# Supplementary material for: Parent-reported child’s close contact with non-household family members and their well-being during the COVID-19 pandemic: A cross-sectional survey
Source: PLoS One. 2023 Oct 19;18(10):e0292344. doi: 10.1371/journal.pone.0292344 (PMC10586646; doi:10.1371/journal.pone.0292344)
Supplement: S1 Text — The information can be downloaded at: DOI 10.18742/21757232. (DOCX) [file pone.0292344.s001.docx]

**S1 Text: Full Survey Material**

D1. Please type your age on your last birthday: [number]

___________

**Screenout if D1 = <18**

Q1. Which country do you live in? [single code]

1. England
2. Scotland
3. Wales
4. Northern Ireland
5. Outside of the UK

**Screenout if Q1 = b-e**

Q2. Which region do you live in? [single code]

1. East Midlands
2. East of England
3. London
4. North East
5. North West
6. South East
7. South West
8. West Midlands
9. Yorkshire and the Humber

Q3. Are you the parent or guardian of a child who usually attends school (that is, before the coronavirus outbreak occurred) and who lives with you? [single code]

1. Yes
2. No

**Screenout if Q3 = b**

Q4. Which of the following age groups do your children who live with you fall into? Please tick any that apply. [multi code]

1. 0-3
2. 4-11
3. 12-18
4. None of the above [exclusive]

**Screenout if b OR c not selected**

Q5. The Government has categorised some people as “critical workers” or “key workers.” For example, this includes people working in health and social care, education, key public services, transport, food distribution and other jobs. As far as you know, are you, or a spouse/partner, a “critical” or “key” worker? Please tick any that apply. [multi code]

1. Yes, me
2. Yes, spouse/partner
3. No [exclusive]
4. Don’t know [exclusive]

IF Q4 = b

Q6. You stated that you have at least one child aged 4-11 who lives with you. Are any of your children who live with you in the following school years? Please tick any that apply. [multi code]

1. Reception
2. Year 1
3. Year 6
4. I have no children in the school years listed above [exclusive]

IF 2 or more options are selected at Q6

Q7. Thinking only about your children in Reception, Year 1 or Year 6, who live with you, which child had the most recent birthday? If the most recent birthday was shared by more than one child (e.g. twins, or children born on the same day in different years), please answer the following questions thinking about one of these children only. [single code]

1. My child in Reception
2. My child in Year 1
3. My child in Year 6

ONLY SHOW OPTIONS SELECTED AT Q6

IF Q6 = a-c

Q8. Can you please tell us how old that child is? [single code]

1. 4
2. 5
3. 6
4. 7
5. 8
6. 9
7. 10
8. 11

IF Q6 = a-c

Q9. Can you please tell us if that child is a: [single code]

1. Boy
2. Girl

IF Q6 = a-c

Q10. Can you please tell us the type of school that child usually attends? [single code]

1. Fee-paying
2. State-funded school
3. Don’t know

IF Q6 = a-c

Q11A. Thinking about that child, have they attended school at all in the past 7 days? [single code]

1. Yes, on 5 days
2. Yes, on 1 to 4 days
3. No
4. Don’t know

IF Q11A = a/b

Q12A. You said that your child has attended school on at least one day in the past week. For which reasons did you send your child to school? Please tick any that apply [multi code]:

RANDOMISE ORDER OF STATEMENTS, anchor other to bottom

1. It is compulsory for my child to attend
2. I need to work
3. I have non-work commitments I need to meet
4. My child’s education will benefit from being at school
5. My child will benefit from seeing their friends
6. Having my child at home is a strain
7. I don’t believe my child will be at risk at school
8. My child wants to go to school
9. I think friends, family or other parents will judge me if I don’t send my child to school
10. It reduces costs at home
11. Other reason [write in]

IF Q11A = b

Q13A. You said that your child has not attended school every day in the past week. For which reasons are they attending only part-time? Please tick any that apply [multi code]:

RANDOMISE ORDER OF STATEMENTS, anchor other to bottom

1. I am using it as childcare and I only need them in part time
2. It is less risky for them to be in part-time
3. The school only offers for them to be in part-time
4. I am only sending them in on days where the lessons are important
5. I am only sending them in on days when their friends are in
6. I am only sending them in on days when I can arrange transport
7. They only live with me part time
8. I started sending them in, but have now changed my mind
9. They have developed coronavirus symptoms (cough or fever, or change in sense of taste or smell)
10. My child developed symptoms of a different illness
11. Someone else in the family developed coronavirus symptoms (cough or fever, or change in sense of taste or smell)
12. Someone else at the school developed coronavirus symptoms (cough or fever, or change in sense of taste or smell)
13. Other reason [write in]

IF Q11A = c

Q14A. You said that your child has not attended school in the past week. For which reasons did you not send your child to school? Please tick any that apply [multi code]:

RANDOMISE ORDER OF STATEMENTS, anchor other to bottom

1. Someone in my household is clinically vulnerable to coronavirus
2. I think it is too risky for my child to attend school at the moment
3. The school is not open
4. The school has asked my child not to attend
5. My child doesn’t want to go
6. I think friends, family or other parents will judge me if I send my child to school
7. I think they will get a better education at home than at school at the moment
8. Being at school will be stressful for my child
9. My child’s friends will not be at school
10. I have another child who can’t go to school
11. I can’t arrange transport to get them to school
12. There’s no point, schools will shut again soon anyway
13. They have developed coronavirus symptoms (cough or fever, or change in sense of taste or smell)
14. My child developed symptoms of a different illness
15. Someone else in the family developed coronavirus symptoms (cough or fever, or change in sense of taste or smell)
16. Someone else at the school developed coronavirus symptoms (cough or fever, or change in sense of taste or smell)
17. Other reason [write in]

IF Q11A = a/b

Q15A. In this question we are interested in things that happened on the most recent day that your child went to school. Please remember that this survey is anonymous – please be honest in your answers. Please tick any that apply. [multi code]

RANDOMISE ORDER OF STATEMENTS, anchor none of these to bottom

1. Either on the way to or from school, or at the school gates, I had physical contact with someone that I don’t live with
2. Either on the way to or from school, or at the school gates, I was within 1 metre of someone that I don’t live with for 1 minute or longer
3. Either on the way to or from school, or at the school gates, I was between 1 and 2 metres of someone that I don’t live with for 15 minutes or longer
4. My child used public transport to get to or from school.
5. My child shared a lift in a car with another family to get to or from school.
6. I gave a lift in a car to a child from another family to get them to or from school.
7. My child washed their hands as soon as they got home from school.
8. My child had a shower or bath as soon as they got home from school.
9. I washed my child’s clothes after they got home from school.
10. None of these [exclusive]

IF Q11A = a/b

Q16A. Thinking about the facilities or procedures at your child’s school. Which of the following, if any, are actually happening as far as you are aware?

Please tick all that apply [multi code]

RANDOMISE ORDER OF STATEMENTS, anchor none of these to bottom

1. There are hand washing facilities or hand gel dispensers at the entrance to the school that are working
2. There are hand washing facilities or hand gel dispensers at the entrance to the classrooms that are working
3. Children’s hand washing or hand gel use is being monitored at school
4. My child’s class sizes is now 15 or fewer
5. Children need to take in their own food and snacks
6. Children are having their temperature checked on the way in
7. The school has used markings or barriers to help children keep their distance from each other
8. None of these are happening at my child’s school [exclusive]

Q17. For the next series of questions, we would like you to think about one of your children who usually goes to school (that is, before the coronavirus outbreak occurred). If you have more than one child who usually went to school, then please answer about your child with the most recent birthday. If the most recent birthday was shared by more than one child (e.g. twins, or children born on the same day in different years), please answer the following questions thinking about one of these children only.

Firstly, can you tell us the first name of your child. This is just so we can refer to them throughout the survey. You can give a fake name if you would prefer. [open]

__________________

Q18. How old is [CHILD]? [single code]

1. 4
2. 5
3. 6
4. 7
5. 8
6. 9
7. 10
8. 11
9. 12
10. 13
11. 14
12. 15
13. 16
14. 17
15. 18

Q19. What year is [CHILD] in at school [single code]

1. Reception
2. Year 1
3. Year 2
4. Year 3
5. Year 4
6. Year 5
7. Year 6
8. Year 7
9. Year 8
10. Year 9
11. Year 10
12. Year 11
13. Year 12
14. Year 13

Q20. Is [CHILD] a: [single code]

1. Boy
2. Girl

Q21. What type of school does [CHILD] normally attend? [single code]

1. Fee-paying
2. State-funded school
3. Don’t know

If Q5 = a/b AND [Q4 ≠ b or Q6 = d)

Q11B. Has [CHILD] attended school at all in the past 7 days? [single code]

1. Yes, on 5 days
2. Yes, on 1 to 4 days
3. No
4. Don’t know

IF Q11B = a/b

Q12B. You said that [CHILD] has attended school on at least one day in the past week. For which reasons did you send [CHILD] to school? Please tick any that apply [multi code]:

RANDOMISE ORDER OF STATEMENTS, anchor other to bottom

1. It is compulsory for my child to attend
2. I need to work
3. I have non-work commitments I need to meet
4. My child’s education will benefit from being at school
5. My child will benefit from seeing their friends
6. Having my child at home is a strain
7. I don’t believe my child will be at risk at school
8. My child wants to go to school
9. I think friends, family or other parents will judge me if I don’t send my child to school
10. It reduces costs at home
11. Other reason [write in]

IF Q11B = b

Q13B. You said that [CHILD] has not attended school every day in the past week. For which reasons is [CHILD] attending only part-time? Please tick any that apply [multi code]:

RANDOMISE ORDER OF STATEMENTS, anchor other to bottom

1. I am using it as childcare and I only need them in part time
2. It is less risky for them to be in part-time
3. The school only offers for them to be in part-time
4. I am only sending them in on days where the lessons are important
5. I am only sending them in on days when their friends are in
6. I am only sending them in on days when I can arrange transport
7. They only live with me part time
8. I started sending them in, but have now changed my mind
9. They have developed coronavirus symptoms (cough or fever, or change in sense of taste or smell)
10. My child developed symptoms of a different illness
11. Someone else in the family developed coronavirus symptoms (cough or fever, or change in sense of taste or smell)
12. Someone else at the school developed coronavirus symptoms (cough or fever, or change in sense of taste or smell)
13. Other reason [write in]

IF Q11B = c

Q14B. You said that [CHILD] has not attended school in the past week. For which reasons did you not send [CHILD] to school? Please tick any that apply [multi code]:

RANDOMISE ORDER OF STATEMENTS, anchor other to bottom

1. Someone in my household is clinically vulnerable to coronavirus
2. I think it is too risky for my child to attend school at the moment
3. The school is not open
4. The school has asked my child not to attend
5. My child doesn’t want to go
6. I think friends, family or other parents will judge me if I send my child to school
7. I think they will get a better education at home than at school at the moment
8. Being at school will be stressful for my child
9. My child’s friends will not be at school
10. I have another child who can’t go to school
11. I can’t arrange transport to get them to school
12. There’s no point, schools will shut again soon anyway
13. They have developed coronavirus symptoms (cough or fever, or change in sense of taste or smell)
14. My child developed symptoms of a different illness
15. Someone else in the family developed coronavirus symptoms (cough or fever, or change in sense of taste or smell)
16. Someone else at the school developed coronavirus symptoms (cough or fever, or change in sense of taste or smell)
17. Other reason [write in]

IF Q11B = a/b

Q15B. In this question we are interested in things that happened on the most recent day that [CHILD] went to school. Please remember that this survey is anonymous – please be honest in your answers. Please tick any that apply. [multi code]

RANDOMISE ORDER OF STATEMENTS, anchor none of these to bottom

1. Either on the way to or from school, or at the school gates, I had physical contact with someone that I don’t live with
2. Either on the way to or from school, or at the school gates, I was within 1 metre of someone that I don’t live with for 1 minute or longer
3. Either on the way to or from school, or at the school gates, I was between 1 and 2 metres of someone that I don’t live with for 15 minutes or longer
4. My child used public transport to get to or from school.
5. My child shared a lift in a car with another family to get to or from school.
6. I gave a lift in a car to a child from another family to get them to or from school.
7. My child washed their hands as soon as they got home from school.
8. My child had a shower or bath as soon as they got home from school.
9. I washed my child’s clothes after they got home from school.
10. None of these [exclusive]

IF Q11B = a/b

Q16B. Thinking about the facilities or procedures at [CHILD]’s school. Which of the following, if any, are actually happening as far as you are aware? Please tick any that apply. [multi code]

RANDOMISE ORDER OF STATEMENTS, anchor none of these to bottom

1. There are hand washing facilities or hand gel dispensers at the entrance to the school that are working
2. There are hand washing facilities or hand gel dispensers at the entrance to the classrooms that are working
3. Children’s hand washing or hand gel use is being monitored at school
4. My child’s class size is now 15 or fewer
5. Children need to take in their own food and snacks
6. Children are having their temperature checked on the way in
7. The school has used markings or barriers to help children keep their distance from each other
8. None of these are happening at [CHILD]’s school [exclusive]

Q22. Does [CHILD] have special educational needs? [singe code]

- Yes
- No
- Don’t know / prefer not to say

Q23. In the past 7 days, how many times, if at all, has [CHILD] left your home for each of the following reasons? [multi code]

1. To go to the shops for groceries, toiletries or medicines
2. To go to the shops for other items
3. For exercise
4. For a medical need (e.g. an outpatient appointment)
5. To go to school
6. To provide help to someone else
7. To meet friends
8. To meet family members who don’t live with you
9. For another reason

Q24. In the past 7 days, have you done the following, because of the risk from coronavirus:

SCALE

- Yes
- No

STATEMENTS – RANDOMISE

1. Washed your hands thoroughly and regularly
2. Stayed 2m (3 steps) away from people you do not live with when outside your home
3. Washed your clothes when you have returned home
4. Washed [CHILD]’s clothes when she/ he has returned home
5. Used tissues or clothing to touch door handles or other objects when outside
6. Cleaned or disinfected items you have brought into the home, such as groceries or parcels
7. Left items that you have brought into the home for 24 hours or more before using them, to kill any virus on them
8. Covered your face or used a face mask when out and about
9. Covered [CHILD’s] face or given her/him a face mask when out and about
10. Worn protective gloves when out and about
11. Made [CHILD] wear protective gloves when out and about

Q25. We are interested in how many people [CHILD] has had close contact with in the past 24 hours. By close contact we mean closer than 2 meters, for fifteen minutes or more. Please remember, this survey is anonymous so please be honest.

In the past 24 hours, how many people in the following categories has [CHILD] had close contact with: [multi code]

1. Someone [CHILD] lives with
2. Friends or other children who [CHILD] does not live with
3. A family member aged under 70 who [CHILD] does not live with
4. A family member aged over 70 who [CHILD] does not live with
5. A babysitter, nanny or childminder
6. Other children, not already reported above
7. Other adults, not already reported above

Q26. Did [CHILD] attend school in the past 24 hours? [single code]

1. Yes
2. No

**RANDOMISE ORDER OF Q27a AND Q27b SHOWN TO EACH RESPONDENT**

Q27a. How much, if at all, do you agree or disagree with the following statements:

STATEMENTS (randomise order):

1. If [CHILD] goes out, she/he is likely to catch coronavirus
2. If [CHILD] goes out, she/he is likely to bring coronavirus back into our home
3. [CHILD] is keeping up with her/his schoolwork
4. I feel confident helping [CHILD] with her/his schoolwork
5. I feel supported by [CHILD’s] school
6. I have access to all the resources that [CHILD] needs to do her/his schoolwork
7. During lockdown, [CHILD] has learned about important things she/he wouldn’t normally learn at school.
8. In the past 7 days, [CHILD] has been bored

SCALE

- Strongly agree
- Tend to agree
- Neither agree nor disagree
- Tend to disagree
- Strongly disagree
- Not applicable

Q27b. How much, if at all, do you agree or disagree with the following statements:

STATEMENTS (randomise order):

1. In the past 7 days, my household has had a regular structure to the day
2. In the past 7 days, [CHILD] has kept in touch with her/his friends
3. [CHILD] is worried about coronavirus
4. In the past 7 days, [CHILD] has felt upset about not seeing other family members who do not live with us
5. In the past 7 days, I have found it hard to keep up with work or other important commitments
6. In the past 7 days, people in my household have been getting along well
7. I am worried about the financial impact of lockdown measures
8. Before the school closures, [CHILD] had extra support at school

SCALE

- Strongly agree
- Tend to agree
- Neither agree nor disagree
- Tend to disagree
- Strongly disagree
- Not applicable

Q28. To what extent, if at all, do you agree or disagree with the following statements?

RANDOMISE ORDER OF STATEMENTS

1. If it were possible, I would feel comfortable sending [CHILD] to school next week.
2. If it were possible, I would want to send [CHILD] to school next week.
3. There is still too much coronavirus around for schools to be able to reopen safely
4. When [CHILD’s] school fully reopens, some parents will send their children to school, even if they have symptoms of coronavirus
5. It is impossible for children to maintain ‘social distancing’ at school
6. Children will not wash their hands properly at school
7. Staff will not wash their hands properly at school
8. Schools will not be able to provide good quality education at the moment
9. School will not be an enjoyable place for [CHILD] to be at the moment

SCALE

- Strongly agree
- Tend to agree
- Neither agree nor disagree
- Tend to disagree
- Strongly disagree
- Not applicable

Q29A. Has [CHILD] had any of the following symptoms in the past 7 days? Please tick any that apply, no matter where you think the symptoms came from, and even if the symptoms were mild. [multi code]

RANDOMISE ORDER OF STATEMENTS, anchor “none of these” and “don’t know” to bottom

- New, continuous cough
- High temperature / fever
- Runny nose
- Diarrhoea
- Nausea / feeling sick
- Vomiting
- Sneezing
- Loss of appetite
- Loss of sense of smell (fully or partial)
- Loss of taste
- None of these
- Don’t know

Q29B. Have you, or anyone else in your household (excluding [CHILD]) had any of the following symptoms in the past 14 days? Please tick any that apply, no matter where you think the symptoms came from, and even if the symptoms were mild. [multi code]

RANDOMISE ORDER OF STATEMENTS, anchor “none of these” and “don’t know” to bottom

- New, continuous cough
- High temperature / fever
- Runny nose
- Diarrhoea
- Nausea / feeling sick
- Vomiting
- Sneezing
- Loss of appetite
- Loss of sense of smell (fully or partial)
- Loss of taste
- None of these
- Don’t know

Q30. Do you know if [CHILD] has had, or currently has, coronavirus? (Please select the option that BEST applies) [single code]

They have definitely had it or definitely have it now

They have probably had it or probably have it now

They have probably not had it and probably don’t have it now

1. They have definitely not had it and definitely don’t have it now

**RANDOMISE ORDER OF Q31a AND Q31b SHOWN TO EACH RESPONDENT**

Q31a. Please tell us how often each of these things happen to [CHILD]. There are no right or wrong answers.

*SCALE*

- *Never*
- *Sometimes*
- *Often*
- *Always*

STATEMENTS

- My child worries about things

- My child worries that something awful will happen to someone in the family

- My child worries that bad things will happen to him/her

- My child feels sad or empty

- Nothing is much fun for my child anymore

- My child has trouble sleeping

- My child has problems with his/her appetite

- My child has no energy for things

Q31b. Please tell us how often each of these things happen to [CHILD]. There are no right or wrong answers.

*SCALE*

- *Never*
- *Sometimes*
- *Often*
- *Always*

STATEMENTS

- My child worries that something bad will happen to him/her

- My child worries about what is going to happen

- My child thinks about death

- My child is tired a lot

- My child cannot think clearly

- My child feels worthless

- My child feels like he/she doesn’t want to move

- My child feels restless

Q32. Thinking about next flu season (September 2020 – March 2021). During this time, you may be invited to vaccinate [CHILD] against flu. All primary school, but not secondary school children, are eligible for the child flu vaccine. For the following questions, please think about the next flu season.

For each of the following statements, please tell us to what extent, if at all, you agree or disagree:

I want [CHILD] to be vaccinated for flu next year (2020/21)

- Strongly agree
- Agree
- Neither agree nor disagree
- Disagree
- Strongly disagree
- Not applicable, [CHILD] will be in secondary school in next school year (2020/21)

IF Q32 = a-e

Q33 I intend [CHILD] to be vaccinated for flu next year (2020/21)

- Strongly agree
- Agree
- Neither agree nor disagree
- Disagree
- Strongly disagree
- Not applicable, [CHILD] will be in secondary school in next school year (2020/21)

Thinking now about yourself…

Q34. Over the last 2 weeks, how often have you been bothered by the following problems?

SCALE

- Not at all
- Several days
- More than half the days
- Nearly every day

STATEMENTS

- Feeling nervous, anxious or on edge
- Not being able to stop or control worrying
- Little interest or pleasure in doing things
- Feeling down, depressed, or hopeless

And finally, the following questions ask for some more information about you and your household.

D2. Which gender do you identify yourself with? [single code]

- Male
- Female
- Prefer to self-describe
- Prefer not to say

D3. What is your employment status? [single code]

1. Full time paid job (31+ hours)
2. Part time paid job (<31 hours)
3. Doing paid work on a self-employed basis or within your own business
4. Employed, but currently furloughed
5. Student / On a government training programme (Nation Traineeship/Modern Apprenticeship)
6. Out of work (6 months or less)
7. Out of work (more than 6 months)
8. Looking after home / Homemaker
9. Retired
10. Disabled OR Long-term sick
11. Unpaid work for a business, community or voluntary organisation
12. Prefer not to say

If answer a/b/c to D3

D4. Are you currently working from home? [single code]

- Yes
- No

D5. What is the highest level of educational qualification you have received? [single code]

1. PhD/Doctor
2. Master’s
3. Bachelor’s Degree or equivalent (Such as a NVQ level 5)
4. Higher education (Such as a HND or a NVQ level 4)
5. A level or equivalent (Such as Scottish Highers or NVQ level 3)
6. GCSE and below (Such as O level or an RSA Diploma)
7. Other qualifications (Such as NVQ level 1)
8. No qualifications
9. Prefer not to say

D6. Which of the following income brackets best represents your household income, before tax deductions for income tax, National Insurance etc? [single code]

1. Less than £5,000
2. £5,000-£9,999
3. £10,000-£14,999
4. £15,000-£19,999
5. £20,000-£24,999
6. £25,000-£29,999
7. £30,000-£34,999
8. £35,000-£39,999
9. £40,000-£44,999
10. £45,000-£49,999
11. £50,000-£59,999
12. £60,000-£69,999
13. £70,000-£84,999
14. £85,000-£99,999
15. More than £100,000
16. Prefer not to say

D7. What is your current marital or civil partnership status? [single code]

1. Single (i.e. never married and never registered as a same sex civil-partnership)
2. Co-habiting with partner (but never married or been in a civil partnership)
3. Civil partnership
4. Married
5. Separated, but still legally married / in a civil partnership
6. Divorced / Civil partnership legally dissolved
7. Widowed / Surviving partner from a same-sex civil partnership

D8. Which of the following categories would best describe your ethnicity? [single code]

1. English/Welsh/Scottish/Northern Irish/British
2. Irish
3. Gypsy or Irish Traveller
4. Other
5. White and Black Caribbean
6. White and Black African
7. White and Asian
8. Other
9. Indian
10. Pakistani
11. Bangladeshi
12. Chinese
13. Other
14. Caribbean
15. African
16. Other
17. Arab
18. Any other (please specify)
19. Prefer not to say

D10. How many children in each age group live in your household? [number list]

- 0-3
- 4-10
- 11-15
- 16-18

Allow numerical input for each age group

D11. Do you live with anyone over the age of 70 years? [single code]

1. Yes
2. No

D12. Including yourself, how many people live in your household? [single code]

1. 2
2. 3
3. 4
4. 5
5. 6
6. 7
7. 8
8. 9
9. 10 or more

D9. Do any of the following have a medical condition than might make them particularly vulnerable to coronavirus?

SCALE

- Yes
- No
- Don’t know

STATEMENTS

1. Yourself
2. [CHILD]
3. Anyone else you live with [show if D12 = b-i]

D13. [Record number] How many bedrooms does your home have?

D14. [Multi code] Which of the following do you have access to at your home:

1. A garden
2. A patio / terrace
3. A balcony
4. Another form of outdoor space
5. No outdoor space

Thank you for completing this survey. If you have any concerns about your child’s mental health, please click [here](https://youngminds.org.uk/).
