# Supplementary material for: Parent-reported child’s close contact with non-household family members and their well-being during the COVID-19 pandemic: A cross-sectional survey
Source: PLoS One. 2023 Oct 19;18(10):e0292344. doi: 10.1371/journal.pone.0292344 (PMC10586646; doi:10.1371/journal.pone.0292344)
Supplement: S1 Appendix — (DOCX) [file pone.0292344.s002.docx]

# Appendix A

Table 7 Binary logistic regression comparing parent and child characteristics and associations with children’s non-household close contact

| Variable | Level | Child’s not had close contact, n, (%) | Child’s had close contact, n, (%) |
| --- | --- | --- | --- |
| Parent gender | Male | 797 (86) | 134 (14) |
|  | Female | 894 (84) | 171 (16) |
| Parent age | 18-35 years | 305 (77) | 89 (23) |
|  | 36-45 years | 738 (85) | 130 (15) |
|  | 46 years ≥ | 658 (88) | 90 (12) |
| Region | East Midlands | 129 (85) | 22 (15) |
|  | East of England | 186 (85) | 33 (15) |
|  | North East | 104 (88) | 14 (12) |
|  | North West | 232 (85) | 41 (15) |
|  | South East | 283 (84) | 52 (16) |
|  | South West | 143 (88) | 20 (12) |
|  | West Midlands | 183 (87) | 28 (13) |
|  | Yorkshire & the Humber | 161 (83) | 34 (17) |
|  | London | 280 (81) | 65 (19) |
| Household income | ≤ £34,999 | 660 (82) | 144 (18) |
|  | £35,000 ≥ | 958 (86) | 156 (14) |
| Employment status^1^ | Working | 1412 (84) | 265 (16) |
|  | Not working | 278 (87) | 43 (13) |
| Parent Working from home^2^ | Yes | 774 (82) | 166 (18) |
|  | No | 551 (88) | 77 (12) |
| Education level | ≤ A-level | 740 (87) | 115 (13) |
|  | Degree ≥ | 947 (83) | 192 (17) |
| Marital status | Living alone | 262 (80) | 65 (20) |
|  | Married/cohabiting | 1439 (86) | 244 (14) |
| Ethnicity | White | 1483 (85) | 270 (15) |
|  | BAME | 203 (84) | 38 (16) |
| Key worker status | Both parents | 167 (86) | 28 (14) |
|  | One parent | 686 (81) | 161 (19) |
|  | No | 837 (88) | 119 (12) |
| Child gender | Boy | 891 (84) | 172 (16) |
|  | Girl | 810 (86) | 137 (14) |
| Child school year | Early Years | 85 (84) | 16 (16) |
|  | Key Stage 1 | 279 (78) | 77 (22) |
|  | Key Stage 2 | 576 (85) | 99 (15) |
|  | Key Stage 3 | 334 (85) | 61 (15) |
|  | Key Stage 4 | 289 (88) | 39 (12) |
|  | Years 12 & 13 | 138 (89) | 17 (11) |
| Child had special educational needs | Yes | 118 (73) | 43 (27) |
|  | No | 1568 (86) | 264 (14) |
| Child lower well-being | Yes | 384 (74) | 135 (26) |
|  | No | 1317 (88) | 174 (12) |
| Parent lower well-being | Yes | 295 (75) | 96 (25) |
|  | No | 1406 (87) | 213 (13) |
| Child vulnerable COVID-19 | Yes | 111 (71) | 46 (29) |
|  | No | 1566 (86) | 260 (14) |
| Household vulnerable COVID-19 | Yes | 429 (84) | 81 (16) |
|  | No | 1127 (86) | 184 (14) |
| Someone over 70 years | Yes | 59 (68) | 28 (32) |
|  | No | 1642 (85) | 281 (15) |
| COVID-19 behaviours followed by child and parent | Continuous (0=followed no behaviours, 11=followed all behaviours) | N=1701,  M=5.51,  SD=3.06 | N=309,  M=6.11,  SD=3.27 |
| Access to outside space^3^ | Garden | 1524 (85) | 260 (15) |
|  | Other^3^ | 121 (77) | 36 (23) |
|  | No | 56 (81) | 13 (19) |

^1^ Working includes students and volunteers.

^2^ Question only offered to participants who reported working in a paid job (full-time, part-time, and self-employed) and not to participants who reported being a student, on furlough and a volunteer.

^3^ Participants that reported no access to a garden but had access to other outdoor spaces such as patio, terrace, and balcony.

Abbreviations: n = number; % = percentage; M = mean; SD = standard deviation.

# Appendix B

**Table 8 Binary logistic regression comparing statements about lockdown and associations with children’s non-household close contact. Close contact is defined by a child’s close contact with a family member outside the household (n=309)**

| Statement | Level | Child’s not had close contact, n, (%) | Child’s had close contact, n, (%) |
| --- | --- | --- | --- |
| If child goes out, she/he is likely to catch coronavirus | 5-point Likert-scale (1=strongly agree, 5=strongly disagree) | N=1672,  M=3.27,  SD=1.03 | N=309,  M=3.08,  SD=1.18 |
| If child goes out, she/he is likely to bring coronavirus back into our home | 5-point Likert-scale (1=strongly agree, 5=strongly disagree) | N=1672,  M=3.23,  SD=1.07 | N=309,  M=3.05,  SD=1.16 |
| Child is keeping up with his/her schoolwork | 5-point Likert-scale (1=strongly agree, 5=strongly disagree) | N=1672,  M=2.12,  SD=1.08 | N=309,  M=2.30,  SD=1.15 |
| I feel confident helping child with her/his schoolwork | 5-point Likert-scale (1=strongly agree, 5=strongly disagree) | N=1672,  M=2.23,  SD=1.03 | N=305,  M=2.30,  SD=1.07 |
| I feel supported by child’s school | 5-point Likert-scale (1=strongly agree, 5=strongly disagree) | N=1681,  M=2.32,  SD=1.07 | N=306,  M=2.32,  SD=1.07 |
| I have access to all the resources that child needs to do her/his schoolwork | 5-point Likert-scale (1=strongly agree, 5=strongly disagree) | N=1678,  M=2.15,  SD=1.02 | N=307,  M=2.21,  SD=1.03 |
| During lockdown, child has learned about important things she/he wouldn’t normally learn at school | 5-point Likert-scale (1=strongly agree, 5=strongly disagree) | N=1691,  M=2.40,  SD=1.00 | N=308,  M=2.38,  SD=1.05 |
| In the past 7 days, child has been bored | 5-point Likert-scale (1=strongly agree, 5=strongly disagree) | N=1694,  M=2.54,  SD=1.20 | N=309,  M=2.30,  SD=1.16 |
| In the past 7 days, my household has had a regular structure to the day | 5-point Likert-scale (1=strongly agree, 5=strongly disagree) | N=1692,  M=2.31,  SD=1.02 | N=309,  M=2.43,  SD=1.12 |
| In the past 7 days, child has kept in touch with her/his friends | 5-point Likert-scale (1=strongly agree, 5=strongly disagree) | N=1691,  M=2.25,  SD=1.14 | N=307,  M=2.26,  SD=1.12 |
| Child is worried about coronavirus | 5-point Likert-scale (1=strongly agree, 5=strongly disagree) | N=1690,  M=2.72,  SD=1.12 | N=306,  M=2.70,  SD=1.23 |
| In the past 7 days, child has felt upset about not seeing other family members who do not live with us | 5-point Likert-scale (1=strongly agree, 5=strongly disagree) | N=1684,  M=2.97,  SD=1.24 | N=308,  M=2.65,  SD=1.25 |
| In the past 7 days, I have found it hard to keep up with work or other important commitments | 5-point Likert-scale (1=strongly agree, 5=strongly disagree) | N=1626,  M=3.22,  SD=1.21 | N=299,  M=2.79,  SD=1.21 |
| In the past 7 days, people in my household have been getting along well | 5-point Likert-scale (1=strongly agree, 5=strongly disagree) | N=1691,  M=2.08,  SD=0.95 | N=309,  M=2.19,  SD=1.01 |
| I am worried about the financial impact of lockdown measures | 5-point Likert-scale (1=strongly agree, 5=strongly disagree) | N=1685,  M=2.51,  SD=1.21 | N=304,  M=2.40,  SD=1.16 |
| Before the school closures, child had extra support at school | 5-point Likert-scale (1=strongly agree, 5=strongly disagree) | N=1528,  M=3.59,  SD=1.36 | N=285,  M=3.16,  SD=1.48 |

Abbreviations: n = number; % = percentage; M = mean; SD = standard deviation.

# Appendix C

**Table 9 Frequencies to show the behaviours that parents or children had followed, in the past 7 days because of the risk of coronavirus. The findings our presented by children’s non-household family close contact (n=309) and lower well-being (n=519).**

| Statements | Statement in relation to COVID-19 guidelines at the time | Total participants (n=2010, %) | Childs not had close contact (n=1701, %) | Childs had close contact (n=309, (%) | Child higher well-being, (n=1491, %) | Child lower well-being, (n=519, %) |
| --- | --- | --- | --- | --- | --- | --- |
| Washed your hands thoroughly and regularly | Recommended | 1895 (94%) | 1617 (95) | 278 (90) | 1435 (96) | 460 (89) |
| Stayed 2m (3 steps) away from people you do not live with when outside your home | Recommended | 1867 (93%) | 1599 (94) | 268 (87) | 1413 (95) | 454 (87) |
| Cleaned or disinfected items you have brought into the home, such as groceries or parcels | Not recommended | 1089 (54%) | 908 (53) | 181 (59) | 779 (52) | 310 (60) |
| Washed [CHILD]’s clothes when she/ he has returned home | Not recommended | 1089 (54%) | 829 (49) | 176 (57) | 688 (46) | 317 (61) |
| Washed your clothes when you have returned home | Not recommended | 959 (48%) | 788 (46) | 171 (55) | 653 (44) | 306 (59) |
| Used tissues or clothing to touch door handles or other objects when outside | Not recommended | 954 (48%) | 788 (46) | 166 (54) | 679 (46) | 275 (53) |
| Covered your face or used a face mask when out and about | Not recommended | 822 (41%) | 678 (40) | 144 (47) | 571 (38) | 251 (48) |
| Covered [CHILD’s] face or given her/him a face mask when out and about | Not recommended | 741 (37%) | 600 (35) | 141 (46) | 500 (34) | 241 (46) |
| Left items that you have brought into the home for 24 hours or more before using them, to kill any virus on them | Not recommended | 725 (36%) | 607 (36) | 118 (38) | 493 (33) | 232 (45) |
| Worn protective gloves when out and about | Not recommended | 693 (35%) | 562 (33) | 131 (43) | 453 (30) | 240 (46) |
| Made [CHILD] wear protective gloves when out and about | Not recommended | 503 (25%) | 390 (23) | 113 (37) | 308 (21) | 195 (38) |

Abbreviations: n = number of parents; % = percentage.

# Appendix D

**Table 10 Binary logistic regression comparing parent and child characteristics and associations with children’s lower well-being (n=519)**

| Variable | Level | Child higher well-being, n, (%) | Child lower well-being, n, (%) |
| --- | --- | --- | --- |
| Parent gender | Male | 702 (75) | 229 (25) |
|  | Female | 784 (74) | 281 (26) |
| Parent age | 18-35 years | 263 (67) | 131 (33) |
|  | 36-45 years | 652 (75) | 216 (25) |
|  | 46 years ≥ | 576 (77) | 172 (23) |
| Region | East Midlands | 101 (67) | 50 (33) |
|  | East of England | 163 (74) | 56 (26) |
|  | North East | 92 (78) | 26 (22) |
|  | North West | 208 (76) | 65 (24) |
|  | South East | 248 (74) | 87 (26) |
|  | South West | 118 (72) | 45 (28) |
|  | West Midlands | 161 (76) | 50 (24) |
|  | Yorkshire & the Humber | 152 (78) | 43 (22) |
|  | London | 248 (72) | 97 (28) |
| Household income | ≤ £34,999 | 583 (73) | 221 (27) |
|  | £35,000 ≥ | 832 (75) | 282 (25) |
| Employment status^1^ | Working | 1240 (74) | 437 (26) |
|  | Not working | 244 (76) | 77 (24) |
| Parent Working from home^2^ | Yes | 682 (73) | 258 (27) |
|  | No | 474 (75) | 154 (25) |
| Education level | ≤ A-level | 642 (75) | 213 (25) |
|  | Degree ≥ | 836 (73) | 303 (27) |
| Marital status | Living alone | 234 (72) | 93 (28) |
|  | Married/cohabiting | 1257 (75) | 426 (25) |
| Ethnicity | White | 1304 (74) | 449 (26) |
|  | BAME | 176 (73) | 65 (27) |
| Key worker status | Both parents | 134 (69) | 61 (31) |
|  | One parent | 593 (70) | 254 (30) |
|  | No | 754 (79) | 202 (21) |
| Child gender | Boy | 771 (73) | 292 (27) |
|  | Girl | 720 (76) | 227 (24) |
| Child school year | Early Years | 77 (76) | 24 (24) |
|  | Key Stage 1 | 263 (74) | 93 (26) |
|  | Key Stage 2 | 517 (7) | 158 (23) |
|  | Key Stage 3 | 286 (72) | 109 (28) |
|  | Key Stage 4 | 230 (70) | 98 (30) |
|  | Years 12 & 13 | 118 (76) | 37 (24) |
| Child has special educational needs | Yes | 71 (44) | 90 (56) |
|  | No | 1410 (770 | 422 (23) |
| Parent low well-being | Yes | 159 (41) | 232 (59) |
|  | No | 1332 (82) | 287 (18) |
| Child vulnerable COVID-19 | Yes | 77 (49) | 80 (51) |
|  | No | 1393 (76) | 433 (24) |
| Household vulnerable COVID-19 | Yes | 329 (65) | 181 (35) |
|  | No | 1029 (78) | 282 (22) |
| Someone over 70 years | Yes | 46 (53) | 41 (47) |
|  | No | 1445 (75) | 478 (25) |
| COVID-19 behaviours followed by child and parent | Continuous (0=followed no behaviours, 11=followed all behaviours) | N=1491,  M=5.35,  SD=3.02 | N=519,  M=6.32,  SD=3.22 |
| Access to outside space^3^ | Garden | 1333 (75) | 451 (25) |
|  | Other^3^ | 108 (69) | 49 (31) |
|  | No | 50 (72) | 19 (28) |

* *p≤ ·05* and formatted bold.
** *p≤ ·001* and formatted bold.

Some results are rounded to 3 rather than 2 decimal places to distinguish between *p≤ ·05* and *p≤ ·001.*

† When interpretating the predictor listed in the row, we controlled for the other variables listed here: participant gender, age, region, household income, employment status, education level, marital status, ethnicity, and the child’s gender and school year.

^^^ Not included variable highly correlated with employment variable.

^1^ Working includes students and volunteers.

^2^ Question only offered to participants who reported working in a paid job (full-time, part-time, and self-employed) and not to participants who reported being a student, on furlough and a volunteer.

^3^ Participants that reported no access to a garden but had access to other outdoor spaces such as patio, terrace, and balcony.

Abbreviations: n = number of parents; % = percentage; CI = confidence interval.

# Appendix E

**Table 11 Binary logistic regression comparing statements about lockdown and associations with children’s lower well-being (n=519)**

| Statement | Level | Child higher well-being, n, (%) | Child lower well-being, n, (%) |
| --- | --- | --- | --- |
| If child goes out, she/he is likely to catch coronavirus | 5-point Likert-scale (1=strongly agree, 5=strongly disagree) | N=1473,  M=3.37,  SD=1.02 | N=515,  M=2.87,  SD=1.06 |
| If child goes out, she/he is likely to bring coronavirus back into our home | 5-point Likert-scale (1=strongly agree, 5=strongly disagree) | N=1465,  M=3.33,  SD=1.04 | N=515,  M=2.82  SD=1.11 |
| Child is keeping up with his/her schoolwork | 5-point Likert-scale (1=strongly agree, 5=strongly disagree) | N=1470,  M=2.03,  SD=1.01 | N=508,  M=2.48,  SD=1.24 |
| I feel confident helping child with her/his schoolwork | 5-point Likert-scale (1=strongly agree, 5=strongly disagree) | N=1469,  M=2.16,  SD=0.98 | N=508,  M=2.48,  SD=1.13 |
| I feel supported by child’s school | 5-point Likert-scale (1=strongly agree, 5=strongly disagree) | N=1475,  M=2.25,  SD=1.03 | N=512,  M=2.52  SD=1.15 |
| I have access to all the resources that child needs to do her/his schoolwork | 5-point Likert-scale (1=strongly agree, 5=strongly disagree) | N=1473,  M=2.09,  SD=0.97 | N=512,  M=2.37,  SD=1.14 |
| During lockdown, child has learned about important things she/he wouldn’t normally learn at school | 5-point Likert-scale (1=strongly agree, 5=strongly disagree) | N=1481,  M=2.37,  SD=0.98 | N=518,  M=2.49,  SD=1.08 |
| In the past 7 days, child has been bored | 5-point Likert-scale (1=strongly agree, 5=strongly disagree) | N=1486,  M=2.63,  SD=1.20 | N=517,  M=2.14,  SD=1.11 |
| In the past 7 days, my household has had a regular structure to the day | 5-point Likert-scale (1=strongly agree, 5=strongly disagree) | N=1484,  M=2.27,  SD=0.99 | N=517,  M=2.50,  SD=1.14 |
| In the past 7 days, child has kept in touch with her/his friends | 5-point Likert-scale (1=strongly agree, 5=strongly disagree) | N=1483,  M=2.21,  SD=1.12 | N=515,  M=2.39,  SD=1.19 |
| Child is worried about coronavirus | 5-point Likert-scale (1=strongly agree, 5=strongly disagree) | N=1481,  M=2.88,  SD=1.12 | N=515,  M=2.27,  SD=1.08 |
| In the past 7 days, child has felt upset about not seeing other family members who do not live with us | 5-point Likert-scale (1=strongly agree, 5=strongly disagree) | N=1475,  M=3.09,  SD=1.24 | N=517,  M=2.45,  SD=1.15 |
| In the past 7 days, I have found it hard to keep up with work or other important commitments | 5-point Likert-scale (1=strongly agree, 5=strongly disagree) | N=1429,  M=3.37,  SD=1.19 | N=496,  M=2.55,  SD=1.15 |
| In the past 7 days, people in my household have been getting along well | 5-point Likert-scale (1=strongly agree, 5=strongly disagree) | N=1484,  M=2.00,  SD=0.93 | N=516,  M=2.37,  SD=0.97 |
| I am worried about the financial impact of lockdown measures | 5-point Likert-scale (1=strongly agree, 5=strongly disagree) | N=1475,  M=2.58,  SD=1.23 | N=514,  M=2.24,  SD=1.10 |
| Before the school closures, child had extra support at school | 5-point Likert-scale (1=strongly agree, 5=strongly disagree) | N=1326,  M=3.72,  SD=1.32 | N=487,  M=2.98,  SD=1.42 |

Abbreviations: N = number of parents; M = mean; SD = standard deviation; % = percentage.
