# Supplementary material for: Parent-reported child’s close contact with non-household family members and their well-being during the COVID-19 pandemic: A cross-sectional survey
Source: PLoS One. 2023 Oct 19;18(10):e0292344. doi: 10.1371/journal.pone.0292344 (PMC10586646; doi:10.1371/journal.pone.0292344)
Supplement: Attachment — Submitted filename: PONE-D-23-01008 + Reviewed.pdf [file pone.0292344.s003.pdf]

PLOS ONE

Children’s interactions with non-household family members and child well-being during the COVID-19 pandemic: a cross-sectional survey

--Manuscript Draft--

**Manuscript Number:**	PONE-D-23-01008
**Article Type:**	Research Article
**Full Title:**	Children’s interactions with non-household family members and child well-being during the COVID-19 pandemic: a cross-sectional survey
**Short Title:**	Children’s interactions with non-household family members and their well-being during COVID-19
**Corresponding Author:**	Lisa Woodland<br>King's College London<br>London, London UNITED KINGDOM
**Keywords:**	children; COVID-19; close contact; physical interactions; adherence; England; parents; well-being; mental health; physical health; exercise
**Abstract:**	In England (UK), at the start of the COVID-19 pandemic, schools were closed, and the public were required to reduce social interactions to slow the spread of COVID-19. We investigated the risk factors for children having, 1) close contact with family members from outside the household (non-adherent behaviour) and 2) low well-being (Revised Child Anxiety and Depression Scale). We conducted a cross-sectional survey between 8 and 11 June 2020 in parents (n = 2,010) who were aged eighteen years or over and had a school-aged child (4-18 years old). Parents reported that 15% (n = 309) of children had contact with a family member they did not live with and that 26% (n = 519) had low well-being. Children were more likely to have non-household family interactions when they had special educational needs, had a lower well-being, were vulnerable to COVID-19, lived with someone who was over 70 years of age and their parent had low well-being. Children were more likely to have a lower well-being when they had special educational needs, were vulnerable to COVID-19, lived with someone else who was also vulnerable to COVID-19, or lived with someone that was over 70 years old.
**Order of Authors:**	Lisa Woodland
	Louise E Smith
	Samantha K Brooks
	Rebecca K Webster
	Richard Amlôt
	Antonia Rubin
	G James Rubin
**Opposed Reviewers:**	
**Additional Information:**	
**Question**	**Response**
**Financial Disclosure**<br><br>Enter a financial disclosure statement that describes the sources of funding for the work included in this submission. Review the submission guidelines for detailed requirements. View published research articles from *PLOS ONE* for specific examples.	Funding: This study was funded by the Economic and Social Research Council [grant number ES/P000703/1] and by the National Institute for Health and Care Research Health Protection Research Unit (NIHR HPRU) in Emergency Preparedness and Response [grant number NIHR200890], a partnership between the UK Health Security Agency, King’s College London and the University of East Anglia. The views expressed are those of the author(s) and not necessarily those of the NIHR, UKHSA or the Department of Health and Social Care. For the purpose of open access, the author has applied a Creative Commons Attribution (CC BY) licence to any Author Accepted Manuscript version arising

Powered by Editorial Manager® and ProduXion Manager® from Aries Systems Corporation

This statement is required for submission and **will appear in the published article** if the submission is accepted. Please make sure it is accurate.<br><br>**Unfunded studies**<br>Enter: *The author(s) received no specific funding for this work.*<br><br>**Funded studies**<br>Enter a statement with the following details:<br>• Initials of the authors who received each award<br>• Grant numbers awarded to each author<br>• The full name of each funder<br>• URL of each funder website<br>• Did the sponsors or funders play any role in the study design, data collection and analysis, decision to publish, or preparation of the manuscript?<br>• **NO** - Include this sentence at the end of your statement: *The funders had no role in study design, data collection and analysis, decision to publish, or preparation of the manuscript.*<br>• **YES** - Specify the role(s) played.<br><br>* typeset	Role of the funding source: The funders had no role in study design, data collection and analysis, decision to publish, or preparation of the manuscript. The corresponding author had full access to all the data and had final responsibility for the decision to submit for publication.
**Competing Interests**<br><br>Use the instructions below to enter a competing interest statement for this submission. On behalf of all authors, disclose any competing interests that could be perceived to bias this work—acknowledging all financial support and any other relevant financial or non-financial competing interests.<br><br>This statement is required for submission and **will appear in the published article** if the submission is accepted. Please make sure it is accurate and that any funding sources listed in your Funding Information later in the submission form are also declared in your Financial Disclosure statement.<br><br>View published research articles from	I have read the journal's policy and the authors of this manuscript have the following competing interests: G.J.R., R.A. and L.S. participate in the UK's Scientific Advisory Group for Emergencies, or its subgroups. RA is an employee of the UK Health Security Agency. These groups did not fund the study or authors. The author interests do not alter our adherence to PLOS ONE policies on sharing data and materials.

Powered by Editorial Manager® and ProduXion Manager® from Aries Systems Corporation

PLOS ONE for specific examples.<br><br>**NO authors have competing interests**<br><br>Enter: *The authors have declared that no competing interests exist.*<br><br>**Authors with competing interests**<br><br>Enter competing interest details beginning with this statement:<br><br>*I have read the journal's policy and the authors of this manuscript have the following competing interests: [insert competing interests here]*<br><br>* typeset	
**Ethics Statement**<br><br>Enter an ethics statement for this submission. This statement is required if the study involved:<br><br>• Human participants<br>• Human specimens or tissue<br>• Vertebrate animals or cephalopods<br>• Vertebrate embryos or tissues<br>• Field research<br><br>Write "N/A" if the submission does not require an ethics statement.<br><br>General guidance is provided below. Consult the submission guidelines for detailed instructions. **Make sure that all information entered here is included in the Methods section of the manuscript.**	The research was approved by the Psychiatry, Nursing and Midwifery Research Ethics Subcommittee at King's College London (LRS-19/20-18787).

Powered by Editorial Manager® and ProduXion Manager® from Aries Systems Corporation

**Format for specific study types**<br><br>**Human Subject Research (involving human participants and/or tissue)**<br>• Give the name of the institutional review board or ethics committee that approved the study<br>• Include the approval number and/or a statement indicating approval of this research<br>• Indicate the form of consent obtained (written/oral) or the reason that consent was not obtained (e.g. the data were analyzed anonymously)<br><br>**Animal Research (involving vertebrate animals, embryos or tissues)**<br>• Provide the name of the Institutional Animal Care and Use Committee (IACUC) or other relevant ethics board that reviewed the study protocol, and indicate whether they approved this research or granted a formal waiver of ethical approval<br>• Include an approval number if one was obtained<br>• If the study involved *non-human primates*, add *additional details* about animal welfare and steps taken to ameliorate suffering<br>• If anesthesia, euthanasia, or any kind of animal sacrifice is part of the study, include briefly which substances and/or methods were applied<br><br>**Field Research**<br><br>Include the following details if this study involves the collection of plant, animal, or other materials from a natural setting:<br>• Field permit number<br>• Name of the institution or relevant body that granted permission	
**Data Availability**<br><br>Authors are required to make all data underlying the findings described fully available, without restriction, and from the time of publication. PLOS allows rare exceptions to address legal and ethical concerns. See the PLOS Data Policy and FAQ for detailed information.	Yes - all data are fully available without restriction

A Data Availability Statement describing where the data can be found is required at submission. Your answers to this question constitute the Data Availability Statement and **will be published in the article**, if accepted.<br><br>**Important:** Stating 'data available on request from the author' is not sufficient. If your data are only available upon request, select 'No' for the first question and explain your exceptional situation in the text box.<br><br>Do the authors confirm that all data underlying the findings described in their manuscript are fully available without restriction?	
**Describe where the data may be found in full sentences. If you are copying our sample text, replace any instances of XXX with the appropriate details.**<br><br>• If the data are **held or will be held in a public repository**, include URLs, accession numbers or DOIs. If this information will only be available after acceptance, indicate this by ticking the box below. For example: *All XXX files are available from the XXX database (accession number(s) XXX, XXX.*).<br>• If the data are all contained **within the manuscript and/or Supporting Information files**, enter the following: *All relevant data are within the manuscript and its Supporting Information files.*<br>• If neither of these applies but you are able to provide **details of access elsewhere**, with or without limitations, please do so. For example:<br><br>*Data cannot be shared publicly because of [XXX]. Data are available from the XXX Institutional Data Access / Ethics Committee (contact via XXX) for researchers who meet the criteria for access to confidential data.*<br><br>*The data underlying the results presented in the study are available from (include the name of the third party*	The data set and survey questions related to this study can be accessed via the King's College London research data repository, KORDS (DOI 10.18742/21757232). For further information contact: kcl - research.data research.data@kcl.ac.uk.

and contact information or URL).<br>• This text is appropriate if the data are owned by a third party and authors do not have permission to share the data.<br><br>* typeset	
Additional data availability information:	Tick here if the URLs/accession numbers/DOIs will be available only after acceptance of the manuscript for publication so that we can ensure their inclusion before publication.

Powered by Editorial Manager® and ProduXion Manager® from Aries Systems Corporation

Lisa Woodland
King's College London
Cutcombe Road,
London
SE5 9RJ

Thursday, 12 January 2023

Dear Dr Chenette,

We are pleased to submit this research article entitled "Children's interactions with non-household family members and child well-being during the covid-19 pandemic: a cross-sectional survey" for consideration by your journal.

We confirm that this work is original and has not been published elsewhere, nor is it currently under consideration for publication elsewhere and will not be submitted to any other journal while under consideration by PLOS ONE.

In this paper, we show the risk factors associated with children having a low well-being during the COVID-19 school closures. We also show that worryingly, children were non-adherent to having physical interactions with non-household family members, as such increasing the risk of disease transmission in the community. The risks we found associated with children's low well-being and physical interactions with non-household family members are likely to exacerbate existing social and educational inequalities.

Understanding such factors are important if policy makers and schools are to provide effective measures that may mitigate these risks in future school closures.

Given the importance of schooling for a child's health we believe that this manuscript is appropriate for publication by PLOS ONE and will be of interest to your readers.

We have disclosed that G.J.R., R.A. and L.S. participate in the UK's Scientific Advisory Group for Emergencies, or its subgroups. These groups did not fund the study or authors. RA is an employee of the UK Health Security Agency. The sponsors had no role in the design, execution, interpretation, or writing of the study. The corresponding author had full access to all the data and had final responsibility for the decision to submit for publication.

Please address all correspondence concerning this manuscript to me at lisa.woodland@kcl.ac.uk.

Thank you for your consideration of this manuscript.

Sincerely,

Lisa Woodland (on behalf of all authors)

Manuscript

Click here to access/download;Manuscript;Manuscript 12.01.23.docx

Children's interactions with non-household family members and child well-being during the covid-19 pandemic: a cross-sectional survey

Short title: Children's interactions with non-household family members and their well-being during COVID-19

Lisa Woodland[1,5*],

Louise E. Smith[1,5],

Samantha K. Brooks[1,5],

Rebecca K. Webster[2],

Richard Amlôt[3,5],

Antonia Rubin[4],

G James Rubin[1,5]

[1] Department of Psychological Medicine, King's College London, London, United Kingdom

[2] Department of Psychology, University of Sheffield, Sheffield, United Kingdom

[3] Behavioural Science and Insights Unit, UK Health Security Agency, Salisbury, United Kingdom

[4] Trustee at Weald of Kent Grammar School, Tonbridge, Kent, United Kingdom

[5] NIHR Health Protection Research Unit in Emergency Preparedness and Response

* Corresponding author

E-mail: lisa.woodland@kcl.ac.uk

ABSTRACT

In England (UK), at the start of the COVID-19 pandemic, schools were closed, and the public were required to reduce social interactions to slow the spread of COVID-19. We investigated the risk factors for children having, 1) close contact with family members from outside the household (non-adherent behaviour) and 2) low well-being (Revised Child Anxiety and Depression Scale). We conducted a cross-sectional survey between 8 and 11 June 2020 in parents (n = 2,010) who were aged eighteen years or over and had a school-aged child (4-18 years old). Parents reported that 15% (n = 309) of children had contact with a family member they did not live with and that 26% (n = 519) had low well-being. Children were more likely to have non-household family interactions when they had special educational needs, had a lower well-being, were vulnerable to COVID-19, lived with someone who was over 70 years of age and their parent had low well-being. Children were more likely to have a lower well-being when they had special educational needs, were vulnerable to COVID-19, lived with someone else who was also vulnerable to COVID-19, or lived with someone that was over 70 years old.

1 INTRODUCTION

On 23 March 2020, England went into “lockdown,” with the public required to stay at home to slow the spread of COVID-19 [1]. The public were only allowed to leave home for a limited number of reasons, which included for medical attention, to buy necessities (as infrequently as possible), for exercise (once a day) and to work if absolutely necessary and were unable to work from home. Physical distancing guidelines were implemented: people from different households were not allowed to

meet and individuals were asked to stay two meters away from people not in their household. Non-essential shops were closed, and schools were closed to most children. Children could attend school if they were on a ‘health care plan’ due to a specific medical or social need or if their parent(s) were employed in jobs that were essential to the COVID-19 response (‘key workers’), such as doctors, teachers, and supermarket employees [2]. After two months of lockdown guidance, the restrictions started to ease (see Box One).

On 11 May it was announced that people who could not work from home were able to return to work, the restrictions on spending time outside were lifted, and individuals were allowed to arrange meetings with one person from another household as long as this happened outside and a two meters distance was maintained [3].
From 1 June 2020, the rules relaxed further: six people from different households were able to meet outside (though still at a two meter distance) and more children were eligible to attend school, children in reception (four to five years), year one (five to six years) and year six (10 to 11 years) [4].

Box 1 COVID-19 lockdown restrictions came into effect on 23 March 2020, in England. The restrictions that had been eased since the COVID-19 lockdown restrictions that started 23 March 2020.

While all of the restrictions caused strain within society, the closure of schools was particularly problematic and had adverse impacts on children [5]. Determining, at the height of a crisis, whether the epidemiological benefits of school closures in terms of reducing disease transmission will exceed the psychological, physical, educational, and social costs is a daunting challenge for those who must make this decision. One question that must be factored into decision making is ‘where do children go when their school is shut?’. In an ideal world (from an epidemiological perspective), parents would be able to ensure that children are placed under appropriate alternative supervision and remain apart from each other: continued mixing between children and other households would defeat the purpose of school

closures. A systematic review of unplanned school closures prior to the COVID-19 pandemic found a 65% reduction in the mean total number of contacts for each student whilst schools were closed [6]. However, all 19 studies included in the review reported that children continued to meet with people from other households to some extent. A common reason for meeting others related to childcare. For example, one study found that children in households where special childcare arrangements were needed during the closure were significantly more likely to leave home than children who were more independent and able to take care of themselves [7]. Parental concerns about the school closure also commonly reflected the difficulties they would face in trying to arrange childcare as well as concerns about lost education [6].

In the context of the COVID-19 pandemic, issues around childcare while schools were closed were of particular concern because of a worry that grandparents might be called on to look after children in some families, putting older adults at increased risk of infection [8]. It was not just policy makers who worried about this, many children were themselves worried about their grandparents' vulnerability to COVID-19 [9] and of the risk that they themselves might infect their grandparents [10]. The extent to which these worries were borne out remains unclear.

Of course, while keeping children separate from each other and away from their grandparents might reduce disease transmission, it does present other risks. For example, a reduction in physical activity levels as a result of staying at home can affect physical and mental well-being [5]. While parents were advised to ensure their children engaged in physical activity during the initial lockdown, the extent to which this occurred, and the impact of that initial period of isolation and inactivity is uncertain. Some parents and children may also have been very cautious about the

risks associated with COVID-19, engaging in additional protective behaviours that were not explicitly recommended by the Government and which may have exerted an additional toll on well-being [11, 12].

In this study we investigated the risk factors associated with parents reporting children having 1) close contact with family members from outside their household and 2) low well-being during the first COVID-19 lockdown in the UK. We specifically explored whether close contact with non-household family members and low child well-being were associated with: parents' age, gender, and employment characteristics, children's age, gender and special educational needs status, and household's vulnerability to, perceptions about and behaviours in relation to COVID-19.

2 MATERIALS AND METHODS

2.1 Design

We commissioned a market research company, BMG Research, to administer a cross-sectional survey between 8 and 11 June 2020 [13]. Data collection occurred after lockdown restrictions had begun to be eased. At this stage in the pandemic, schools had re-opened to children in reception (4 to 5 years), year one (5 to 6 years) and year six (ten to eleven years). However, physical distancing restrictions remained in place throughout society, and while up to six non-household members could meet, this had to be outside and at a two-meter distance.

We have previously published data from this survey relating to parental perceptions of the hygiene procedures within schools [14] and investigating why some parents did not send their eligible children back to school [15].

2.2 Participants

Participants (n=2,447) were recruited from BMG Research’s panel. To achieve a sample that was broadly representative of the population, BMG Research monitored region, child age, child gender, parent/guardian age, and parent/guardian gender. Participants were eligible for the study if they were aged eighteen years or over, lived in England, and were a parent or guardian to a school-aged child (4-18 years old) who usually lived with them. 183 participants were screened out for non-eligibility, 226 participants dropped out after starting the survey and 28 completed but were removed for reasons related to quality control, such as completing the survey quickly or for ‘straight-lining’ (selecting the same option for every question) suggesting inattention to the questions. 2,010 participants remained.

The sample fell within five percentage points of the national population by the child’s gender, key stage, and type of school attended against the known distribution for school children in England [16].

Participants were paid in points equivalent to £0.[illegible].

The research was approved by the Psychiatry, Nursing and Midwifery Research Ethics Subcommittee at King’s College London (LRS-19/20-18787).

2.3 Study Materials

The full survey is available in the supplementary materials (S.1 Text).

All participants answered questions referring to their child who had the most recent birthday. In rare cases where children shared a birthday, we asked the parent to select one child.

2.3.1 Parent and child personal characteristics

We asked parents to report their gender, age, region, household income, employment status, and if employed, whether they were working from home, level of education, marital status, ethnicity, and key worker status. We asked parents to report the child's gender, age, school year, and whether the child had special educational needs.

We also asked whether anyone within the household was aged over 70 years old or had a health condition that made them vulnerable to COVID-19 and whether they had access to outside space.

2.3.2 Child's physical interactions with non-household members

We asked parents to choose from a list of seven options about people who the child had come into close contact with in the past 24 hours ("someone [child] lives with;" "friends or other children who [child] does not live with"; "a babysitter, nanny or childminder;" "family member aged under 70 who [child] does not live with;" "family member aged over 70 who [child] does not live with;" "other children, not already reported above"; and "other adults, not already reported above"). We made clear in the question that "by close contact we mean closer than 2 meters, for fifteen minutes or more." Parents were asked to report all the options that applied.

2.3.3 Child's well-being

We asked parents to report the child's well-being using two subscales from the Revised Child Anxiety and Depression Scale (RCADS): the generalised anxiety disorder (GAD) sub-scale and the major depressive disorder (MDD) sub-scale [17]. The GAD sub-scale asks parents to respond to six statements about their child (e.g., "my child worries about things"; and "my child worries that something awful will happen to someone in the family"). The MDD sub-scale asks parents to respond to

ten statements about their child (e.g., “my child feels sad or empty”; “nothing is much fun for my child”; and “my child has trouble sleeping”). Parents can respond “never,” “sometimes,” “often,” and “always.”

2.3.4 Parent’s well-being

We asked parents to report their well-being using the Patient-Health Questionnaire-4 (PHQ4) [18], which asks “over the last two weeks, how often have you been bothered by the following problems:” “feeling nervous, anxious or on the edge;” “not being able to stop or control worrying;” “little interest or pleasure in doing things;” and “feeling down, depressed, or hopeless.” Parents can respond “not at all,” “several days,” “more than half the days,” and “nearly every day.”

2.3.5 Child’s activities outside the home

We asked parents how many times the child had left the home in the past seven days: “to go to the shops for groceries, toiletries, or medicines;” “to go to the shops for other items;” “for exercise;” “for a medical need (e.g., an outpatient appointment);” “to go to school;” “to provide help to someone else;” “to meet friends;” to meet family members who they did not live with ; and “for another reason”.

2.3.6 Statements about lockdown

We included 16 statements about lockdown, which included questions about COVID-19 (e.g., “if [child] goes out, she/he is likely to catch coronavirus”), schooling (e.g., “[child] is keeping up with his/her schoolwork”) and home environment (e.g., “in the past 7 days, [child] has been bored”). Parents responded to each statement using a five-point Likert-scale from “strongly agree” to “strongly disagree,” or “not applicable.”

2.4 Patient and public involvement

A school trustee contributed to the development of the survey materials and co-authored this paper. The survey questions were reviewed by five parents of school children who also piloted the survey before publication. The feedback we received resulted in minor changes to the wording of some survey questions for clarity.

2.5 Analysis: recoding variables

2.5.1 Outcome one: children's physical interactions with non-household family members

We created a binary variable to indicate whether the child had close contact with a family member from outside their household, defined as a "non-household interaction." This included children reported as having had close contact (closer than 2 meters, for fifteen minutes or more) with either "a family member aged under 70 who [child] does not live with" or "a family member aged over 70 who [child] does not live with."

2.5.2 Outcome two: children with a low well-being

We created a binary variable to indicate well-being in the child. The variable was recoded using SPSS syntax supplied by the RCADS authors, which assigns a value against each answer from 0 ("never") to 3 ("always") on the GAD and MDD RCADS sub-scales and creates a total score for each sub-scale [17]. The total score is turned into a t-score, normalising the RCADS scores within the population, by child's age and gender. A t-score of 65 (approximately in the top 7% of un-referred young people of the same age) on either the GAD or MDD sub-scale was our well-being cut off. "Lower well-being" represents a child with a medium or severe risk of clinical mental illness and "higher well-being" represents a child with low risk of clinical mental illness.

2.5.3 Predictor variables

We recoded household income, employment status, parent education level, marital status, and ethnicity into binary variables. We recoded parent age, key worker status, and access to outside space into categorical variables, as shown in the results tables. We recoded child school year into Key Stages as used in the English education system (Reception = ages 4 to 5; Key Stage 1 = ages 5 to 7; Key Stage 2 = ages 7 to 11; Key Stage 3 = ages 11 to 14; Key Stage 4 = ages 14 to 16; Years 12 and 13 = ages 16 to 18).

We created a binary variable to indicate low well-being in the parent. We assigned a value against each answer from 0 (“not at all”) to 3 (“nearly every day”) on the PHQ4. We used a cut off score of 5 or above to indicate low well-being in the parent, indicating moderate or severe risk of clinical anxiety or depression.

We created two binary variables to indicate whether the child, and someone in the household (other than the child) had a health condition that might make them particularly vulnerable to COVID-19. The responses “yourself [participant]” and “anyone else you live with” were combined into one variable to indicate that the child lived with someone vulnerable to COVID-19.

We created a continuous variable to indicate the COVID-19 behaviours that parents and their child had followed by combining all 11 responses to the statements about what the parent or child had done in the past seven days because of the risk of COVID-19.

Items about lockdown and the child’s activities outside the home were used as continuous variables.

For all variables, we coded the responses “don’t know”, “not applicable”, “prefer not to say” and “prefer to self-describe” as missing data.

2.6 Analysis

We ran a series of binary logistic regressions using SPSS [19, 20] investigating the univariable associations between our two outcomes and each of our predictor variables. We ran a second set of binary logistic regressions controlling for child and parent characteristics (participant gender, age, region, household income, employment status, education level, marital status, ethnicity, and the child’s gender and school year).

We applied a Bonferroni correction to our results ($p \leq 0.001$) due to running many analyses. Only associations that met this level are discussed narratively in the results section.

3 RESULTS

3.1 Risk factors associated with children’s non-household family interactions

Of the 2010 parent responses, 15% (95% CI 14% to 17%, n=309) of children had had close contact with a family member that they did not live with in the past 24 hours. This included 9% (n=189) who had interactions with a family member aged under 70 years and 6% (n=120) who had interactions with a family member aged 70 years or older, which includes n = 45 who had interactions with a non-household family member under and over 70 years of age. The parent and child characteristics associated with children who had non-household family interactions are shown in Table 1. Children were more likely to have non-household family interactions when their parent had low well-being. Children were also more likely to have had non-

household family interactions when they had special educational needs, had a lower well-being, were vulnerable to COVID-19, and lived with someone who was over 70 years of age.

Table 2 shows the associations between children who had non-household family interactions and the predictor variables relating to parent perceptions about lockdown. Non-household family interaction was associated with parents' agreement that their child had extra support at school before the closures, were upset about not seeing other family members that they did not live with, and that the parent had found it hard to keep up with work or other important commitments.

The behaviours that families followed because of the risk of COVID-19 are presented in Table 6 (see S2 Appendix A).

Table 1 Binary logistic regression comparing parent and child characteristics and associations with children's non-household family interact[illegible]s.

Variable	Level	Child's not had family interactions, n, (%)	Child's had family interactions, n, (%)	Odds Ratio (95% CI)	P-value	Adjusted odds ratio (95% CI) †	P-value
Gender	Male	797 (86)	134 (14)	0.88 (0.69 to 1.12)	0.30	0.97 (0.74 to 1.27)	0.80
	Female	894 (84)	171 (16)	Reference		Reference	
Age	18-35 years	305 (77)	89 (23)	**2.13** (1.54 to 2.95)**	**<0.001**	**1.28* (1.25 to 2.65)**	**0.002**
	36-45 years	738 (85)	130 (15)	1.29 (0.97 to 1.72)	0.09	1.17 (0.85 to 1.62)	0.34
	46 years ≥	658 (88)	90 (12)	Reference		Reference	
Region	East Midlands	129 (85)	22 (15)	0.73 (0.43 to 1.24)	0.25	0.69 (0.40 to 1.21)	0.19
	East of England	186 (85)	33 (15)	0.76 (0.48 to 1.21)	0.25	0.77 (0.47 to 1.26)	0.30
	North East	104 (88)	14 (12)	0.58 (0.31 to 1.08)	0.08	0.55 (0.28 to 1.06)	0.07
	North West	232 (85)	41 (15)	0.76 (0.50 to 1.17)	0.21	0.83 (0.52 to 1.32)	0.43
	South East	283 (84)	52 (16)	0.79 (0.53 to 1.18)	0.25	0.84 (0.55 to 1.28)	0.43
	South West	143 (88)	20 (12)	0.60 (0.35 to 1.03)	0.07	0.61 (0.35 to 1.08)	0.09
	West Midlands	183 (87)	28 (13)	0.66 (0.41 to 1.07)	0.09	0.63 (0.37 to 1.05)	0.08
	Yorkshire & the Humber	161 (83)	34 (17)	0.91 (0.58 to 1.44)	0.69	0.88 (0.54 to 1.44)	0.61
	London	280 (81)	65 (19)	Reference		Reference	
Household income	≤ £34,999	660 (82)	144 (18)	**1.34* (1.05 to 1.72)**	**0.02**	**1.44* (1.08 to 1.92)**	**0.01**
	£35,000 ≥	958 (86)	156 (14)	Reference		Reference	
Employment status[1]	Working	1412 (84)	265 (16)	0.27 (1.21 to 0.86 to 1.72)	0.27	1.12 (0.77 to 1.63)	0.57
	Not working	278 (87)	43 (13)	Reference		Reference	
Parent Working from home[2]	Yes	774 (82)	166 (18)	**1.53* (1.15 to 2.05)**	**0.004**	^^^	^^^
	No	551 (88)	77 (12)	Reference		Reference	
Education level	≤ A-level	740 (87)	115 (13)	**0.77* (0.60 to 0.98)**	**0.04**	**0.70* (0.53 to 0.93)**	**0.01**
	Degree ≥	947 (83)	192 (17)	Reference		Reference	
Marital status	Living alone	262 (80)	65 (20)	**1.46* (1.08 to 1.98)**	**0.01**	1.36 (0.98 to 1.90)	0.07
	Married/cohabiting	1439 (86)	244 (14)	Reference		Reference	
Ethnicity	White	1483 (85)	270 (15)	0.97 (0.67 to 1.41)	0.88	1.25 (0.83 to 1.87)	0.28
	BAME	203 (84)	38 (16)	Reference		Reference	
Key worker status	Both parents	167 (86)	28 (14)	1.18 (0.76 to 1.84)	0.47	1.18 (0.74 to 1.90)	0.49
	One parent	686 (81)	161 (19)	**1.65** (1.28 to 2.14)**	**<0.001**	**1.53* (1.16 to 2.02)**	**0.002**
	No	837 (88)	119 (12)	Reference		Reference	
Child gender	Boy	891 (84)	172 (16)	1.14 (0.89 to 1.46)	0.29	1.14 (0.88 to 1.48)	1.14
	Girl	810 (86)	137 (14)	Reference		Reference	
School year	Early Years	85 (84)	16 (16)	1.53 (0.73 to 3.18)	0.26	1.22 (0.55 to 2.71)	0.63
	Key Stage 1	279 (78)	77 (22)	**2.24* (1.28 to 3.94)**	**0.01**	**2.11* (1.13 to 3.94)**	**0.02**

	Key Stage 2	576 (85)	99 (15)	1.40 (0.81 to 2.41)	0.23	1.28 (0.70 to 2.33)	0.42
	Key Stage 3	334 (85)	61 (15)	1.48 (0.84 to 2.63)	0.18	1.53 (0.83 to 2.83)	0.17
	Key Stage 4	289 (88)	39 (12)	1.10 (0.60 to 2.01)	0.77	1.21 (0.64 to 2.30)	0.56
	Years 12 & 13	138 (89)	17 (11)	Reference		Reference	
Child had special educational needs	Yes	118 (73)	43 (27)	**2.16** (1.49 to 3.14)**	**<0.001**	**2.19** (1.47 to 3.27)**	**<0.001**
	No	1568 (86)	264 (14)	Reference		Reference	
Child lower well-being	Yes	384 (74)	135 (26)	**2.66** (2.07 to 3.42)**	**<0.001**	**2.65** (2.03 to 3.46)**	**<0.001**
	No	1317 (88)	174 (12)	Reference		Reference	
Parent lower well-being	Yes	295 (75)	96 (25)	**2.15** (1.64 to 2.82)**	**<0.001**	**1.94** (1.45 to 2.58)**	**<0.001**
	No	1406 (87)	213 (13)	Reference		Reference	
Child vulnerable COVID-19	Yes	111 (71)	46 (29)	**2.50** (1.73 to 3.61)**	**<0.001**	**2.17** (1.45 to 3.25)**	**<0.001**
	No	1566 (86)	260 (14)	Reference		Reference	
Household vulnerable COVID-19	Yes	429 (84)	81 (16)	1.16 (0.87 to 1.54)	0.32	1.17 (0.87 to 1.59)	0.30
	No	1127 (86)	184 (14)	Reference		Reference	
Someone over 70 years	Yes	59 (68)	28 (32)	**2.77** (1.74 to 4.42)**	**<0.001**	**2.56** (1.55 to 4.24)**	**<0.001**
	No	1642 (85)	281 (15)	Reference		Reference	
COVID-19 behaviours followed by child and parent	Continuous (0=followed no behaviours, 11=followed all behaviours)	N=1701, M=5.51, SD=3.06	N=309, M=6.11, SD=3.27	**1.06* (1.02 to 1.11)**	**0.002**	1.04 (0.99 to 1.08)	0.10
Access to outside space[3]	Garden	1524 (85)	260 (15)	0.73 (0.40 to 1.38)	0.33	0.88 (0.44 to 1.75)	0.71
	Other[3]	121 (77)	36 (23)	1.28 (0.63 to 2.60)	0.49	1.25 (0.58 to 2.73)	0.71
	No	56 (81)	13 (19)	Reference		Reference	

* $p \leq \cdot05$ and formatted bold
** $p \leq \cdot001$ and formatted bold
Some results are rounded to 3 rather than 2 decimal places to distinguish between $p \leq \cdot05$ and $p \leq \cdot001$
† Adjusting for participant gender, age, region, household income, employment status, education level, marital status, ethnicity, and the child's gender and school year
^^^ Not included variable highly correlated with employment variable
[1] Working includes students and volunteers
[2] Question only offered to participants who reported working in a paid job (full-time, part-time, and self-employed) and not to participants who reported being a student, on furlough and a volunteer.
[3] Participants that reported no access to a garden but had access to other outdoor spaces such as patio, terrace, and balcony

Table 2 Binary logistic regression comparing statements about lockdown and associations with children's non-household family interactions. Family interaction is defined by a child's close contact with a family member outside the household (n=309)

Statement	Level	Child's not had family interactions, n, (%)	Child's had family interactions, n, (%)	Odds ratio (95%, CI)	P-value	Adjusted odds ratio (95%, CI) †	P-value
If child goes out, she/he is likely to catch coronavirus	5-point Likert-scale (1=strongly agree, 5=strongly disagree)	N=1672, M=3.27, SD=1.03	N=309, M=3.08, SD=1.18	**0.85* (0.75 to 0.95)**	**0.004**	0.89 (0.79 to 1.01)	0.07
If child goes out, she/he is likely to bring coronavirus back into our home	5-point Likert-scale (1=strongly agree, 5=strongly disagree)	N=1672, M=3.23, SD=1.07	N=309, M=3.05, SD=1.16	**0.86* (0.77 to 0.96)**	**0.01**	0.91 (0.81 to 1.03)	0.13
Child is keeping up with his/her schoolwork	5-point Likert-scale (1=strongly agree, 5=strongly disagree)	N=1672, M=2.12, SD=1.08	N=309, M=2.30, SD=1.15	**1.16* (1.04 to 1.29)**	**0.01**	**1.14* (1.02 to 1.27)**	**0.03**
I feel confident helping child with her/his schoolwork	5-point Likert-scale (1=strongly agree, 5=strongly disagree)	N=1672, M=2.23, SD=1.03	N=305, M=2.30, SD=1.07	1.06 (0.94 to 1.19)	0.32	1.08 (0.96 to 1.23)	0.20
I feel supported by child's school	5-point Likert-scale (1=strongly agree, 5=strongly disagree)	N=1681, M=2.32, SD=1.07	N=306, M=2.32, SD=1.07	1.00 (0.89 to 1.12)	0.96	0.99 (0.68 to 1.12)	0.87
I have access to all the resources that child needs to do her/his schoolwork	5-point Likert-scale (1=strongly agree, 5=strongly disagree)	N=1678, M=2.15, SD=1.02	N=307, M=2.21, SD=1.03	1.05 (0.94 to 1.18)	0.38	1.09 (0.96 to 1.23)	0.18
During lockdown, child has learned about important things she/he wouldn't normally learn at school	5-point Likert-scale (1=strongly agree, 5=strongly disagree)	N=1691, M=2.40, SD=1.00	N=308, M=2.38, SD=1.05	0.98 (0.87 to 1.11)	0.74	1.03 (0.30 to 1.17)	0.69
In the past 7 days, child has been bored	5-point Likert-scale (1=strongly agree, 5=strongly disagree)	N=1694, M=2.54, SD=1.20	N=309, M=2.30, SD=1.16	**0.84** (0.75 to 0.93)**	**0.001**	**0.85* (0.76 to 0.95)**	**0.004**
In the past 7 days, my household has had a regular structure to the day	5-point Likert-scale (1=strongly agree, 5=strongly disagree)	N=1692, M=2.31, SD=1.02	N=309, M=2.43, SD=1.12	1.12 (1.00 to 1.26)	0.05	1.09 (0.96 to 1.23)	0.18
In the past 7 days, child has kept in touch with her/his friends	5-point Likert-scale (1=strongly agree, 5=strongly disagree)	N=1691, M=2.25, SD=1.14	N=307, M=2.26, SD=1.12	1.00 (0.90 to 1.12)	0.96	0.95 (0.84 to 1.07)	0.36
Child is worried about coronavirus	5-point Likert-scale (1=strongly agree, 5=strongly disagree)	N=1690, M=2.72, SD=1.12	N=306, M=2.70, SD=1.23	0.98 (0.88 to 1.09)	0.72	0.99 (0.88 to 1.10)	0.82

In the past 7 days, child has felt upset about not seeing other family members who do not live with us	5-point Likert-scale (1=strongly agree, 5=strongly disagree)	N=1684, M=2.97, SD=1.24	N=308, M=2.65, SD=1.25	**0.81** (0.73 to 0.89)**	**<0.001**	**0.84** (0.75 to 0.93)**	**0.001**
In the past 7 days, I have found it hard to keep up with work or other important commitments	5-point Likert-scale (1=strongly agree, 5=strongly disagree)	N=1626, M=3.22, SD=1.21	N=299, M=2.79, SD=1.21	**0.75** (0.67 to 0.83)**	**<0.001**	**0.78** (0.70 to 0.87)**	**<0.001**
In the past 7 days, people in my household have been getting along well	5-point Likert-scale (1=strongly agree, 5=strongly disagree)	N=1691, M=2.08, SD=0.95	N=309, M=2.19, SD=1.01	1.13 (1.00 to 1.28)	0.05	1.07 (0.94 to 1.22)	0.28
I am worried about the financial impact of lockdown measures	5-point Likert-scale (1=strongly agree, 5=strongly disagree)	N=1685, M=2.51, SD=1.21	N=304, M=2.40, SD=1.16	0.92 (0.83 to 1.02)	0.13	0.93 (0.83 to 1.03)	0.18
Before the school closures, child had extra support at school	5-point Likert-scale (1=strongly agree, 5=strongly disagree)	N=1528, M=3.59, SD=1.36	N=285, M=3.16, SD=1.48	**0.80** (0.73 to 0.88)**	**<0.001**	**0.82** (0.74 to 0.90)**	**<0.001**

* $p \leq \cdot 05$ and formatted bold
** $p \leq \cdot 001$ and formatted bold
Some results are rounded to 3 rather than 2 decimal places to distinguish between $p \leq \cdot 05$ and $p \leq \cdot 001$
† Adjusting for participant gender, age, region, household income, employment status, education level, marital status, ethnicity, and the child's gender and school year

3.2 Risk factors associated with children's lower well-being

Of the 2010 parent responses, 26% (95% CI 24% to 28%, n=519) reported that their child had a low well-being. The parent and child characteristics associated with child low well-being are shown in Table 3. Children were more likely to have a lower well-being when the parent was aged between 18 and 35 years old, when one of their parents were a key worker and when the parent had lower well-being. Children were also more likely to have a lower well-being when they had special educational needs, were vulnerable to COVID-19, lived with someone else who was also vulnerable to COVID-19 and lived with someone that was over 70 years old. Children were more likely to have a lower well-being if their household followed multiple precautionary behaviours because of the risk of COVID-19. All lockdown statements bar two were associated with lower well-being (Table 4).

Table 3 Binary logistic regression comparing parent and child characteristics and associations with children's lower well-being (n=519)

Variable	Level	Child higher well-being, n, (%)	Child lower well-being, n, (%)	Odds Ratio (95% CI)	P-value	Adjusted odds ratio (95% CI) †	P-value
Gender	Male	702 (75)	229 (25)	0.91 (0.74 to 1.11)	0.36	0.92 (0.74 to 1.15)	0.46
	Female	784 (74)	281 (26)	Reference		Reference	
Age	18-35 years	263 (67)	131 (33)	**1.67** (1.27 to 2.19)**	**<0.001**	**1.92** (1.40 to 2.64)**	**<0.001**
	36-45 years	652 (75)	216 (25)	1.11 (0.88 to 1.40)	0.38	1.25 (0.96 to 1.62)	0.09
	46 years ≥	576 (77)	172 (23)	Reference		Reference	
Region	East Midlands	101 (67)	50 (33)	1.27 (0.84 to 1.91)	0.26	1.21 (0.78 to 1.88)	0.39
	East of England	163 (74)	56 (26)	0.88 (0.60 to 1.29)	0.51	0.93 (0.61 to 1.39)	0.71
	North East	92 (78)	26 (22)	0.72 (0.44 to 1.18)	0.20	0.77 (0.46 to 1.30)	0.33
	North West	208 (76)	65 (24)	0.80 (0.56 to 1.15)	0.23	0.90 (0.61 to 1.33)	0.61
	South East	248 (74)	87 (26)	0.90 (0.64 to 1.26)	0.53	0.90 (0.62 to 1.29)	0.56
	South West	118 (72)	45 (28)	0.98 (0.64 to 1.48)	0.91	1.04 (0.67 to 1.63)	0.85
	West Midlands	161 (76)	50 (24)	0.79 (0.54 to 1.18)	0.25	0.75 (0.49 to 1.15)	0.19
	Yorkshire & the Humber	152 (78)	43 (22)	0.72 (0.48 to 1.09)	0.12	0.71 (0.46 to 1.11)	0.13
	London	248 (72)	97 (28)	Reference		Reference	
Household income	≤ £34,999	583 (73)	221 (27)	1.12 (0.91 to 1.37)	0.29	1.08 (0.85 to 1.37)	0.54
	£35,000 ≥	832 (75)	282 (25)	Reference		Reference	
Employment status[1]	Working	1240 (74)	437 (26)	1.12 (0.85 to 1.48)	0.44	1.12 (0.82 to 1.52)	0.48
	Not working	244 (76)	77 (24)	Reference		Reference	
Parent Working from home[2]	Yes	682 (73)	258 (27)	1.16 (0.92 to 1.47)	0.20	^^^	^^^
	No	474 (75)	154 (25)	Reference		Reference	
Education level	≤ A-level	642 (75)	213 (25)	0.92 (0.75 to 1.12)	0.39	0.92 (0.74 to 1.16)	0.50
	Degree ≥	836 (73)	303 (27)	Reference		Reference	
Marital status	Living alone	234 (72)	93 (28)	1.17 (0.90 to 1.53)	0.24	1.15 (0.87 to 1.53)	0.33
	Married/cohabiting	1257 (75)	426 (25)	Reference		Reference	
Ethnicity	White	1304 (74)	449 (26)	0.93 (0.69 to 1.26)	0.65	1.06 (0.76 to 1.48)	0.73
	BAME	176 (73)	65 (27)	Reference		Reference	
Key worker status	Both parents	134 (69)	61 (31)	**1.70* (1.21 to 2.39)**	**0.002**	**1.71* (1.18 to 2.46)**	**0.004**
	One parent	593 (70)	254 (30)	**1.60** (1.29 to 1.98)**	**<0.001**	**1.51** (1.20 to 1.90)**	**<0.001**
	No	754 (79)	202 (21)	Reference		Reference	
Child gender	Boy	771 (73)	292 (27)	1.20 (0.98 to 1.47)	0.07	1.22 (0.98 to 1.50)	0.07
	Girl	720 (76)	227 (24)	Reference		Reference	
School year	Early Years	77 (76)	24 (24)	0.99 (0.55 to 1.79)	0.98	0.71 (0.38 to 1.32)	0.28
	Key Stage 1	263 (74)	93 (26)	1.13 (0.73 to 1.75)	0.59	0.80 (0.50 to 1.29)	0.37
	Key Stage 2	517 (7)	158 (23)	0.97 (0.65 to 1.47)	0.90	0.71 (0.46 to 1.10)	0.13
	Key Stage 3	286 (72)	109 (28)	1.22 (0.79 to 1.87)	0.37	1.03 (0.66 to 1.61)	1.61

	Key Stage 4	230 (70)	98 (30)	1.36 (0.88 to 2.11)	0.17	1.15 (0.73 to 1.80)	0.55
	Years 12 & 13	118 (76)	37 (24)	Reference		Reference	
Child has special educational needs	Yes	71 (44)	90 (56)	**4.24** (3.05 to 5.89)**	**<0.001**	**4.13** (2.90 to 5.87)**	**<0.001**
	No	1410 (770	422 (23)	Reference		Reference	
Parent low well-being	Yes	159 (41)	232 (59)	**6.77** (5.33 to 8.60)**	**<0.001**	**7.26** (5.62 to 9.38)**	**<0.001**
	No	1332 (82)	287 (18)	Reference		Reference	
Child vulnerable COVID-19	Yes	77 (49)	80 (51)	**3.34** (2.40 to 4.65)**	**<0.001**	**3.06** (2.15 to 4.36)**	**<0.001**
	No	1393 (76)	433 (24)	Reference		Reference	
Household vulnerable COVID-19	Yes	329 (65)	181 (35)	**2.01** (1.60 to 2.51)**	**<0.001**	**2.08** (1.64 to 2.64)**	**<0.001**
	No	1029 (78)	282 (22)	Reference		Reference	
Someone over 70 years	Yes	46 (53)	41 (47)	**2.69** (1.75 to 4.16)**	**<0.001**	**2.41** (1.51 to 3.83)**	**<0.001**
	No	1445 (75)	478 (25)	Reference		Reference	
COVID-19 behaviours followed by child and parent	Continuous (0=followed no behaviours, 11=followed all behaviours)	N=1491, M=5.35, SD=3.02	N=519, M=6.32, SD=3.22	**1.11** (1.07 to 1.14)**	**<0.001**	**1.11** (1.07 to 1.15)**	**<0.001**
Access to outside space[3]	Garden	1333 (75)	451 (25)	0.89 (0.52 to 1.53)	0.67	1.10 (0.60 to 2.03)	0.76
	Other[3]	108 (69)	49 (31)	1.19 (0.64 to 2.23)	0.58	1.36 (0.68 to 2.73)	0.38
	No	50 (72)	19 (28)	Reference		Reference	

* *p≤ ·05* and formatted bold

** *p≤ ·001* and formatted bold

Some results are rounded to 3 rather than 2 decimal places to distinguish between *p≤ ·05* and *p≤ ·001*

† Adjusting for participant gender, age, region, household income, employment status, education level, marital status, ethnicity, and the child's gender and school year

^^^ Not included variable highly correlated with employment variable

[1] Working includes students and volunteers

[2] Question only offered to participants who reported working in a paid job (full-time, part-time, and self-employed) and not to participants who reported being a student, on furlough and a volunteer.

[3] Participants that reported no access to a garden but had access to other outdoor spaces such as patio, terrace, and balcony

Table 4 Binary logistic regression comparing statements about lockdown and associations with children's lower well-being (n=519)

Statement	Level	Child higher well-being, n, (%)	Child lower well-being, n, (%)	Odds ratio (95%, CI)	P-value	Adjusted odds ratio (95%, CI) †	P-value
If child goes out, she/he is likely to catch coronavirus	5-point Likert-scale (1=strongly agree, 5=strongly disagree)	N=1473, M=3.37, SD=1.02	N=515, M=2.87, SD=1.06	**0.63** (0.57 to 0.70)**	**<0.001**	**0.63** (0.57 to 0.70)**	**<0.001**
If child goes out, she/he is likely to bring coronavirus back into our home	5-point Likert-scale (1=strongly agree, 5=strongly disagree)	N=1465, M=3.33, SD=1.04	N=515, M=2.82 SD=1.11	**0.63** (0.58 to 0.70)**	**<0.001**	**0.63** (0.57 to 0.70)**	**<0.001**
Child is keeping up with his/her schoolwork	5-point Likert-scale (1=strongly agree, 5=strongly disagree)	N=1470, M=2.03, SD=1.01	N=508, M=2.48, SD=1.24	**1.43** (1.31 to 1.57)**	**<0.001**	**1.46** (1.33 to 1.60)**	**<0.001**
I feel confident helping child with her/his schoolwork	5-point Likert-scale (1=strongly agree, 5=strongly disagree)	N=1469, M=2.16, SD=0.98	N=508, M=2.48, SD=1.13	**1.34** (1.22 to 1.48)**	**<0.001**	**1.34** (1.21 to 1.48)**	**<0.001**
I feel supported by child's school	5-point Likert-scale (1=strongly agree, 5=strongly disagree)	N=1475, M=2.25, SD=1.03	N=512, M=2.52 SD=1.15	**1.26** (1.15 to 1.38)**	**<0.001**	**1.24** (1.12 to 1.37)**	**<0.001**
I have access to all the resources that child needs to do her/his schoolwork	5-point Likert-scale (1=strongly agree, 5=strongly disagree)	N=1473, M=2.09, SD=0.97	N=512, M=2.37, SD=1.14	**1.30** (1.18 to 1.43)**	**<0.001**	**1.31** (1.18 to 1.44)**	**<0.001**
During lockdown, child has learned about important things she/he wouldn't normally learn at school	5-point Likert-scale (1=strongly agree, 5=strongly disagree)	N=1481, M=2.37, SD=0.98	N=518, M=2.49, SD=1.08	**1.12* (1.01 to 1.24)**	**0.02**	**1.13* (1.02 to 1.25)**	**0.02**
In the past 7 days, child has been bored	5-point Likert-scale (1=strongly agree, 5=strongly disagree)	N=1486, M=2.63, SD=1.20	N=517, M=2.14, SD=1.11	**0.68** (0.62 to 0.75)**	**<0.001**	**0.68** (0.61 to 0.75)**	**<0.001**
In the past 7 days, my household has had a regular structure to the day	5-point Likert-scale (1=strongly agree, 5=strongly disagree)	N=1484, M=2.27, SD=0.99	N=517, M=2.50, SD=1.14	**1.23** (1.12 to 1.36)**	**<0.001**	**1.23** (1.11 to 1.36)**	**<0.001**
In the past 7 days, child has kept in touch with her/his friends	5-point Likert-scale (1=strongly agree, 5=strongly disagree)	N=1483, M=2.21, SD=1.12	N=515, M=2.39, SD=1.19	**1.15* (1.05 to 1.25)**	**0.01**	1.67 (0.89 to 3.15)	0.11
Child is worried about coronavirus	5-point Likert-scale (1=strongly agree, 5=strongly disagree)	N=1481, M=2.88, SD=1.12	N=515, M=2.27, SD=1.08	**0.60** (0.54 to 0.66)**	**<0.001**	**0.59** (0.53 to 0.65)**	**<0.001**
In the past 7 days, child has felt upset about not seeing other family members who do not live with us	5-point Likert-scale (1=strongly agree, 5=strongly disagree)	N=1475, M=3.09, SD=1.24	N=517, M=2.45, SD=1.15	**0.64** (0.59 to 0.70)**	**<0.001**	**0.63** (0.57 to 0.69)**	**<0.001**

In the past 7 days, I have found it hard to keep up with work or other important commitments	5-point Likert-scale (1=strongly agree, 5=strongly disagree)	N=1429, M=3.37, SD=1.19	N=496, M=2.55, SD=1.15	0.56** (0.51 to 0.61)	<0.001	0.55** (0.49 to 0.60)	<0.001
In the past 7 days, people in my household have been getting along well	5-point Likert-scale (1=strongly agree, 5=strongly disagree)	N=1484, M=2.00, SD=0.93	N=516, M=2.37, SD=0.97	1.47** (1.33 to 1.63)	<0.001	1.46** (1.31 to 1.63)	<0.001
I am worried about the financial impact of lockdown measures	5-point Likert-scale (1=strongly agree, 5=strongly disagree)	N=1475, M=2.58, SD=1.23	N=514, M=2.24, SD=1.10	0.78** (0.71 to 0.85)	<0.001	0.77** (0.70 to 0.85)	<0.001
Before the school closures, child had extra support at school	5-point Likert-scale (1=strongly agree, 5=strongly disagree)	N=1326, M=3.72, SD=1.32	N=487, M=2.98, SD=1.42	0.68** (0.63 to 0.74)	<0.001	0.69** (0.63 to 0.75)	<0.001

* *p*≤ *·05* and formatted bold
** *p*≤ *·001* and formatted bold
Some results are rounded to 3 rather than 2 decimal places to distinguish between *p*≤ *·05* and *p*≤ *·001*
† Adjusting for participant gender, age, region, household income, employment status, education level, marital status, ethnicity, and the child's gender and school year

Associations between well-being and leaving home

A child was more likely to have a lower well-being if they left home for shopping, to provide help to someone else, to meet family, for medical treatment or for another reason (Table 5). Lower well-being was also associated with not leaving home for exercise.

Table 5 Binary logistic regression comparing reasons for children leaving the home by children with low well-being (n=519)

Number of times that child had left the home for each reason on a continuous scale starting at 0	Child higher well-being, n, (%)	Child lower well-being, n, (%)	Odds ratio (95%, CI)	P-value	Adjusted odds ratio (95%, CI) †	P-value
To go to the shops for groceries, toiletries, or medicines	N=1491, M=0.53, SD=1.03	N=519, M=0.83, SD=1.24	**1.25** (1.15 to 1.36)**	**<0.001**	**1.24** (1.13 to 1.35)**	**<0.001**
To go to the shops for other items	N=1491, M=0.38, SD=0.91	N=519, M=0.68, SD=1.13	**1.32** (1.20 to 1.45)**	**<0.001**	**1.31** (1.19 to 1.45)**	**<0.001**
To provide help to someone else	N=1491, M=0.15, SD=0.71	N=519, M=0.42, SD=0.99	**1.44** (1.27 to 1.64)**	**<0.001**	**1.37** (1.21 to 1.56)**	**<0.001**
To meet friends	N=1491, M=0.44, SD=1.51	N=519, M=0.64, SD=1.20	**1.14** (1.06 to 1.24)**	**<0.001**	**1.14* (1.05 to 1.24)**	**0.002**
To meet family members who don't live with you	N=1491, M=0.42, SD=0.88	N=519, M=0.59, SD=0.95	**1.22** (1.10 to 1.35)**	**<0.001**	**1.19** (1.07 to 1.33)**	**0.001**
For a medical need (e.g., an outpatient appointment)	N=1497, M=0.04, SD=0.25	N=519, M=0.24, SD=0.60	**3.75** (2.75 to 5.11)**	**<0.001**	**3.70** (2.64 to 5.18)**	**<0.001**
To go to school	N=1491, M=0.62, SD=1.48	N=519, M=0.77, SD=1.51	1.07 (1.00 to 1.14)	0.05	1.06 (0.99 to 1.13)	0.12
For exercise	N=1491, M=3.27, SD=2.99	N=519, M=2.64, SD=2.53	**0.92** (0.89 to 0.96)**	**<0.001**	**0.93** (0.89 to 0.97)**	**<0.001**
For another reason	N=1491, M=0.15, SD=0.76	N=519, M=0.33, SD=0.97	**1.25** (1.12 to 1.40)**	**<0.001**	**1.22** (1.08 to 1.37)**	**0.001**

* *p≤ ·05* and formatted bold
** *p≤ ·001* and formatted bold
Some results are rounded to 3 rather than 2 decimal places to distinguish between *p≤ ·05* and *p≤ ·001*
† Adjusting for participant gender, age, region, household income, employment status, education level, marital status, ethnicity, and the child's gender and school year

4 DISCUSSION

During the COVID-19 pandemic, interventions were implemented to reduce physical interactions, including school closures and limits to physical contact

between members of different households. We explored the impact of these measures to identify the risk factors for children having 1) close contact with family members from outside their household and 2) a low well-being.

4.1 Physical interactions with non-household family members

Our finding that 15% of children had physical interactions with non-household family members when schools were closed is concerning, as this would have increased the risk of disease transmission. This is particularly problematic for the 6% who had interactions with a non-household family member aged 70 years or over, who would be particularly at risk from COVID-19.

We found several variables associated with non-household contact that seem to point towards a high risk of such interactions among families that require childcare. This included, for example, when one parent was a key worker and when parents reported they had been unable to keep up with work or other important commitments. At the time of the survey parents were able to go back to work and some children were eligible to attend school. However, research suggests that most eligible children were still not attending school [15], and many will have needed alternative childcare.

We found that the risk for non-household family interactions was relatively equal across all children’s ages, which contrasts with previous research [6]. We suspect that the restrictions in place at the time of our survey explains this result. A longitudinal German study found that during COVID-19, the activities that children engaged in differed between ages [21]. However, these age differences disappeared when the guidance was more restrictive. That study also found that children

commonly met elderly relatives (and friends) throughout the one-year study period, which supports our findings.

There appeared to be a cluster of predictive variables that indicated that children with worse psychological well-being were more likely to meet up with non-household family members. Variables in this group included children or their parent having a low well-being and when children being worried about non-household family members. We interpret these findings as the physical interactions may have been used to try to improve children's well-being [22], although as we are unable to determine causality; it could be that children's well-being reduced because of meeting their non-household family members.

Children who had extra support before the school closures and had special educational needs were also more likely to have non-household family interactions. This suggests that worries about education also increased risk of non-household interactions, which aligns with previous research [6].

Our results were less clear in terms of the perceived risk of COVID-19 and the impact on children's non-household family interactions. We did not find any associations between children's non-household family interactions and the lockdown statements that related to COVID-19. This finding is at odds with previous research [6]. In addition, children who were vulnerable to COVID-19 and lived with someone over 70 years old were more likely to have non-household family interactions. These findings are concerning, suggesting a higher risk of contagion in these more vulnerable groups.

4.2 Children with a low well-being

Over a quarter (26%) of children reported on in our study had a low well-being that places them at risk for a clinical mental illness. A study in Switzerland conducted while schools were closed due to the COVID-19 pandemic found children’s well-being and family functioning had reduced compared to before the pandemic, and the psychological impacts were greater for children at risk for neurodevelopmental impairments [23]. Children with special educational needs had four times higher odds of having a lower well-being compared to children without special educational needs. Previous research supports this finding [24]. In addition, a study suggests that children with special education needs are at risk for emotional, conduct, attention and peer relationship problems [25]. Therefore, the risk of adverse mental health problems could be exacerbated in children with special educational needs due to being at increased risk of challenging behaviour during lockdown. Parents who struggle to manage their children’s behaviour are prone to using adverse parenting styles, and are at increased risk of family conflict and parental distress [26-28]. That said, 'special educational needs’ covers a wide range of health and educational needs and research is needed to unpack what makes these children most at risk of poor well-being; increased risk could result from educational worries and a lack of academic support for these children [23].

We also observed risk factors for low well-being that relate to the home environment: parents who reported that their child had lower well-being also tended to report that they were bored or upset about not seeing family members, and that people in the household had not been getting along, or there was no structure to the day. Parents were also more likely to be worried about the financial impact of lockdown or unable to keep up with work and other commitments. Similar risk factors

were identified in a study conducted during the COVID-19 pandemic that found individuals with poor sleep quality, increased distress due to financial circumstances, dependents, and or who were not adjusting to lockdown were more likely to experience depression [29]. In addition, there is research showing the relationship between parent low well-being and child poor mental health outcomes [5, 26, 30]. We found that children were at seven times higher odds of lower well-being when the parent had low well-being. Our findings indicate that not only is parental well-being associated with child well-being but there also appears to be a link between parental distress due to home circumstances, such as financial worries.

Factors related to vulnerability to COVID-19 were also associated with children having a lower well-being. These findings mirror research showing that risk factors for poor well-being in children include being worried about a grandparent's vulnerability to COVID-19, infecting their grandparents [9, 10] and being vulnerable to COVID-19 themselves [31, 32]. Children were also more likely to have a lower well-being if one of their parents was a key worker. Keyworkers commonly interacted with many people daily, which increased their risk of COVID-19 [33]. Families that adopted more protective behaviours because of the risk of COVID-19 were also more likely to have a child with lower well-being; it is possible that increased levels of protective behaviours reflected a higher general sense of worry about the pandemic within the household.

More positively, children who had a higher well-being were more likely to have parents who had confidence in home-schooling and the perception that children were keeping up with their schooling. Research has shown that parental self-efficacy can improve children's well-being [34]. It is possible that this explains our results

perhaps by reducing tension within the home about schoolwork. We did not find any associations with parental education, income, or employment status in contrast to previous research [35] although we did note that younger parents were more likely to report low well-being for their children than older parents. Younger adults in general experienced higher levels of stress and anxiety during the pandemic [29] because of more challenging working and living conditions, something which might account for our findings.

Exercise was also a protective factor; children were more likely to have a higher well-being the more times they had left the home to exercise. This finding aligns with previous research that shows the benefits of exercise on physical and mental health [29, 36-39]. We were surprised that access to outside space had no associations with either of our outcomes, although having access to outside space does not necessarily mean children use the space. To counterbalance the increased stress on families as a result of the pandemic, exercise should be promoted as a way to maintain well-being and parents should be taught how to increase their self-efficacy in managing difficult situations.

4.3 Limitations

We used purposive sampling to meet pre-determined quotas for parent and children characteristics to broadly represent parents and children in England. The use of quota rather than random sampling means that it is not possible to quantify the nature of any bias in the prevalence estimates that we have made. However, we have no reason to believe that the associations between the different variables that we measured would be affected by any theoretical bias [40]. The RCADS is designed for children aged between 8 and 18 although parents reported on children

from four years old [41]. However, we suggest this has minimal impact on our findings, RCADS has been found to be a reliable and valid measure in children as young as three years old [42].

4.4 Conclusions

During the COVID-19 pandemic we found that although children reduced their physical interactions, 15% had non-adherent physical contact with non-household family members. The reasons for these interactions were largely related to a need for childcare, although factors relating to COVID-19, children's well-being and education were also important. Children who had special educational needs or had a parent with low well-being were most at risk for having a lower well-being themselves. Exercise and parent self-efficacy with home-schooling may help maintain children's mental and physical health.

Keywords: children; COVID-19; close contact; physical interactions; adherence; England; parents; well-being; mental health; physical health; exercise

Supplementary materials: The following supporting information can be downloaded at: DOI 10.18742/21757232, S.1 Text: Full survey material.

Author contributions:

Lisa Woodland: Conceptulization, Methodology, Formal analysis, Investigation, Data Curation, Writing – Original Draft, Visualization, Project administration.

Louise E. Smith: Conceptulization, Methodology, Writing – Review & Editing.

Samantha K. Brooks: Methodology, Writing – Review & Editing.

Rebecca K. Webster: Conceptulization, Methodology, Writing – Review & Editing, Supervision, Funding acquisition.

Richard Amlôt: Conceptulization, Methodology, Writing – Review & Editing, Supervision, Funding acquisition.

Antonia Rubin: Investigation, Methodology, Writing – Review & Editing.

G. James Rubin: Conceptulization, Methodology, Resources, Writing – Review & Editing, Supervision, Funding acquisition.

Competing Interests: I have read the journal's policy and the authors of this manuscript have the following competing interests: G.J.R., R.A. and L.S. participate in the UK's Scientific Advisory Group for Emergencies, or its subgroups. RA is an employee of the UK Health Security Agency. These groups did not fund the study or authors. The author interests do not alter our adherence to PLOS ONE policies on sharing data and materials.

Funding: This study was funded by the Economic and Social Research Council [grant number ES/P000703/1] and by the National Institute for Health and Care Research Health Protection Research Unit (NIHR HPRU) in Emergency Preparedness and Response [grant number NIHR200890], a partnership between the UK Health Security Agency, King's College London and the University of East Anglia. The views expressed are those of the author(s) and not necessarily those of the NIHR, UKHSA or the Department of Health and Social Care. For the purpose of open access, the author has applied a Creative Commons Attribution (CC BY) licence to any Author Accepted Manuscript version arising

Role of the funding source: The funders had no role in study design, data collection and analysis, decision to publish, or preparation of the manuscript. The corresponding author had full access to all the data and had final responsibility for the decision to submit for publication.

Data Sharing: The data set and survey questions related to this study can be accessed via the King's College London research data repository, KORDS (DOI 10.18742/21757232). For further information contact: kcl - research.data research.data@kcl.ac.uk.

APPENDIX A

Table 6 Frequencies to show the behaviours that parents or children had followed, in the past 7 days because of the risk of coronavirus. The findings our presented by children's non-household family interactions (n=309) and lower well-being (n=519).

Statements	Statement in relation to COVID-19 guidelines at the time	Total participants (n=2010, %)	Childs not had family interactions (n=1701, %)	Childs had family interactions (n=309, (%)	Child higher well-being, (n=1491, %)	Child lower well-being, (n=519, %)
Washed your hands thoroughly and regularly	Recommended	1895 (94%)	1617 (95)	278 (90)	1435 (96)	460 (89)
Stayed 2m (3 steps) away from people you do not live with when outside your home	Recommended	1867 (93%)	1599 (94)	268 (87)	1413 (95)	454 (87)
Cleaned or disinfected items you have brought into the home, such as groceries or parcels	Not recommended	1089 (54%)	908 (53)	181 (59)	779 (52)	310 (60)
Washed [CHILD]'s clothes when she/ he has returned home	Not recommended	1089 (54%)	829 (49)	176 (57)	688 (46)	317 (61)
Washed your clothes when you have returned home	Not recommended	959 (48%)	788 (46)	171 (55)	653 (44)	306 (59)
Used tissues or clothing to touch door handles or other objects when outside	Not recommended	954 (48%)	788 (46)	166 (54)	679 (46)	275 (53)
Covered your face or used a face mask when out and about	Not recommended	822 (41%)	678 (40)	144 (47)	571 (38)	251 (48)
Covered [CHILD's] face or given her/him a face mask when out and about	Not recommended	741 (37%)	600 (35)	141 (46)	500 (34)	241 (46)
Left items that you have brought into the home for 24 hours or more before using them, to kill any virus on them	Not recommended	725 (36%)	607 (36)	118 (38)	493 (33)	232 (45)
Worn protective gloves when out and about	Not recommended	693 (35%)	562 (33)	131 (43)	453 (30)	240 (46)
Made [CHILD] wear protective gloves when out and about	Not recommended	503 (25%)	390 (23)	113 (37)	308 (21)	195 (38)

5 REFERENCES

1. HM Government Prime Minister's statement on coronavirus (COVID-19): 23 March 2020. https://www.gov.uk/government/speeches/pm-address-to-the-nation-on-coronavirus-23-march-2020 (accessed 11 June 2020).
2. HM Government Children of critical workers and vulnerable children who can access schools or educational settings. https://www.gov.uk/government/publications/coronavirus-covid-19-maintaining-

educational-provision/guidance-for-schools-colleges-and-local-authorities-on-maintaining-educational-provision (accessed 16 March 2021).
3. HM Government Prime Minister's statement on coronavirus (COVID-19): 11 May 2020. https://www.gov.uk/government/speeches/pm-statement-on-coronavirus-11-may-2020 (accessed 22 March 2022).
4. HM Government, PM: Six people can meet outside under new measures to ease lockdown. 2020.
5. Viner, R.; Russell, S.; Saulle, R.; Croker, H.; Stansfield, C.; Packer, J.; Nicholls, D.; Goddings, A.-L.; Bonell, C.; Hudson, L.; Hope, S.; Ward, J.; Schwalbe, N.; Morgan, A.; Minozzi, S., School Closures During Social Lockdown and Mental Health, Health Behaviors, and Well-being Among Children and Adolescents During the First COVID-19 Wave. *JAMA Pediatrics* **2022**.
6. Brooks, S. K.; Smith, L. E.; Webster, R. K.; Weston, D.; Woodland, L.; Hall, I.; Rubin, G. J., The impact of unplanned school closure on children's social contact: rapid evidence review. *Euro Surveill* **2020,** *25* (13).
7. Mizumoto, K.; Yamamoto, T.; Nishiura, H., Contact behaviour of children and parental employment behaviour during school closures against the pandemic influenza A (H1N1-2009) in Japan. *Journal of International Medical Research* **2013,** *41* (3), 716-724.
8. HM Government, Sixteenth SAGE meeting on Wuhan Coronavirus (Covid-19), 16th March 2020. 2020.
9. Sarkadi, A.; Sahlin Torp, L.; Pérez-Aronsson, A.; Warner, G., Children’s expressions of worry during the COVID-19 pandemic in Sweden. *Journal of Pediatric Psychology* **2021,** *46* (8), 939-949.
10. Idoiaga, N.; Berasategi, N.; Eiguren, A.; Picaza, M., Exploring children’s social and emotional representations of the Covid-19 pandemic. *Frontiers in Psychology* **2020,** *11*, 1952.
11. Denford, S.; Morton, K. S.; Lambert, H.; Zhang, J.; Smith, L. E.; Rubin, G. J.; Cai, S.; Zhang, T.; Robin, C.; Lasseter, G.; Hickman, M.; Oliver, I.; Yardley, L., Understanding patterns of adherence to COVID-19 mitigation measures: a qualitative interview study. *Journal of Public Health* **2021,** *43* (3), 508-516.
12. Lasseter, G.; Compston, P.; Robin, C.; Lambert, H.; Hickman, M.; Denford, S.; Reynolds, R.; Zhang, J.; Cai, S.; Zhang, T.; Smith, L. E.; Rubin, J.; Yardley, L.; Amlot, R.; Oliver, I., Exploring the impact of shielding advice on the health and wellbeing of individuals identified as extremely vulnerable and advised to shield in Southwest England amid the COVID-19 pandemic: A mixed-methods evaluation. Cold Spring Harbor Laboratory: 2022.
13. BMG Research BMG About Us. https://www.bmgresearch.co.uk/bmg/about-us/ (accessed 1 September 2022).
14. Smith, L. E.; Woodland, L.; Amlôt, R.; Rubin, A.; Rubin, G. J., A cross-sectional survey of parental perceptions of COVID-19 related hygiene measures within schools and adherence to social distancing in journeys to and from school. *BMJ Paediatrics Open* **2020,** *4* (1).
15. Woodland, L.; Smith, L. E.; Webster, R. K.; Amlôt, R.; Rubin, A.; Wessely, S.; Rubin, J. G., Why did some parents not send their children back to school following school closures during the COVID-19 pandemic: a cross-sectional survey. *BMJ Paediatrics Open* **2021,** *5* (1), e001014.
16. HM Government Schools, pupils and their characteristics: Academic Year 2019/20. https://explore-education-statistics.service.gov.uk/find-statistics/school-pupils-and-their-characteristics (accessed 25 June 2020).

17. Chorpita, B. F.; Yim, L.; Moffitt, C.; Umemoto, L. A.; Francis, S. E., Assessment of symptoms of DSM-IV anxiety and depression in children: a revised child anxiety and depression scale. *Behaviour Research and Therapy* **2000,** *38* (8), 835-855.

18. Kroenke, K.; Spitzer, R. L.; Williams, J. B. W.; Lowe, B., An Ultra-Brief Screening Scale for Anxiety and Depression: The PHQ-4. *Psychosomatics* **2009,** *50* (6), 613-621.

19. IBM Corp IBM SPSS Statistics for Windows, 26.0 Armonk, NY.

20. IBM Corp IBM SPSS Statistics for Windows, 28.0 Armonk, NY.

21. Paulsen, M.; Zychlinsky Scharff, A.; De Cassan, K.; Sugianto, R. I.; Blume, C.; Blume, H.; Christmann, M.; Hauß, C.; Illig, T.; Jonczyk, R.; Klopp, N.; Kopfnagel, V.; Lichtinghagen, R.; Lucas, H.; Luhr, A.; Mutschler, F.; Pietschmann, T.; Pott, P.-C.; Prokein, J.; Schaefer, P.; Stahl, F.; Stanislawski, N.; Von Der Born, J.; Schmidt, B. M. W.; Heiden, S.; Stiesch, M.; Memaran, N.; Melk, A., Children and Adolescents' Behavioral Patterns in Response to Escalating COVID-19 Restriction Reveal Sex and Age Differences. *Journal of Adolescent Health* **2022,** *70* (3), 378-386.

22. Morina, N.; Kip, A.; Hoppen, T. H.; Priebe, S.; Meyer, T., Potential impact of physical distancing on physical and mental health: a rapid narrative umbrella review of meta-analyses on the link between social connection and health. *BMJ Open* **2021,** *11* (3), e042335.

23. Ehrler, M.; Werninger, I.; Schnider, B.; Eichelberger, D. A.; Naef, N.; Disselhoff, V.; Kretschmar, O.; Hagmann, C. F.; Latal, B.; Wehrle, F. M., Impact of the COVID-19 pandemic on children with and without risk for neurodevelopmental impairments. *Acta Paediatrica* **2021,** *110* (4), 1281-1288.

24. Essex, M. J.; Kraemer, H. C.; Armstrong, J. M.; Boyce, W. T.; Goldsmith, H. H.; Klein, M. H.; Woodward, H.; Kupfer, D. J., Exploring Risk Factors for the Emergence of Children's Mental Health Problems. *Archives of General Psychiatry* **2006,** *63* (11), 1246.

25. Deighton, J.; Lereya, S. T.; Casey, P.; Patalay, P.; Humphrey, N.; Wolpert, M., Prevalence of mental health problems in schools: poverty and other risk factors among 28 000 adolescents in England. *British Journal of Psychiatry* **2019,** *215* (3), 565-567.

26. Wille, N.; Bettge, S.; Ravens-Sieberer, U., Risk and protective factors for children's and adolescents' mental health: results of the BELLA study. *European Child & Adolescent Psychiatry* **2008,** *17* (S1), 133-147.

27. Prime, H.; Wade, M.; Browne, D. T., Risk and resilience in family well-being during the COVID-19 pandemic. *American Psychologist* **2020,** *75* (5), 631.

28. Spinelli, M.; Lionetti, F.; Pastore, M.; Fasolo, M., Parents' stress and children's psychological problems in families facing the COVID-19 outbreak in Italy. *Frontiers in psychology* **2020,** *11*, 1713.

29. Varma, P.; Junge, M.; Meaklim, H.; Jackson, M. L., Younger people are more vulnerable to stress, anxiety and depression during COVID-19 pandemic: A global cross-sectional survey. *Progress in Neuro-Psychopharmacology and Biological Psychiatry* **2021,** *109*, 110236.

30. Davis, E.; Sawyer, M. G.; Lo, S. K.; Priest, N.; Wake, M., Socioeconomic risk factors for mental health problems in 4–5-year-old children: Australian population study. *Academic Pediatrics* **2010,** *10* (1), 41-47.

31. Tunçgenç, B.; Newson, M.; Sulik, J.; Zhao, Y.; Dezecache, G.; Deroy, O.; Zein, M. E., Social alignment matters: Following pandemic guidelines is associated with better wellbeing. *BMC Public Health* **2022,** *22* (1).
32. Saurabh, K.; Ranjan, S., Compliance and Psychological Impact of Quarantine in Children and Adolescents due to Covid-19 Pandemic. *The Indian Journal of Pediatrics* **2020,** *87* (7), 532-536.
33. Environmental Modelling Group (EMG) Transmission Group *COVID-19 Risk by Occupation and Workplace*; HM Government: 11 February, 2021.
34. Albanese, A. M.; Russo, G. R.; Geller, P. A., The role of parental self-efficacy in parent and child well-being: A systematic review of associated outcomes. *Child: care, health and development* **2019,** *45* (3), 333-363.
35. Reiss, F., Socioeconomic inequalities and mental health problems in children and adolescents: A systematic review. *Social Science & Medicine* **2013,** *90*, 24-31.
36. Biddle, S. J. H.; Ciaccioni, S.; Thomas, G.; Vergeer, I., Physical activity and mental health in children and adolescents: An updated review of reviews and an analysis of causality. *Psychology of Sport and Exercise* **2019,** *42*, 146-155.
37. Ahn, S.; Fedewa, A. L., A Meta-analysis of the Relationship Between Children's Physical Activity and Mental Health. *Journal of Pediatric Psychology* **2011,** *36* (4), 385-397.
38. Eddolls, W. T. B.; McNarry, M. A.; Lester, L.; Winn, C. O. N.; Stratton, G.; Mackintosh, K. A., The association between physical activity, fitness and body mass index on mental well-being and quality of life in adolescents. *Quality of Life Research* **2018,** *27* (9), 2313-2320.
39. Benzing, V.; Gaillard, P.; Scheidegger, D.; Dössegger, A.; Nigg, C. R.; Schmidt, M., COVID-19: Physical Activity and Quality of Life in a Sample of Swiss School Children during and after the First Stay-at-Home. *International Journal of Environmental Research and Public Health* **2022,** *19* (4), 2231.
40. Kohler U Possible Uses of Nonprobability Sampling for the Social Sciences. *Survey Methods: Insights from the Field*. https://surveyinsights.org/?p=10981 (accessed 29 July 2020).
41. Ebesutani, C.; Chorpita, B. F.; Higa-Mcmillan, C. K.; Nakamura, B. J.; Regan, J.; Lynch, R. E., A Psychometric Analysis of the Revised Child Anxiety and Depression Scales—Parent Version in a School Sample. *Journal of Abnormal Child Psychology* **2011,** *39* (2), 173-185.
42. Ebesutani, C.; Tottenham, N.; Chorpita, B., The Revised Child Anxiety and Depression Scale - Parent Version: Extended Applicability and Validity for Use with Younger Youth and Children with Histories of Early-Life Caregiver Neglect. *Journal of Psychopathology and Behavioral Assessment* **2015,** *37* (4), 705-718.

Click here to access/download
Supporting Information
S.1 Text.docx
