# Supplementary material for: Parent-reported child’s close contact with non-household family members and their well-being during the COVID-19 pandemic: A cross-sectional survey
Source: PLoS One. 2023 Oct 19;18(10):e0292344. doi: 10.1371/journal.pone.0292344 (PMC10586646; doi:10.1371/journal.pone.0292344)
Supplement: Attachment — Submitted filename: PONE-D-23-01008_R1+Reviewed.pdf [file pone.0292344.s005.pdf]

PLOS ONE

Parent-reported child's close contact with non-household family members and their well-being during the COVID-19 pandemic: a cross-sectional survey

--Manuscript Draft--

**Manuscript Number:**	PONE-D-23-01008R1
**Article Type:**	Research Article
**Full Title:**	Parent-reported child's close contact with non-household family members and their well-being during the COVID-19 pandemic: a cross-sectional survey
**Short Title:**	Children's perceived close contact with non-household family
**Corresponding Author:**	Lisa Woodland<br>King's College London<br>London, London UNITED KINGDOM
**Keywords:**	children; COVID-19; close contact; physical interactions; adherence; England; parents; well-being; mental health; physical health; Exercise
**Abstract:**	In England (UK), at the start of the COVID-19 pandemic the public were required to reduce their physical contacts to slow the spread of COVID-19. We investigated risk factors for children having: 1) close contact with family members from outside their household ("non-adherent behaviour"); and 2) low well-being (using the Revised Child Anxiety and Depression Scale). We conducted an online cross-sectional survey, completed at any location of the participant's choice between 8 and 11 June 2020 in parents (n = 2,010) who were aged eighteen years or over and had a school-aged child (4-18 years old). Parents reported that 15% (n = 309) of children had non-adherent contact and that 26% (n = 519) had low well-being. We used a series of binary logistic regressions to investigate associations between outcomes and child and parent characteristics. Children had higher odds of having non-household contact when they had special educational needs [adjusted odds ratio, 2.19 (95% CI, 1.47 to 3.27)], lower well-being [2.65 (95% CI, 2.03 to 3.46)], were vulnerable to COVID-19 [2.17 (95% CI, 1.45 to 3.25)], lived with someone who was over 70 years old [2.56 (95% CI, 1.55 to 4.24)] and their parent had low well-being [1.94 (95% CI, 1.45 to 2.58)]. Children had higher odds of lower well-being when they had special educational needs [4.13 (95% CI, 2.90 to 5.87)], were vulnerable to COVID-19 [3.06 (95% CI, 2.15 to 4.36)], lived with someone else who was vulnerable to COVID-19 [2.08 (95% CI, 1.64 to 2.64)], or lived with someone who was over 70 years old [2.41 (95% CI, 1.51 to 3.83)]. Many children came into contact with non-household family members, mainly for childcare. Factors relating to COVID-19, children's well-being and education were also important. If school closures are needed in future, addressing these issues may help reduce contact.
**Order of Authors:**	Lisa Woodland
	Louise E Smith
	Samantha K Brooks
	Rebecca K Webster
	Richard Amlôt
	Antonia Rubin
	G James Rubin
**Opposed Reviewers:**	
**Response to Reviewers:**	1. Please ensure that your manuscript meets PLOS ONE's style requirements, including those for file naming. The PLOS ONE style templates can be found at https://journals.plos.org/plosone/s/file?id=wjVg/PLOSOne_formatting_sample_main_body.pdf and https://journals.plos.org/plosone/s/file?id=ba62/PLOSOne_formatting_sample_title_authors_affiliations.pdf.<br>Response: We have updated the manuscript to align with PLOS ONE's formatting

Powered by Editorial Manager® and ProduXion Manager® from Aries Systems Corporation

style.
2. Please provide additional details regarding participant consent. In the ethics statement in the Methods and online submission information, please ensure that you have specified (1) whether consent was informed and (2) what type you obtained (for instance, written or verbal, and if verbal, how it was documented and witnessed). If your study included minors, state whether you obtained consent from parents or guardians. If the need for consent was waived by the ethics committee, please include this information.
If you are reporting a retrospective study of medical records or archived samples, please ensure that you have discussed whether all data were fully anonymized before you accessed them and/or whether the IRB or ethics committee waived the requirement for informed consent. If patients provided informed written consent to have data from their medical records used in research, please include this information.
Response: We have provided more information about how the participants provided consent.
3. Thank you for stating the following in the Competing Interests section:
'I have read the journal's policy and the authors of this manuscript have the following competing interests: G.J.R., R.A. and L.S. participate in the UK's Scientific Advisory Group for Emergencies, or its subgroups. RA is an employee of the UK Health Security Agency. These groups did not fund the study or authors. The author interests do not alter our adherence to PLOS ONE policies on sharing data and materials."
We note that one or more of the authors are employed by a commercial company: UK Health Security Agency
a. Please provide an amended Funding Statement declaring this commercial affiliation, as well as a statement regarding the Role of Funders in your study. If the funding organization did not play a role in the study design, data collection and analysis, decision to publish, or preparation of the manuscript and only provided financial support in the form of authors' salaries and/or research materials, please review your statements relating to the author contributions, and ensure you have specifically and accurately indicated the role(s) that these authors had in your study. You can update author roles in the Author Contributions section of the online submission form.
Please also include the following statement within your amended Funding Statement. "The funder provided support in the form of salaries for authors [insert relevant initials], but did not have any additional role in the study design, data collection and analysis, decision to publish, or preparation of the manuscript. The specific roles of these authors are articulated in the 'author contributions' section."
If your commercial affiliation did play a role in your study, please state and explain this role within your updated Funding Statement.
Response: We have included the funding statement that you have provided and updated the funding statement to include that one of the authors is affiliated with UK Health Security Agency which is an arm of the UK Government.
b. Please also provide an updated Competing Interests Statement declaring this commercial affiliation along with any other relevant declarations relating to employment, consultancy, patents, products in development, or marketed products, etc.
Within your Competing Interests Statement, please confirm that this commercial affiliation does not alter your adherence to all PLOS ONE policies on sharing data and materials by including the following statement: "This does not alter our adherence to PLOS ONE policies on sharing data and materials." (as detailed online in our guide for authors http://journals.plos.org/plosone/s/competing-interests) . If this adherence statement is not accurate and there are restrictions on sharing of data and/or materials, please state these. Please note that we cannot proceed with consideration of your article until this information has been declared.
Please include both an updated Funding Statement and Competing Interests Statement in your cover letter. We will change the online submission form on your behalf.
Response: We have updated the competing interests statement to include the statement that you have provided.
Academic Editor's Comments:
Title:
1.Since the children were not the respondents in this survey, the title should be rephrased to reflect that. Secondly, substitute "interaction" with "close contact" for consistency. The suggested/rephrased title is: "Perceived children's close contact with

	non-household family members and their well-being during the COVID-19 pandemic: a cross-sectional survey"<br>Response: We have changed the title to "Parent-reported child close contact with non-household family members and their well-being during the COVID-19 pandemic: a cross-sectional survey," to make it clear that it was parents and not children who responded to the survey.<br>Abstract:<br>2.Line 28: Is close contact = interaction? It is not clear. Be consistent throughout the article.<br>Response: We have updated the manuscript throughout to use "close contact" rather than interaction for clarity.<br>3.Line 32: mention the setting and highest statistical procedure/tests performed.<br>Response: We have added to the abstract that the cross-sectional survey was "completed at any location" by the participants and the statistical tests that we used.<br>4.Lines 34-35: Include results of direct reports of interactions/contact by the students as well, not only as perceived by their parents.<br>Response: Because all data are based on parental report, we do not have these data. We have made it clearer that the findings are from parents' perceptions about their child's behaviour.<br>5.Line 34: Revisit the statistical language: odds ratios are not a measure of risk, therefore, revise the phrase "more likely" to "had higher odds" throughout the manuscript.<br>Response: We have revised our manuscript throughout to report "had higher odds."<br>6.Liness 34-40: state the statistical values e.g. aORs and their Cis.<br>Response: We have added the adjusted odd ratio and confidence intervals for each result.<br>Include a sentence that will serve as a "conclusion and recommendation" in the abstract.<br>Response: We have added a conclusion and recommendation to the abstract.<br>8.Include key words after the abstract.<br>Response: We have added six key words after the abstract.<br>Introduction<br>9.Do not number the headings for PLOS One (revise the manuscript by strictly following the journal formatting style)<br>Response: We have updated the manuscript to follow PLOS One's formatting style.<br>10.Line 54 should be "Box 1". However, it is not clear what this text appears in a box when it is still the authors presenting their introduction. It would make sense if it was an image from another source document e.g., the media or regulating agency. I suggest this be removed from the box and be presented as part of the continuous writing of the introduction and be cited as done in the box.<br>Response: We have removed the box and added the text to follow on the first paragraph in the introduction.<br>11.Lines 100-104, why these variables, specifically, amongst all other 'risk factors'? What informed this selection?<br>Response: We have added that the variables we investigated were to chosen because we had conducted a qualitative study that suggested these variables may have an impact on children's well-being.<br>12.Discuss the known risk factors from previous studies in the introduction section. What factors have other studies identified? Any disagreements/mixed findings on these?<br>Response: We have reported the risk factors that have been identified in previous research.<br>13.Discuss the "well-being" reported in other studies from elsewhere as part of the literature review in the introduction section.<br>Response: We have added more detail about well-being from the systematic review that we reported about.<br>Methods:<br>14.Lines 106 and 117, BMG stands for?<br>Response: BMG does not stand for anything – it is the registered name of the company. Information about the company can be found in the reference that we have cited.<br>15.Line 122, if you begin a sentence with a number, write the number in words. Do that throughout the manuscript.<br>Response: We have updated this throughout the manuscript.

16.Lines 124-125, "… completing the survey quickly…".   – What was the definition of "quickly"? Who assessed the quickness, BMG or the authors? How did the authors ascertain this?
Response: We have clarified that BMG screened out the non-eligible participants and that "quickly" was compared against the average and median survey time.
17.Lines 124-126, indicate to readers whether this practice is common/conventional in such studies i.e. cite previous studies that have used or textbooks that recommend such practice.
Response: We have provided a reference which defines this as a common problem.
Line 132, upload the Ethical clearance certificate during the resubmission.
Response: We have provided the ethical clearance with the submission.
Line 134, was the questionnaire/survey administered online or physically? On average, how long did it take participants to complete the survey?
Response: We have reported that the survey was administered online and provided information about how long the survey took to complete.
20.Line 147, decide whether to use "interaction" or "close contact"? Be consistent.
Response: We have changed all wording to close contact.
Line 155, what is the validity of this question in measuring "physical interaction" or close contact?
Response: The survey copied the wording of the guidance given by the UK Government to determine close contact which we have reported in the study and provided a reference. But beyond face validity we do not have data on the association between self-reports to this item and observed close contact. We have added to the limitations that the study used self-report data.
22.Lines 156-165, discuss the scoring, validity and reliability of the measure. In fact, do that for all the measures in your study.
Response: We have added a validity and reliability section that has references to show the reliability of the RCADS and PHQ-4. We have also reported that our other study questions have face validity, although we do not have psychometric data for them.
23.Line 179, where were these questions sourced from? Or were they developed for this study? How or informed by?
Response: We have reported that the questions that related to COVID-19 were designed for this study and were based on our previous study, which is the same study that we used to design our predictor variables and have now referenced in the introduction.
24.Line 186, Who is the "patient" being referred to here?
Response: We have removed "patient" from the sub-heading.
25.Lines 192-236, "Kill" this section by merging it with 2.3: analysis should be discussed under 2.6. This will enhance the flow.
Response: We have merged the methods section as you have suggested.
26.Lines 197-200, what informed this definition of close contact or interaction?
Response: We have reported that we used the UK Government's definition of close contact and referenced this.
27.Lines 213-215, recoded from what original categorization? Or from continuous measurement? Specify that for all the variables described as "recoded" below.
Response: We had provided the categorization for each question that were originally reported in lines 213-215, this information can also be found in the full survey materials which has been provided as supplementary materials.
28.Lines 229-232, which ones are these now? Are they the same questions referred-to in 2.3.6? If so, merge the sections with 2.3.6. If different, which ones are these now? What questions were asked? How were they developed? What is their validity?
Response: We suggest that this comment has been resolved from merging the method sections and adding a validity and reliability section in response to previous comments.
Lines 212-236, these should be discussed under 2.3.
Response: We have merged the method section as you have previously suggested.
30.Line 233, what was the scoring and the possible score range? Merge/discuss this under 2.3.
Response: We have merged the method section as you have previously suggested.
31.Lines 235-236, what informed this statistical decision? Why were they not valid responses? Is this common statistical practice? If so, site the authorities or sources recommending such a practice. How many of such observations were deemed as missing? Did you assess the impact of removing these observations?
Response: We have added to the manuscript that the reason we adopted this approach for outcome variables was because it was not possible to categorise

Powered by Editorial Manager® and ProduXion Manager® from Aries Systems Corporation

	participants as adherent or not, or as having good wellbeing or not. For predictors we adopted this approach to provide a clearer understanding of the difference between endorsing, or not endorsing, each variable in terms of its impact on our outcomes.<br>32.Line 236, so, what was the eventual response rate in this study?<br>Response: Quota sampling is a non-probability based approach. Response rates are therefore not helpful – the rates for each quota will differ, the denominator for each is essentially is unknown, and the predominant source of bias is in the make up of the underlying panels that are being recruited from, rather than whether the sample is representative of the panel. We have now reminded readers about this in the paper and given a reference for further reading.<br>33.Line 240, it appears as though hierarchical models would have been the best choice/more efficient here to minimize/control Type I error. Reconsider your analysis decisions/choices and justify your decisions or choices. Otherwise, rerun the analysis using hierarchical models.<br>Response: We agree with the reviewer that using hierarchical models would have helped minimise Type I error. The aim of this study was to investigate independent factors that may have been associated with outcomes, so that we could make specific suggestions to public health agencies for potential targets of communications or policy interventions. We based our inclusion of predictive factors on previous research (published and conducted by our group). Factors investigated were wide ranging, from parent and child personal and clinical characteristics, to parent perceptions of COVID-19, home-schooling, child perceptions of COVID-19, coping in the last week, and number of times having left the home. If we had used hierarchical models, we would have been investigating the effect of a factor, while controlling for all other factors. We are not sure whether e.g., parental perceptions about home schooling are associated with the number of outings in the last week. Therefore, we did not feel comfortable including all factors in a single statistical model. Instead, we chose to control only for parent and child personal characteristics, as these are things that are not modifiable. Furthermore, there is no theoretical basis for the order of inclusion of factors in blocks, if we were to use hierarchical models. For these reasons, we chose to conduct analyses as described in the manuscript and did not use hierarchical models.<br>Results:<br>34.Line 248, present the participants' background characteristics first.<br>Response: We have reported some parent characteristics narratively in the opening paragraph and added a table reporting parent and child characteristics.<br>35.Line 249-250, you cannot report the findings as though they were stated by the children. In such studies, indicate that the results were reported or perceived by the parents, e.g., here, it must read, ".... 15% of the children were reported to have had ......".<br>Response: We have added “perceived by the parents” throughout the results to make it clear that it was not the children who responded to the study.<br>Line 256, as indicated in the comments for the abstract section, revisit your statistical language. Using “more likely” is not accurate reporting for odds ratios as they are not a measure of risk. Revise all such words and instead refer to them as "higher odds" or "lower odds" as appropriate. Do that through throughout the results and discussion sections.<br>Response: We have amended as you have suggested.<br>Ordinarily, this should be a purely descriptive table of the background characteristics of the participants.<br>Response: The tables provided all of the details necessary to understand the association between the predictor and outcome variables. However, we have now changed this so that we have one table that only reports parent and child characteristics and associations are reported in two another tables.<br>38.Throughout the results section, report the ORs and their CIs in text when reporting the results from regression.<br>Response: These are listed in the tables, and we felt repeating the information in the text would be duplicative, but we have now made this change as requested.<br>39.Indicate whether it is crude or adjusted (i.e Bivariate or multiple). Do that for all tables<br>Response: The tables already show both adjusted and unadjusted associations, although we have added “unadjusted” odds ratio to make this clearer.<br>Show the "N" for all the tables.<br>Response: The tables already show the sample sizes and percentages. For the continuous variables we have also reported the mean and standard deviation. We

	have added abbreviations to the footnotes of each table to make this clearer.<br>41.Line 269, ordinarily, this should be a purely descriptive table of the background characteristics of the participants.<br>Response: We have amended how the tables are presented following reviewer comments.<br>42.Line 274, it is not accurate to state that all these were adjusted for, yet they were also the predictors. Presenting such a statement implies that there was one or two main independent variable(s) and the rest were control variable/confounders, in which case, you would not interpret them. However, in your study, these were all covariates or predictors, hence, when interpreting one predictor, you should state that you controlled for the rest; not that the model adjusted for all the variables listed. Revisit this.<br>Response: We have changed the footnote to state that "when interpreting the predictor listed in the row, we controlled for the other variables listed" as you have suggested.<br>43.Lines, 292-294, the correct presentation should be: "parents aged between 18 and 35 years old, parents who were a key worker and parents with lower well-being had higher odds of reporting that their child had lower well-being." You must do this correction throughout the results section and discussion section.<br>Response: We have updated the results section as you have suggested.<br>44.The Tables needs to be simplified to make them easier to read. Present fewer columns, by separately presenting the background characteristics, then descriptives of the variables in Tables 4 and 5 (as Supplementary files/Supporting Information) and collapse Tables 4 and 5 as suggested below.<br>Response: We have separated the tables and the outcome frequencies are reported in the appendix to reduce the number of columns that are presented in the tables in the main text.<br>45.Tables 4 & 5, is it possible to create composite scores of each variable and present them rather than 'dumping' all the individual items into the regression models, which is what appears to be the case in the current presentation. That is, these would then be two variables represented each table.<br>Response: For similar reasons to why we did not use hierarchical models, the study aims to investigate the independent associations between the predictor variables that we reported about and our two outcomes. Therefore, a factor analysis or similar method to create a composite score of similar variables did not suit our study's aims, by combining variables we would not be able to investigate the independent effects of each predictor variable on parents' perception of their children's close contact behaviour and well-being. By doing so now would also mean that we would be revising our analytic strategy after reviewing our data. In addition, future studies will more easily be able to replicate single items rather than a composite score as they can choose the variables that they want to investigate.<br>Discussion<br>The discussion section will have to be revised to align with the changes suggested above.<br>Response: We have provided the rationale for why we have not altered our analysis strategy as suggested above. Therefore, we suggest that the discussion appropriately discusses our findings.<br>Reviewer #1:<br>1.The authors have chosen an interesting topic. The concept of the paper is so smart, starting with the introduction, methodology, discussion to a conclusion.<br>2.In the limitation part of the paper, you better to mention that using the cross sectional survey as the major limitation of this paper.<br>Response: We have added that we used a cross-sectional design to our limitations.<br>Reviewer #2:<br>1.Raw data for participant characteristics at baseline? Looking at the survey questions, you have collected a lot of important data but a lot of it is unreported here. I appreciate it may be contained in other publications or future manuscripts, but you need to contextualize your findings.<br>Response: We are not clear what the reviewer means by "baseline" – this was a cross-sectional survey or the data that they indicate we have not reported. However, we do now report a separate table of participant characteristics as suggested by the editor.<br>2.Table 1: check grammar, specify child vs parental age in the column. Add child age: what was the range? This would have had a very significant impact on interpreting results.<br>Response: We have added 'parent' or 'child' to the gender, age and school year

	variables description in the table to make this clearer. The eligibility criteria states the age of the children who were included in the study ("were a parent or guardian to a school-aged child (4-18 years old")). The table that reports parent and child characteristics that we have created following reviewer comments reports the parents age group and the child's Key Stage. We have also added the child's age range to this table to make the child's age clearer.<br>3.Did you conduct sub analyses for income as well? The income range and IQR should be reported. Grouping all participants in this way may appear to produce spurious associations. Sub-analyses for each factor (i.e. income, age) would provide more clarity.<br>Response: We agree that you can code variables in various different ways and each method may produce different results. Therefore, the decisions that we made about how we would code our variables were made carefully based on previous research and our data before we conducted our analysis. The number of levels in a variable was a main driver, we tried to balance not having too many levels as not to increase the risk of type 2 errors with being able to identify the groups most at risk. There were 15 different income brackets and one "prefer not to say," we decided to create a binary variable based on the mean household income in our data (£40,000 to £44,000 bracket) and the mean UK household income in the UK (£32,000). Providing the IQR for income would not be useful as this was a categorical variable. We have conducted sensitivity analysis about this variable, which show a similar pattern to the findings that we have presented.<br>We decided to recode age, into three levels based on the Mean = 43 years of age; 25th percentile = 37 years of age; and 75th percentile = 49 years of age and with trying to group the income brackets evenly (by number of responses and income range). Previous research also indicated that very young or old parents may have difference impacts on our outcomes compared to middle aged parents and therefore, keeping the age variable as a continuous variable did not align to our study's aims. We did not run sensitivity analysis on these data as increasing the number of levels in the age variable, by increments of 10 years for example produces low cell counts in several categories and therefore we are unable to analyse the data.<br>4.Other comments about tables in general: the way the data are presented is confusing. For example, you report % across columns but then use reference ranges across rows (86% of male parents completed the survey about children who had not had family interactions, then appears you report an odds ratio calculated relative to female parents?)<br>Response: The reviewer has correctly interpreted how we had presented the table. However, we have now split them up into multiple tables at the suggestion of the editor so that the tables in the main manuscript have fewer columns for clarity.<br>5.You state in the limitations section that you could not adjust for bias created by sampling, why was this the case and what happened when these variables were included in the model?<br>Response: We are not certain we understand the reviewer's point here. We used quota, rather than random sampling. This might lead to bias. There is no analysis we can do to adjust for this – the bias is inherent in the sampling strategy we used. The best we can do is to be honest with readers about the limitation.<br>6.What about the incidence of infection? Medical conditions? What was the definition of special needs/ were they all similar?<br>Response: We did not assess the incidence of infection, rather parents' perceptions about their or their child's perceptions of COVID-19 (e.g., child is worried about COVID-19 and if child goes out, she/he is likely to catch COVID-19) to identify how these perceptions may or may not influence children's contact behaviour and well-being. Similarly, the importance of the study was not the type of medical conditions that the child or household member had that made them vulnerable to COVID-19 and if the child had special educational needs, rather whether or not these perceptions influenced behaviour. We asked the parent "does the [child] have special educational needs," which we also discuss in the discussion: "special educational needs covers a wide range of health and educational needs and research is needed to unpack what makes these children most at risk of poor well-being."<br>7.Minor point: participant reimbursement is unclear: what are points?<br>Response: Panel members earn points for each survey they complete. They can then exchange their accumulated points for rewards once they have accumulated enough (that is, once they have completed enough surveys). For our survey, they earned points worth about 60p. We have added this to the manuscript.

	8.Conclusion that this places children at risk of clinical mental illness is unfounded. Responses to COVID 19 are social phenomena + this suggestion pathologizes a normal response to distressing circumstances arising due to the pandemic.<br>Response: We have removed this statement from the discussion.<br>9.I recommend simplifying the results and even presenting graphs of the rating scales to visualize your findings. The tables are difficult to follow.<br>Response: We have revised the tables in response to previous comments, which have simplified the tables and we suggest that graphs may not add to the findings.<br>Reviewer #3: The authors assessed Children's interactions with non-household family members and child well-being during the COVID-19 pandemic. The authors used a number of data collection tools to meet their objective. Generally, this is an important study in the area of child protection and well-being. There are few issues that need clarification before the paper is published.<br>1.Under results section, I seem to miss data about the participant's age range (both children and their caregivers).<br>Response: We have added a Table that presents the parent and child's characteristics, which includes parents age range and children's Key Stage. We have also tried to make it clear in the tables whether the variables are referring to the parents or their children in response to your previous comment.<br>2.The choice of including parents/ caregivers as participants not the children themselves, should clearly come out.<br>Response: We have altered the wording throughout to make it clear that the study participants are parents and they are reporting about their perceptions of their children's behaviour.<br>Methods<br><br>1.There is a disconnection between the data collected, methods used and the main objective of the study. In line with the study's objectives, it appears to me that; the authors collected more data than what was required. For example, line 186, the authors collected data on: "Patients and public involvement. What did the authors do with data collected under "Statement about the lockdown" Line 179.<br>Response: We have changed the structure of the method and materials, which has made this section clearer, including that we did not collect any data about patients and the public, rather they were used to pilot our survey questions; and that the statements about the lockdown are reported in Tables 3 and 5.<br>2.Line 192: Analysis: recoding variables. This section is very confusing. The authors need to clearly follow the conventional lay out or common phrases used in manuscripts writing. A similar subheading on analysis appears on line 237.<br>Response: As stated in the previous comment, we have revised the layout of our materials and methods section.<br>3.Results: Before the authors point their audience to the tables for visualize the results, they should show some significant results in the texts. For example, results under subheading "risk factors" line 389 are missing.<br>Response: We have narratively reported the significant results before we present the full tabulated results. We have also added the adjusted odd ratios to the narrative results. Line 389 in the original manuscript refers to the discussion section that states: "attention and peer relationship problems [25]. Therefore, the risk of adverse mental...," we are unsure what section is missing. If this comment has not been resolved in our revised manuscript, we welcome further clarification on this comment.
**Additional Information:**	
**Question**	**Response**
**Financial Disclosure**<br><br>Enter a financial disclosure statement that describes the sources of funding for the work included in this submission. Review the submission guidelines for detailed requirements. View published research articles from *PLOS ONE* for specific examples.	Funding: This study was funded by the Economic and Social Research Council [grant number ES/P000703/1] and by the National Institute for Health and Care Research Health Protection Research Unit (NIHR HPRU) in Emergency Preparedness and Response [grant number NIHR200890], a partnership between the UK Health Security Agency, King's College London and the University of East Anglia. The views expressed are those of the author(s) and not necessarily those of the NIHR, UKHSA or the Department of Health and Social Care. For the purpose of open access, the author has applied a Creative Commons Attribution (CC BY) licence to any Author Accepted Manuscript version arising

This statement is required for submission and **will appear in the published article** if the submission is accepted. Please make sure it is accurate.<br><br>**Unfunded studies**<br>Enter: *The author(s) received no specific funding for this work.*<br><br>**Funded studies**<br>Enter a statement with the following details:<br>• Initials of the authors who received each award<br>• Grant numbers awarded to each author<br>• The full name of each funder<br>• URL of each funder website<br>• Did the sponsors or funders play any role in the study design, data collection and analysis, decision to publish, or preparation of the manuscript?<br>• **NO** - Include this sentence at the end of your statement: *The funders had no role in study design, data collection and analysis, decision to publish, or preparation of the manuscript.*<br>• **YES** - Specify the role(s) played.<br><br>* typeset	Role of the funding source: The funders had no role in study design, data collection and analysis, decision to publish, or preparation of the manuscript. The corresponding author had full access to all the data and had final responsibility for the decision to submit for publication.
**Competing Interests**<br><br>Use the instructions below to enter a competing interest statement for this submission. On behalf of all authors, disclose any competing interests that could be perceived to bias this work—acknowledging all financial support and any other relevant financial or non-financial competing interests.<br><br>This statement is required for submission and **will appear in the published article** if the submission is accepted. Please make sure it is accurate and that any funding sources listed in your Funding Information later in the submission form are also declared in your Financial Disclosure statement.<br><br>View published research articles from	I have read the journal's policy and the authors of this manuscript have the following competing interests: G.J.R., R.A. and L.S. participate in the UK's Scientific Advisory Group for Emergencies, or its subgroups. RA is an employee of the UK Health Security Agency. These groups did not fund the study or authors. The author interests do not alter our adherence to PLOS ONE policies on sharing data and materials.

Powered by Editorial Manager® and ProduXion Manager® from Aries Systems Corporation

PLOS ONE for specific examples.<br><br>**NO authors have competing interests**<br><br>Enter: *The authors have declared that no competing interests exist.*<br><br>**Authors with competing interests**<br><br>Enter competing interest details beginning with this statement:<br><br>*I have read the journal's policy and the authors of this manuscript have the following competing interests: [insert competing interests here]*<br><br>* typeset	
**Ethics Statement**<br><br>Enter an ethics statement for this submission. This statement is required if the study involved:<br><br>• Human participants<br>• Human specimens or tissue<br>• Vertebrate animals or cephalopods<br>• Vertebrate embryos or tissues<br>• Field research<br><br>Write "N/A" if the submission does not require an ethics statement.<br><br>General guidance is provided below. Consult the submission guidelines for detailed instructions. **Make sure that all information entered here is included in the Methods section of the manuscript.**	The research was approved by the Psychiatry, Nursing and Midwifery Research Ethics Subcommittee at King’s College London (LRS-19/20-18787).

Powered by Editorial Manager® and ProduXion Manager® from Aries Systems Corporation

**Format for specific study types**<br><br>**Human Subject Research (involving human participants and/or tissue)**<br>• Give the name of the institutional review board or ethics committee that approved the study<br>• Include the approval number and/or a statement indicating approval of this research<br>• Indicate the form of consent obtained (written/oral) or the reason that consent was not obtained (e.g. the data were analyzed anonymously)<br><br>**Animal Research (involving vertebrate animals, embryos or tissues)**<br>• Provide the name of the Institutional Animal Care and Use Committee (IACUC) or other relevant ethics board that reviewed the study protocol, and indicate whether they approved this research or granted a formal waiver of ethical approval<br>• Include an approval number if one was obtained<br>• If the study involved *non-human primates*, add *additional details* about animal welfare and steps taken to ameliorate suffering<br>• If anesthesia, euthanasia, or any kind of animal sacrifice is part of the study, include briefly which substances and/or methods were applied<br><br>**Field Research**<br><br>Include the following details if this study involves the collection of plant, animal, or other materials from a natural setting:<br>• Field permit number<br>• Name of the institution or relevant body that granted permission	
**Data Availability**<br><br>Authors are required to make all data underlying the findings described fully available, without restriction, and from the time of publication. PLOS allows rare exceptions to address legal and ethical concerns. See the PLOS Data Policy and FAQ for detailed information.	Yes - all data are fully available without restriction

Powered by Editorial Manager® and ProduXion Manager® from Aries Systems Corporation

A Data Availability Statement describing where the data can be found is required at submission. Your answers to this question constitute the Data Availability Statement and **will be published in the article**, if accepted.<br><br>**Important:** Stating 'data available on request from the author' is not sufficient. If your data are only available upon request, select 'No' for the first question and explain your exceptional situation in the text box.<br><br>Do the authors confirm that all data underlying the findings described in their manuscript are fully available without restriction?	
**Describe where the data may be found in full sentences. If you are copying our sample text, replace any instances of XXX with the appropriate details.**<br><br>• If the data are **held or will be held in a public repository**, include URLs, accession numbers or DOIs. If this information will only be available after acceptance, indicate this by ticking the box below. For example: *All XXX files are available from the XXX database (accession number(s) XXX, XXX.*).<br>• If the data are all contained **within the manuscript and/or Supporting Information files**, enter the following: *All relevant data are within the manuscript and its Supporting Information files.*<br>• If neither of these applies but you are able to provide **details of access elsewhere**, with or without limitations, please do so. For example:<br><br>*Data cannot be shared publicly because of [XXX]. Data are available from the XXX Institutional Data Access / Ethics Committee (contact via XXX) for researchers who meet the criteria for access to confidential data.*<br><br>*The data underlying the results presented in the study are available from (include the name of the third party*	The data set and survey questions related to this study can be accessed via the King's College London research data repository, KORDS (DOI 10.18742/21757232). For further information contact: kcl - research.data research.data@kcl.ac.uk.

Powered by Editorial Manager® and ProduXion Manager® from Aries Systems Corporation

and contact information or URL).<br>• This text is appropriate if the data are owned by a third party and authors do not have permission to share the data.<br><br>* typeset	
Additional data availability information:	Tick here if the URLs/accession numbers/DOIs will be available only after acceptance of the manuscript for publication so that we can ensure their inclusion before publication.

Lisa Woodland
King's College London
Cutcombe Road,
London
SE5 9RJ

Wednesday, 23 August 2023

Dear Dr Mduduzi Colani Shongwev,

We were offered to resubmit a revised manuscript about "Parent-reported child's close contact with non-household family members and their well-being during the COVID-19 pandemic: a cross-sectional survey" for consideration by your journal.

We want to thank all the reviewers for their comments and the time that they have taken to review our manuscript, following their suggestions the manuscript has been greatly improved. Reviews raised comments about our results tables being too large, we have addressed this issue and moved some of the information that was presented into the tables into the appendix. Similarly, it was suggested that we needed an additional table and narrative descriptions of the participants characteristics, which we have included. There were also comments about it not being clear that the study's participants were parents reporting about their children's behaviour, we have made this clearer by including that the results were 'parents perceptions' throughout the manuscript as suggested. There were a few comments about how we analysed the data, and we have provided a detailed rationale for the decisions that we made in response to these comments. We have included our response to each comment within our submission.

We confirm that this work is original and has not been published elsewhere, nor is it currently under consideration for publication elsewhere and will not be submitted to any other journal while under consideration by PLOS ONE.

In this paper, we show the risk factors associated with children having a low well-being during the COVID-19 school closures. We also show that worryingly, children were non-adherent to having physical interactions with non-household family members, as such increasing the risk of disease transmission in the community. The risks we found associated with children's low well-being and physical interactions with non-household family members are likely to exacerbate existing social and educational inequalities.

Understanding such factors are important if policy makers and schools are to provide effective measures that may mitigate these risks in future school closures.

Given the importance of schooling for a child's health we believe that this manuscript is appropriate for publication by PLOS ONE and will be of interest to your readers.

We have disclosed that G.J.R., R.A. and L.S. participate in the UK's Scientific Advisory Group for Emergencies, or its subgroups. These groups did not fund the

study or authors. RA is an employee of the UK Health Security Agency. The sponsors had no role in the design, execution, interpretation, or writing of the study. The corresponding author had full access to all the data and had final responsibility for the decision to submit for publication.

Please address all correspondence concerning this manuscript to me at lisa.woodland@kcl.ac.uk.

Thank you for your consideration of this manuscript.

Sincerely,

Lisa Woodland (on behalf of all authors)

Manuscript

Click here to access/download;Manuscript;Manuscript 23.08.23.docx

Parent-reported child's close contact with non-household family members and their well-being during the COVID-19 pandemic: a cross-sectional survey

Short title: Children's perceived close contact with non-household family members and their well-being during COVID-19

Lisa Woodland[1,5*], Louise E. Smith[1,5], Samantha K. Brooks[1,5], Rebecca K. Webster[2], Richard Amlôt[3,5], Antonia Rubin[4], G James Rubin[1,5]

[1] Department of Psychological Medicine, King's College London, London, United Kingdom

[2] Department of Psychology, University of Sheffield, Sheffield, United Kingdom

[3] Behavioural Science and Insights Unit, UK Health Security Agency, Salisbury, United Kingdom

[4] Trustee at Weald of Kent Grammar School, Tonbridge, Kent, United Kingdom

[5] NIHR Health Protection Research Unit in Emergency Preparedness and Response

* Corresponding author

E-mail: lisa.woodland@kcl.ac.uk (LW)

Abstract

In England (UK), at the start of the COVID-19 pandemic the public were required to reduce their physical contacts to slow the spread of COVID-19. We investigated risk factors for children having: 1) close contact with family members from outside their household ("non-adherent behaviour"); and 2) low well-being (using the Revised Child Anxiety and Depression Scale). We conducted an online cross-sectional survey, completed at any location of the participant's choice between 8 and 11 June 2020 in parents (n = 2,010) who were aged eighteen years or over and had a school-aged child (4-18 years old). Parents reported that 15% (n = 309) of children had non-adherent contact and that 26% (n = 519) had low well-being. We used a series of binary logistic regressions to investigate associations between outcomes and child and parent characteristics. Children had higher odds of having non-household contact when they had special educational needs [adjusted odds ratio, 2.19 (95% CI, 1.47 to 3.27)], lower well-being [2.65 (95% CI, 2.03 to 3.46)], were vulnerable to COVID-19 [2.17 (95% CI, 1.45 to 3.25)], lived with someone who was over 70 years old [2.56 (95% CI, 1.55 to 4.24)] and their parent had low well-being [1.94 (95% CI, 1.45 to 2.58)]. Children had higher odds of lower well-being when they had special educational needs [4.13 (95% CI, 2.90 to 5.87)], were vulnerable to COVID-19 [3.06 (95% CI, 2.15 to 4.36)], lived with someone else who was vulnerable to COVID-19 [2.08 (95% CI, 1.64 to 2.64)], or lived with someone who was over 70 years old [2.41 (95% CI, 1.51 to 3.83)]. Many children came into contact with non-household family members, mainly for childcare. Factors relating to COVID-19, children's well-being and education were also important. If school closures are needed in future, addressing these issues may help reduce contact.

Key Words: coronavirus disease 2019; child; adherence; wellbeing; social behaviour; pathogen transmission

Introduction

On 23 March 2020, England went into “lockdown,” with the public required to stay at home to slow the spread of COVID-19 [1]. The public were only allowed to leave home for a limited number of reasons, which included for medical attention, to buy necessities (as infrequently as possible), for exercise (once a day) and to work if absolutely necessary and they were unable to work from home. Physical distancing guidelines were implemented: people from different households were not allowed to meet and individuals were asked to stay two meters away from people not in their household. Non-essential shops were closed, and schools were closed to most children. Children could attend school if they were on a ‘health care plan’ due to a specific medical or social need or if their parent(s) were employed in jobs that were essential to the COVID-19 response (‘key workers’), such as doctors, teachers, and supermarket employees [2]. After two months of lockdown guidance, the restrictions started to ease. On 11 May it was announced that people could return to work, the restrictions on spending time outside were lifted, and individuals were allowed to arrange meetings with one person from another household as long as this happened outside and a two meter distance was maintained [3]. From 1 June 2020, the rules relaxed further: six people from different households were able to meet outside (though still at a two meter distance) and more children were eligible to attend school, including children in early years (four to five years), year one (five to six years) and year six (10 to 11 years) [4].

While all the restrictions caused strain within society, the closure of schools was particularly problematic. A systematic review suggested that between 18% and 60% of children scored above risk thresholds for distress, anxiety and depressive symptoms between February and July 2020 and increases in other adverse behaviours were also reported, such as an increase in suicidal ideation, worsening of mood, emotional difficulties and conduct problems in children [5]. In a qualitative study that we conducted in April 2020 we found that parent's and children's characteristics (e.g., parent's work status, children's age, access to resources for home-schooling and entertainment activities and space inside and outside the home to exercise) impacted how well families could cope with the COVID-19 school closures [6].

Determining, at the height of a crisis, whether the epidemiological benefits of school closures in terms of reducing disease transmission will exceed the psychological, physical, educational, and social costs is a daunting challenge for those who must make this decision. One question that must be factored into decision making is 'where do children go when their school is shut?'. In an ideal world (from an epidemiological perspective), parents would be able to ensure that children are placed under appropriate alternative supervision and remain apart from each other: continued mixing between children and other households would defeat the purpose of school closures. A systematic review of unplanned school closures prior to the COVID-19 pandemic found a 65% reduction in the mean total number of contacts for each student whilst schools were closed [7]. However, all 19 studies included in the review reported that children continued to meet with people from other households to some extent. A common reason for meeting others related to childcare. For example, one study found that children in households where special childcare arrangements

were needed during the closure had significantly higher odds of leaving home than children who were more independent and able to take care of themselves [8]. Parental concerns about the school closure also commonly reflected the difficulties they would face in trying to arrange childcare as well as concerns about lost education [7]. Previous research about children's adherence to medical treatment and encouraging healthy eating behaviours and physical exercise follow a similar pattern; factors relating to social networks and family cohesiveness or conflict [9, 10] and risk perceptions (e.g., perceived severity and vulnerability) [11, 12] are common in impacting adherence. These findings mirror a study that we conducted about family's adherence to England's COVID-19 guidelines. This study also found that parent's and children's characteristics, such as family income impacted adherence and that adherence may be more challenging for families with low psychological and physical ability [13].

In the context of the COVID-19 pandemic, issues around childcare while schools were closed were of particular concern because of a worry that grandparents might be called on to look after children in some families, putting older adults at increased risk of infection [14]. It was not just policy makers who worried about this, many children were themselves worried about their grandparents' vulnerability to COVID-19 [15] and of the risk that they themselves might infect their grandparents [16]. The extent to which these worries were borne out remains unclear.

Of course, while keeping children separate from each other and away from their grandparents might reduce disease transmission, it does present other risks. For example, a reduction in physical activity levels as a result of staying at home can affect physical and mental well-being [5]. While parents were advised to ensure their

children engaged in physical activity during the initial lockdown, the extent to which this occurred, and the impact of that initial period of isolation and inactivity is uncertain. Some parents and children may also have been very cautious about the risks associated with COVID-19, engaging in additional protective behaviours that were not explicitly recommended by the Government and which may have exerted an additional toll on well-being [17, 18].

In this study we investigated the risk factors associated with parents reporting children having: 1) close contact with family members from outside their household; and 2) low well-being during the first COVID-19 lockdown in the UK. Considering the evidence discussed above, we specifically explored whether close contact with non-household family members and low child well-being were associated with: parents' age, gender, and employment characteristics, children's age, gender and special educational needs status, and household's vulnerability to, perceptions about and behaviours in relation to COVID-19.

Materials and Methods

Design

We commissioned a market research company, BMG Research [19] to administer an online cross-sectional survey between 8 and 11 June 2020. Data collection occurred after lockdown restrictions had begun to be eased. At this stage in the pandemic, schools had re-opened to children in early years (four to five years), year one (five to six years) and year six (1 to 11 years). However, physical distancing restrictions remained in place throughout society, and while up to six non-household members could meet, this had to be outside and at a two-meter distance.

We have previously published data from this survey relating to parental perceptions of the hygiene procedures within schools [20] and investigating why some parents did not send their eligible children back to school [21].

Participants

Participants (n=2,447) were recruited from BMG Research’s panel. To achieve a sample that was broadly representative of the population, BMG Research monitored region, child age, child gender, parent/guardian age, and parent/guardian gender. Participants were eligible for the study if they were aged eighteen years or over, lived in England, and were a parent or guardian to a school-aged child (4-18 years old) who usually lived with them. One-hundred and eighty-three participants were screened out for non-eligibility by BMG, 226 participants dropped out after starting the survey and 28 completed but were removed for reasons related to quality control, such as completing the survey quickly compared to the average (14 minutes) and median (11 minutes) survey time or for ‘straight-lining’ (selecting the same option for every question) suggesting inattention to the questions. These issues are common in online surveys [22]. Two thousand and ten participants remained.

Quota sampling, as used in this study, is a non-probability based approach and we have therefore not reported the response rates as they are not helpful indicators of nonresponse bias. The response rates for each quota will differ, the denominator for each is essentially unknown, and the predominant source of bias is in the make-up of the underlying panels that are being recruited from, rather than whether the sample is representative of the panel [23].

The sample fell within five percentage points of the national population by the child's gender, key stage, and type of school attended against the known distribution for school children in England [24].

Participants were paid in points that could be accumulated by the participant and exchanged later for money. Our survey paid points equivalent to about £0.60.

The research was approved by the Psychiatry, Nursing and Midwifery Research Ethics Subcommittee at King's College London (LRS-19/20-18787). An information sheet was provided at the start of the survey, which described the purpose of the study. Participants were also informed of the study process, and they provided written consent before taking part.

Study Materials

The full survey is available in the supporting information (S1 Text).

All participants answered questions referring to their child who had the most recent birthday. In rare cases where children shared a birthday, we asked the parent to select one child.

Outcome one: Child's physical close contact with non-household family members

We asked parents to choose from a list of seven options about people who the child had come into close contact with in the past 24 hours ("someone [child] lives with;" "friends or other children who [child] does not live with"; "a babysitter, nanny or childminder;" "family member aged under 70 who [child] does not live with;" "family member aged over 70 who [child] does not live with;" "other children, not already reported above"; and "other adults, not already reported above"). We made

clear in the question that “by close contact we mean closer than 2 meters, for fifteen minutes or more,” which was the UK Government’s definition of close contact [25]. Parents were asked to report all the options that applied.

We created a binary variable to indicate whether the child had close contact with a family member from outside their household, defined as a “non-household close contact.” This included children reported as having had close contact with either “a family member aged under 70 who [child] does not live with” or “a family member aged over 70 who [child] does not live with.”

Outcome two: Child’s well-being

We asked parents to report the child’s well-being using two subscales from the Revised Child Anxiety and Depression Scale (RCADS): the generalised anxiety disorder (GAD) sub-scale and the major depressive disorder (MDD) sub-scale [26]. The GAD sub-scale asks parents to respond to six statements about their child (e.g., “my child worries about things”; and “my child worries that something awful will happen to someone in the family”). The MDD sub-scale asks parents to respond to ten statements about their child (e.g., “my child feels sad or empty”; “nothing is much fun for my child”; and “my child has trouble sleeping”). Parents can respond “never,” “sometimes,” “often,” and “always.”

We created a binary variable to indicate well-being in the child. The variable was recoded using SPSS syntax supplied by the RCADS authors, which assigns a value against each answer from 0 (“never”) to 3 (“always”) on the GAD and MDD RCADS sub-scales and creates a total score for each sub-scale [26]. The total score is turned into a t-score, normalising the RCADS scores within the population, by child’s age and gender. A t-score of 65 (approximately in the top 7% of un-referred

young people of the same age) on either the GAD or MDD sub-scale was our well-being cut off. “Lower well-being” represents a child with a medium or severe risk of clinical mental illness and “higher well-being” represents a child with low risk of clinical mental illness.

Predictor variables:

Parent and child personal characteristics

We asked parents to report their gender, age, region, household income, employment status, and if employed, whether they were working from home, level of education, marital status, ethnicity, and key worker status. We asked parents to report the child’s gender, age, school year, and whether the child had special educational needs. We also asked whether anyone within the household was aged over 70 years old or had a health condition that made them vulnerable to COVID-19 and whether they had access to outside space.

We recoded household income (less than £5,000; £5,000-£9,999; £10,000-£14,999; £15,000-£19,999; £20,000-£24,999; £25,000-£29,999; £30,000-£34,999; £35,000-£39,999; £40,000-£44,999; £45,000-£49,999; £50,000-£59,999; £60,000-£69,999; £70,000-£84,999; £85,000-£99,999; more than £100,000), employment status (full time paid job (31+ hours); part time paid job (<31 hours); doing paid work on a self-employed basis or within your own business; employed, but currently furloughed; student / on a government training programme (Nation Traineeship/Modern Apprenticeship); out of work (6 months or less); out of work (more than 6 months); looking after home / homemaker; retired; disabled OR long-term sick; unpaid work for a business, community or voluntary organisation), parent

education level (PhD/Doctor; Master’s; Bachelor’s Degree or equivalent (such as a NVQ level 5); higher education (such as a HND or a NVQ level 4); A-level or equivalent (such as Scottish Highers or NVQ level 3); GCSE and below (such as O level or an RSA Diploma); Other qualifications (Such as NVQ level 1); No qualifications), marital status (single (i.e. never married and never registered as a same sex civil-partnership); co-habiting with partner (but never married or been in a civil partnership); civil partnership; married; separated, but still legally married / in a civil partnership; divorced / civil partnership legally dissolved; widowed / surviving partner from a same-sex civil partnership), and ethnicity (English/Welsh/Scottish/Northern Irish/British; Irish; Gypsy or Irish Traveller; White and Black Caribbean; White and Black African; White and Asian; Indian; Pakistani; Bangladeshi; Chinese; Caribbean; African; Arab; any other (please specify)) into binary variables.

We recoded parent age, key worker status, and access to outside space into categorical variables, as shown in the results tables. We recoded child school year into Key Stages as used in the English education system (Early years = ages 4 to 5; Key Stage 1 = ages 5 to 7; Key Stage 2 = ages 7 to 11; Key Stage 3 = ages 11 to 14; Key Stage 4 = ages 14 to 16; Years 12 and 13 = ages 16 to 18). We created two binary variables to indicate whether the child, and someone in the household (other than the child) had a health condition that might make them particularly vulnerable to COVID-19. The responses “yourself [participant]” and “anyone else you live with” were combined into one variable to indicate that the child lived with someone vulnerable to COVID-19.

Parent’s well-being

We asked parents to report their well-being using the Patient-Health Questionnaire-4 (PHQ4) [27], which asks “over the last two weeks, how often have you been bothered by the following problems:” “feeling nervous, anxious or on the edge;” “not being able to stop or control worrying;” “little interest or pleasure in doing things;” and “feeling down, depressed, or hopeless.” Parents can respond “not at all,” “several days,” “more than half the days,” and “nearly every day.”

We created a binary variable to indicate low well-being in the parent. We assigned a value against each answer from 0 (“not at all”) to 3 (“nearly every day”) on the PHQ4 and summed responses (range 0 to 12). We used a cut off score of 5 or above to indicate low well-being in the parent, indicating moderate or severe risk of clinical anxiety or depression.

Child’s activities outside the home

We asked parents how many times the child had left the home in the past seven days: “to go to the shops for groceries, toiletries, or medicines;” “to go to the shops for other items;” “for exercise;” “for a medical need (e.g., an outpatient appointment);” “to go to school;” “to provide help to someone else;” “to meet friends;” to meet family members who they did not live with; and “for another reason.”

Items about the child’s activities outside the home were used as continuous variables.

Behaviours that parents and children had followed

We asked parents to report the behaviours that they or their children had followed in the past 7 days because of the risk of COVID-19 (e.g., “washed your hands thoroughly and regularly,” “stayed 2m (3 steps) away from people you do not

live with when outside your home,” “washed your clothes when you have returned home and “washed [child]’s clothes when she/he has returned home”). Parents could respond “yes” or “no” to each statement. Out of the 11 statements that we asked about, two were recommended and nine were not recommended by UK Government at the time of the study.

We created a continuous variable to indicate the COVID-19 behaviours that parents and their child had followed by combining all 11 responses to the statements about what the parent or child had done in the past seven days because of the risk of COVID-19.

Statements about lockdown

We included 16 statements about lockdown, which included questions about COVID-19 (e.g., “if [child] goes out, she/he is likely to catch coronavirus”), schooling (e.g., “[child] is keeping up with his/her schoolwork”) and home environment (e.g., “in the past 7 days, [child] has been bored”). Parents responded to each statement using a five-point Likert-scale from “strongly agree” to “strongly disagree,” or “not applicable.”

Items about lockdown were used as continues variables.

Missing data

We took the pragmatic approach to code the responses “don’t know”, “not applicable”, “prefer not to say” and “prefer to self-describe” as missing data. We adopted this approach for outcome variables because it was not possible to categorise participants as adherent or not, or as having good wellbeing or not. For predictors we adopted this approach to provide a clearer understanding of the

difference between endorsing, or not endorsing, each variable in terms of its impact on our outcomes.

Validity and Reliability

Previous studies have used RCADS [28], and the PHQ-4 [29], and they have both demonstrated good validity and reliability. The survey questions that related to COVID-19 were designed for this study based on our previous research [6], they have face validity and we copied the wording of official Government guidance where applicable. Five parents and one school trustee (see public involvement section) piloted the survey. Beyond this we do not have psychometric data about the validity and reliability of the questions.

Public involvement

A school trustee contributed to the development of the survey materials and co-authored this paper. The survey questions were reviewed by five parents of school children who also piloted the survey before publication. The feedback we received resulted in minor changes to the wording of some survey questions for clarity.

Analysis

We ran a series of binary logistic regressions using SPSS [30, 31] investigating the univariable associations between our two outcomes and each of our predictor variables. We ran a second set of binary logistic regressions controlling for child and parent characteristics (participant gender, age, region, household income, employment status, education level, marital status, ethnicity, and the child’s gender and school year).

We applied a Bonferroni correction to our results (p≤0.001) due to running many analyses. Only associations that met this level are discussed narratively in the results section.

Results

Most parents were: female (53%); between 36 and 45 years of age (43%); lived in London (17%); had a household income over £35,000 (55%); working (83%); highly educated (57%); married or co-habiting (84%); and of white ethnicity (87%) (Table 1).

Table 1: Parent, child and household characteristics (n = 2,010)

Variable	Level	n, (%)*
Parent gender	Male	931 (46)
	Female	1065 (53)
Parent age	18-35 years	394 (20)
	36-45 years	868 (43)
	46 years ≥	748 (37)
Region	East Midlands	151 (8)
	East of England	219 (11)
	North East	118 (6)
	North West	273 (14)
	South East	335 (17)
	South West	163 (8)
	West Midlands	211 (10)
	Yorkshire & the Humber	195 (10)
	London	345 (17)
Household income	≤ £34,999	804 (40)
	£35,000 ≥	1114 (55)
Employment status[1]	Working	1677 (83)
	Not working	321 (16)
Parent Working from home[2]	Yes	940 (59)
	No	628 (40)
Education level	≤ A-level	855 (43)
	Degree ≥	1139 (57)
Marital status	Living alone	327 (16)
	Married/cohabiting	1683 (84)
Ethnicity	White	1753 (87)
	BAME	241 (12)
Key worker status	Both parents	195 (10)
	One parent	847 (42)
	No	956 (48)
Child gender	Boy	1063 (53)
	Girl	947 (47)
Child school year	Early Years (ages 4 to 5)	101 (5)
	Key Stage 1 (ages 5 to 7)	356 (18)
	Key Stage 2 (ages 7 to 11)	675 (34)
	Key Stage 3 (ages 11 to 14)	395 (20)
	Key Stage 4 (ages 14 to 16)	328 (16)
	Years 12 & 13 (ages 16 to 18)	155 (8)
	Yes	161 (8)

Child had special educational needs	No	1832 (91)
Child lower well-being	Yes	519 (26)
	No	1491 (74)
Parent lower well-being	Yes	391 (19)
	No	1619 (81)
Child vulnerable COVID-19	Yes	157 (8)
	No	1826 (91)
Household vulnerable COVID-19	Yes	510 (25)
	No	1311 (65)
Someone over 70 years	Yes	87 (4)
	No	1923 (96)
COVID-19 behaviours followed by child and parent	Continuous (0=followed no behaviours, 11=followed all behaviours)	N=2,010, M=5.60, SD=3.10
Access to outside space[3]	Garden	1784 (89)
	Other[3]	157 (8)
	No	69 (3)

*Percentages may not total 100 due to rounding errors and missing data.
[1] Working includes students and volunteers
[2] Question only offered to participants who reported working in a paid job (full-time, part-time, and self-employed) and not to participants who reported being a student, on furlough and a volunteer.
[3] Participants that reported no access to a garden but had access to other outdoor spaces such as patio, terrace, and balcony
Abbreviations: n = number; % = percentage; M = mean; SD = standard deviation

Risk factors associated with children's non-household close contact

Of the 2010 parent responses, 15% (95% CI 14% to 17%, n=309) of children were perceived by the parents to have had close contact with a family member that they did not live with in the past 24 hours. This included 9% (n=189) who had close contact with a family member aged under 70 years and 6% (n=120) who had close contact with a family member aged 70 years or older, which includes n = 45 who had close contact with non-household family members under and over 70 years of age. ~~The parent and child characteristics associated with children who had non-household close contact are shown in Table 2 (see Table 7, Appendix A for the descriptives).~~ Parents with lower well-being had higher odds of reporting that their child had non-household close contact [adjusted odds ratio 1.94 (95% confidence interval (CI), 1.45 to 2.58)]. Parents who reported that their child had special educational needs, lower well-being, were vulnerable to COVID-19, and lived with someone who was over 70 years of age had higher odds of reporting that their child

had had non-household close contact [adjusted odd ratios, 2.19 (95% CI, 1.47 to 3.27); 2.65 (95% CI, 2.03 to 3.46); 2.56 (95% CI, 1.55 to 4.24), respectively].

Table 3 shows the associations between children who had non-household close contact, as perceived by their parents, and the predictor variables relating to parent perceptions about lockdown (see Table 8, Appendix B for the descriptives). Parents' agreement that their child had extra support at school before the closures, were upset about not seeing other family members that they did not live with, and that the parent had found it hard to keep up with work or other important commitments was associated with their children's non-household close contact [adjusted odd ratios, 0.82 (95% CI, 0.74 to 0.90); 0.84 (95% CI, 0.75 to 0.93); 0.78 (95% CI, 0.70 to 0.87), respectively].

The behaviours that families followed, perceived by parents because of the risk of COVID-19 are presented in Table 9 (see Appendix C).

Table 2 Binary logistic regression comparing parent and child characteristics and associations with children's non-household close contact

Variable	Level	Unadjusted odds Ratio (95% CI)	P-value	Adjusted odds ratio (95% CI) †	P-value
Parent gender	Male	0.88 (0.69 to 1.12)	0.30	0.97 (0.74 to 1.27)	0.80
	Female	Reference		Reference	
Parent age	18-35 years	**2.13** (1.54 to 2.95)**	**<0.001**	**1.28* (1.25 to 2.65)**	**0.002**
	36-45 years	1.29 (0.97 to 1.72)	0.09	1.17 (0.85 to 1.62)	0.34
	46 years ≥	Reference		Reference	
Region	East Midlands	0.73 (0.43 to 1.24)	0.25	0.69 (0.40 to 1.21)	0.19
	East of England	0.76 (0.48 to 1.21)	0.25	0.77 (0.47 to 1.26)	0.30
	North East	0.58 (0.31 to 1.08)	0.08	0.55 (0.28 to 1.06)	0.07
	North West	0.76 (0.50 to 1.17)	0.21	0.83 (0.52 to 1.32)	0.43
	South East	0.79 (0.53 to 1.18)	0.25	0.84 (0.55 to 1.28)	0.43
	South West	0.60 (0.35 to 1.03)	0.07	0.61 (0.35 to 1.08)	0.09
	West Midlands	0.66 (0.41 to 1.07)	0.09	0.63 (0.37 to 1.05)	0.08
	Yorkshire & the Humber	0.91 (0.58 to 1.44)	0.69	0.88 (0.54 to 1.44)	0.61
	London	Reference		Reference	
Household income	≤ £34,999	**1.34* (1.05 to 1.72)**	**0.02**	**1.44* (1.08 to 1.92)**	**0.01**
	£35,000 ≥	Reference		Reference	
Employment status[1]	Working	0.27 (1.21 to 0.86 to 1.72)	0.27	1.12 (0.77 to 1.63)	0.57
	Not working	Reference		Reference	
Parent Working from home[2]	Yes	**1.53* (1.15 to 2.05)**	**0.004**	^^^	^^^
	No	Reference		Reference	
Education level	≤ A-level	**0.77* (0.60 to 0.98)**	**0.04**	**0.70* (0.53 to 0.93)**	**0.01**
	Degree ≥	Reference		Reference	
Marital status	Living alone	**1.46* (1.08 to 1.98)**	**0.01**	1.36 (0.98 to 1.90)	0.07
	Married/cohabiting	Reference		Reference	
Ethnicity	White	0.97 (0.67 to 1.41)	0.88	1.25 (0.83 to 1.87)	0.28
	BAME	Reference		Reference	
Key worker status	Both parents	1.18 (0.76 to 1.84)	0.47	1.18 (0.74 to 1.90)	0.49
	One parent	**1.65** (1.28 to 2.14)**	**<0.001**	**1.53* (1.16 to 2.02)**	**0.002**
	No	Reference		Reference	
Child gender	Boy	1.14 (0.89 to 1.46)	0.29	1.14 (0.88 to 1.48)	1.14
	Girl	Reference		Reference	
Child school year	Early Years	1.53 (0.73 to 3.18)	0.26	1.22 (0.55 to 2.71)	0.63
	Key Stage 1	**2.24* (1.28 to 3.94)**	**0.01**	**2.11* (1.13 to 3.94)**	**0.02**
	Key Stage 2	1.40 (0.81 to 2.41)	0.23	1.28 (0.70 to 2.33)	0.42
	Key Stage 3	1.48 (0.84 to 2.63)	0.18	1.53 (0.83 to 2.83)	0.17
	Key Stage 4	1.10 (0.60 to 2.01)	0.77	1.21 (0.64 to 2.30)	0.56
	Years 12 & 13	Reference		Reference	
Child had special educational needs	Yes	**2.16** (1.49 to 3.14)**	**<0.001**	**2.19** (1.47 to 3.27)**	**<0.001**
	No	Reference		Reference	
Child lower well-being	Yes	**2.66** (2.07 to 3.42)**	**<0.001**	**2.65** (2.03 to 3.46)**	**<0.001**
	No	Reference		Reference	
Parent lower well-being	Yes	**2.15** (1.64 to 2.82)**	**<0.001**	**1.94** (1.45 to 2.58)**	**<0.001**
	No	Reference		Reference	
Child vulnerable COVID-19	Yes	**2.50** (1.73 to 3.61)**	**<0.001**	**2.17** (1.45 to 3.25)**	**<0.001**
	No	Reference		Reference	
Household vulnerable COVID-19	Yes	1.16 (0.87 to 1.54)	0.32	1.17 (0.87 to 1.59)	0.30
	No	Reference		Reference	

Someone over 70 years	Yes	**2.77** (1.74 to 4.42)**	**<0.001**	**2.56** (1.55 to 4.24)**	**<0.001**
	No	Reference		Reference	
COVID-19 behaviours followed by child and parent	Continuous (0=followed no behaviours, 11=followed all behaviours)	**1.06* (1.02 to 1.11)**	**0.002**	1.04 (0.99 to 1.08)	0.10
Access to outside space[3]	Garden	0.73 (0.40 to 1.38)	0.33	0.88 (0.44 to 1.75)	0.71
	Other[3]	1.28 (0.63 to 2.60)	0.49	1.25 (0.58 to 2.73)	0.71
	No	Reference		Reference	

* *p≤ ·05* and formatted bold

** *p≤ ·001* and formatted bold

Some results are rounded to 3 rather than 2 decimal places to distinguish between *p≤ ·05* and *p≤ ·001*

† When interpretating the predictor listed in the row, we controlled for the other variables listed here: participant gender, age, region, household income, employment status, education level, marital status, ethnicity, and the child's gender and school year

^^^ Not included variable highly correlated with employment variable

[1] Working includes students and volunteers

[2] Question only offered to participants who reported working in a paid job (full-time, part-time, and self-employed) and not to participants who reported being a student, on furlough and a volunteer.

[3] Participants that reported no access to a garden but had access to other outdoor spaces such as patio, terrace, and balcony

Abbreviations: CI = confidence interval

Table 3 Binary logistic regression comparing statements about lockdown and associations with children's non-household close contact. Close contact is defined by a child's close contact with a family member outside the household (n=309)

Statement	Level	Unadjusted odds ratio (95%, CI)	P-value	Adjusted odds ratio (95%, CI) †	P-value
If child goes out, she/he is likely to catch coronavirus	5-point Likert-scale (1=strongly agree, 5=strongly disagree)	**0.85* (0.75 to 0.95)**	**0.004**	0.89 (0.79 to 1.01)	0.07
If child goes out, she/he is likely to bring coronavirus back into our home	5-point Likert-scale (1=strongly agree, 5=strongly disagree)	**0.86* (0.77 to 0.96)**	**0.01**	0.91 (0.81 to 1.03)	0.13
Child is keeping up with his/her schoolwork	5-point Likert-scale (1=strongly agree, 5=strongly disagree)	**1.16* (1.04 to 1.29)**	**0.01**	**1.14* (1.02 to 1.27)**	**0.03**
I feel confident helping child with her/his schoolwork	5-point Likert-scale (1=strongly agree, 5=strongly disagree)	1.06 (0.94 to 1.19)	0.32	1.08 (0.96 to 1.23)	0.20
I feel supported by child's school	5-point Likert-scale (1=strongly agree, 5=strongly disagree)	1.00 (0.89 to 1.12)	0.96	0.99 (0.68 to 1.12)	0.87
I have access to all the resources that child needs to do her/his schoolwork	5-point Likert-scale (1=strongly agree, 5=strongly disagree)	1.05 (0.94 to 1.18)	0.38	1.09 (0.96 to 1.23)	0.18
During lockdown, child has learned about important things she/he wouldn't normally learn at school	5-point Likert-scale (1=strongly agree, 5=strongly disagree)	0.98 (0.87 to 1.11)	0.74	1.03 (0.30 to 1.17)	0.69
In the past 7 days, child has been bored	5-point Likert-scale (1=strongly agree, 5=strongly disagree)	**0.84** (0.75 to 0.93)**	**0.001**	**0.85* (0.76 to 0.95)**	**0.004**
In the past 7 days, my household has had a regular structure to the day	5-point Likert-scale (1=strongly agree, 5=strongly disagree)	1.12 (1.00 to 1.26)	0.05	1.09 (0.96 to 1.23)	0.18
In the past 7 days, child has kept in touch with her/his friends	5-point Likert-scale (1=strongly agree, 5=strongly disagree)	1.00 (0.90 to 1.12)	0.96	0.95 (0.84 to 1.07)	0.36
Child is worried about coronavirus	5-point Likert-scale (1=strongly agree, 5=strongly disagree)	0.98 (0.88 to 1.09)	0.72	0.99 (0.88 to 1.10)	0.82
In the past 7 days, child has felt upset about not seeing other family members who do not live with us	5-point Likert-scale (1=strongly agree, 5=strongly disagree)	**0.81** (0.73 to 0.89)**	**<0.001**	**0.84** (0.75 to 0.93)**	**0.001**
In the past 7 days, I have found it hard to keep up with work or other important commitments	5-point Likert-scale (1=strongly agree, 5=strongly disagree)	**0.75** (0.67 to 0.83)**	**<0.001**	**0.78** (0.70 to 0.87)**	**<0.001**
In the past 7 days, people in my household have been getting along well	5-point Likert-scale (1=strongly agree, 5=strongly disagree)	1.13 (1.00 to 1.28)	0.05	1.07 (0.94 to 1.22)	0.28
I am worried about the financial impact of lockdown measures	5-point Likert-scale (1=strongly agree, 5=strongly disagree)	0.92 (0.83 to 1.02)	0.13	0.93 (0.83 to 1.03)	0.18
Before the school closures, child had extra support at school	5-point Likert-scale (1=strongly agree, 5=strongly disagree)	**0.80** (0.73 to 0.88)**	**<0.001**	**0.82** (0.74 to 0.90)**	**<0.001**

* $p \leq \cdot 05$ and formatted bold

** $p \leq \cdot 001$ and formatted bold

Some results are rounded to 3 rather than 2 decimal places to distinguish between $p \leq \cdot 05$ and $p \leq \cdot 001$

† When interpretating the predictor listed in the row, we controlled for the other variables listed here: participant gender, age, region, household income, employment status, education level, marital status, ethnicity, and the child's gender and school year

Abbreviations: CI = confidence interval

Risk factors associated with children's lower well-being

Of the 2010 parent responses, 26% (95% CI 24% to 28%, n=519) reported that their child was perceived to have had low well-being. ~~The parent and child characteristics associated with child low well-being are shown in Table 4 (see Table 10, Appendix D for the descriptives). Parents aged between 18 and 35 years old,~~ who were a key worker and parents with lower well-being had higher odds of reporting that their child had lower well-being [adjusted odds ratio, ~~1.92 (95% CI, 1.40 to 2.64)~~; 1.51 (95% CI, 1.20 to 1.90); 7.26 (95% CI, 5.62 to 9.38), respectively]. Parents who reported that their child had had special educational needs, were vulnerable to COVID-19, lived with someone else who was also vulnerable to COVID-19, and lived with someone that was over 70 years old also had higher odds of reporting that their child had lower well-being [adjusted odd ratios, 4.13 (95% CI, 2.90 to 5.87)]; 3.06 (95% CI, 2.15 to 4.36); 2.08 (95% CI, 1.64 to 2.64)]; 2.41 (95% CI, 1.51 to 3.83), respectively]. Parents who reported that their household followed multiple precautionary behaviours because of the risk of COVID-19 had higher odds of reporting that their child had lower well-being [adjusted odds ratio, 1.11 (95% CI, 1.07 to 1.15)]. All lockdown statements bar two were associated with lower well-being (Table 5) (see Table 11, Appendix E for descriptives).

Table 4 Binary logistic regression comparing parent and child characteristics and associations with children's lower well-being (n=519)

Variable	Level	Unadjusted odds Ratio (95% CI)	P-value	Adjusted odds ratio (95% CI) †	P-value
Parent gender	Male	0.91 (0.74 to 1.11)	0.36	0.92 (0.74 to 1.15)	0.46
	Female	Reference		Reference	
Parent age	18-35 years	**1.67** (1.27 to 2.19)**	**<0.001**	**1.92** (1.40 to 2.64)**	**<0.001**
	36-45 years	1.11 (0.88 to 1.40)	0.38	1.25 (0.96 to 1.62)	0.09
	46 years ≥	Reference		Reference	
Region	East Midlands	1.27 (0.84 to 1.91)	0.26	1.21 (0.78 to 1.88)	0.39
	East of England	0.88 (0.60 to 1.29)	0.51	0.93 (0.61 to 1.39)	0.71
	North East	0.72 (0.44 to 1.18)	0.20	0.77 (0.46 to 1.30)	0.33
	North West	0.80 (0.56 to 1.15)	0.23	0.90 (0.61 to 1.33)	0.61
	South East	0.90 (0.64 to 1.26)	0.53	0.90 (0.62 to 1.29)	0.56
	South West	0.98 (0.64 to 1.48)	0.91	1.04 (0.67 to 1.63)	0.85
	West Midlands	0.79 (0.54 to 1.18)	0.25	0.75 (0.49 to 1.15)	0.19
	Yorkshire & the Humber	0.72 (0.48 to 1.09)	0.12	0.71 (0.46 to 1.11)	0.13
	London	Reference		Reference	
Household income	≤ £34,999	1.12 (0.91 to 1.37)	0.29	1.08 (0.85 to 1.37)	0.54
	£35,000 ≥	Reference		Reference	
Employment status[1]	Working	1.12 (0.85 to 1.48)	0.44	1.12 (0.82 to 1.52)	0.48
	Not working	Reference		Reference	
Parent Working from home[2]	Yes	1.16 (0.92 to 1.47)	0.20	^^^	^^^
	No	Reference		Reference	
Education level	≤ A-level	0.92 (0.75 to 1.12)	0.39	0.92 (0.74 to 1.16)	0.50
	Degree ≥	Reference		Reference	
Marital status	Living alone	1.17 (0.90 to 1.53)	0.24	1.15 (0.87 to 1.53)	0.33
	Married/cohabiting	Reference		Reference	
Ethnicity	White	0.93 (0.69 to 1.26)	0.65	1.06 (0.76 to 1.48)	0.73
	BAME	Reference		Reference	
Key worker status	Both parents	**1.70* (1.21 to 2.39)**	**0.002**	**1.71* (1.18 to 2.46)**	**0.004**
	One parent	**1.60** (1.29 to 1.98)**	**<0.001**	**1.51** (1.20 to 1.90)**	**<0.001**
	No	Reference		Reference	
Child gender	Boy	1.20 (0.98 to 1.47)	0.07	1.22 (0.98 to 1.50)	0.07
	Girl	Reference		Reference	
Child school year	Early Years	0.99 (0.55 to 1.79)	0.98	0.71 (0.38 to 1.32)	0.28
	Key Stage 1	1.13 (0.73 to 1.75)	0.59	0.80 (0.50 to 1.29)	0.37
	Key Stage 2	0.97 (0.65 to 1.47)	0.90	0.71 (0.46 to 1.10)	0.13
	Key Stage 3	1.22 (0.79 to 1.87)	0.37	1.03 (0.66 to 1.61)	1.61
	Key Stage 4	1.36 (0.88 to 2.11)	0.17	1.15 (0.73 to 1.80)	0.55
	Years 12 & 13	Reference		Reference	
Child has special educational needs	Yes	**4.24** (3.05 to 5.89)**	**<0.001**	**4.13** (2.90 to 5.87)**	**<0.001**
	No	Reference		Reference	
Parent low well-being	Yes	**6.77** (5.33 to 8.60)**	**<0.001**	**7.26** (5.62 to 9.38)**	**<0.001**
	No	Reference		Reference	
Child vulnerable COVID-19	Yes	**3.34** (2.40 to 4.65)**	**<0.001**	**3.06** (2.15 to 4.36)**	**<0.001**
	No	Reference		Reference	
Household vulnerable COVID-19	Yes	**2.01** (1.60 to 2.51)**	**<0.001**	**2.08** (1.64 to 2.64)**	**<0.001**
	No	Reference		Reference	
Someone over 70 years	Yes	**2.69** (1.75 to 4.16)**	**<0.001**	**2.41** (1.51 to 3.83)**	**<0.001**
	No	Reference		Reference	
COVID-19 behaviours followed by child and parent	Continuous (0=followed no behaviours,	**1.11** (1.07 to 1.14)**	**<0.001**	**1.11** (1.07 to 1.15)**	**<0.001**

	11=followed all behaviours)				
Access to outside space[3]	Garden	0.89 (0.52 to 1.53)	0.67	1.10 (0.60 to 2.03)	0.76
	Other[3]	1.19 (0.64 to 2.23)	0.58	1.36 (0.68 to 2.73)	0.38
	No	Reference		Reference	

* $p \leq \cdot 05$ and formatted bold

** $p \leq \cdot 001$ and formatted bold

Some results are rounded to 3 rather than 2 decimal places to distinguish between $p \leq \cdot 05$ and $p \leq \cdot 001$

† When interpretating the predictor listed in the row, we controlled for the other variables listed here: participant gender, age, region, household income, employment status, education level, marital status, ethnicity, and the child's gender and school year

^^^ Not included variable highly correlated with employment variable

[1] Working includes students and volunteers

[2] Question only offered to participants who reported working in a paid job (full-time, part-time, and self-employed) and not to participants who reported being a student, on furlough and a volunteer.

[3] Participants that reported no access to a garden but had access to other outdoor spaces such as patio, terrace, and balcony

Abbreviations: CI = confidence interval

Table 5 Binary logistic regression comparing statements about lockdown and associations with children's lower well-being (n=519)

Statement	Level	Unadjusted odds ratio (95%, CI)	P-value	Adjusted odds ratio (95%, CI) †	P-value
If child goes out, she/he is likely to catch coronavirus	5-point Likert-scale (1=strongly agree, 5=strongly disagree)	**0.63** (0.57 to 0.70)**	**<0.001**	**0.63** (0.57 to 0.70)**	**<0.001**
If child goes out, she/he is likely to bring coronavirus back into our home	5-point Likert-scale (1=strongly agree, 5=strongly disagree)	**0.63** (0.58 to 0.70)**	**<0.001**	**0.63** (0.57 to 0.70)**	**<0.001**
Child is keeping up with his/her schoolwork	5-point Likert-scale (1=strongly agree, 5=strongly disagree)	**1.43** (1.31 to 1.57)**	**<0.001**	**1.46** (1.33 to 1.60)**	**<0.001**
I feel confident helping child with her/his schoolwork	5-point Likert-scale (1=strongly agree, 5=strongly disagree)	**1.34** (1.22 to 1.48)**	**<0.001**	**1.34** (1.21 to 1.48)**	**<0.001**
I feel supported by child's school	5-point Likert-scale (1=strongly agree, 5=strongly disagree)	**1.26** (1.15 to 1.38)**	**<0.001**	**1.24** (1.12 to 1.37)**	**<0.001**
I have access to all the resources that child needs to do her/his schoolwork	5-point Likert-scale (1=strongly agree, 5=strongly disagree)	**1.30** (1.18 to 1.43)**	**<0.001**	**1.31** (1.18 to 1.44)**	**<0.001**
During lockdown, child has learned about important things she/he wouldn't normally learn at school	5-point Likert-scale (1=strongly agree, 5=strongly disagree)	**1.12* (1.01 to 1.24)**	**0.02**	**1.13* (1.02 to 1.25)**	**0.02**
In the past 7 days, child has been bored	5-point Likert-scale (1=strongly agree, 5=strongly disagree)	**0.68** (0.62 to 0.75)**	**<0.001**	**0.68** (0.61 to 0.75)**	**<0.001**
In the past 7 days, my household has had a regular structure to the day	5-point Likert-scale (1=strongly agree, 5=strongly disagree)	**1.23** (1.12 to 1.36)**	**<0.001**	**1.23** (1.11 to 1.36)**	**<0.001**
In the past 7 days, child has kept in touch with her/his friends	5-point Likert-scale (1=strongly agree, 5=strongly disagree)	**1.15* (1.05 to 1.25)**	**0.01**	1.67 (0.89 to 3.15)	0.11
Child is worried about coronavirus	5-point Likert-scale (1=strongly agree, 5=strongly disagree)	**0.60** (0.54 to 0.66)**	**<0.001**	**0.59** (0.53 to 0.65)**	**<0.001**
In the past 7 days, child has felt upset about not seeing other family members who do not live with us	5-point Likert-scale (1=strongly agree, 5=strongly disagree)	**0.64** (0.59 to 0.70)**	**<0.001**	**0.63** (0.57 to 0.69)**	**<0.001**
In the past 7 days, I have found it hard to keep up with work or other important commitments	5-point Likert-scale (1=strongly agree, 5=strongly disagree)	**0.56** (0.51 to 0.61)**	**<0.001**	**0.55** (0.49 to 0.60)**	**<0.001**
In the past 7 days, people in my household have been getting along well	5-point Likert-scale (1=strongly agree, 5=strongly disagree)	**1.47** (1.33 to 1.63)**	**<0.001**	**1.46** (1.31 to 1.63)**	**<0.001**
I am worried about the financial impact of lockdown measures	5-point Likert-scale (1=strongly agree, 5=strongly disagree)	**0.78** (0.71 to 0.85)**	**<0.001**	**0.77** (0.70 to 0.85)**	**<0.001**
Before the school closures, child had extra support at school	5-point Likert-scale (1=strongly agree, 5=strongly disagree)	**0.68** (0.63 to 0.74)**	**<0.001**	**0.69** (0.63 to 0.75)**	**<0.001**

* *p≤ ·05* and formatted bold

** *p≤ ·001* and formatted bold

Some results are rounded to 3 rather than 2 decimal places to distinguish between *p≤ ·05* and *p≤ ·001*

† When interpretating the predictor listed in the row, we controlled for the other variables listed here: participant gender, age, region, household income, employment status, education level, marital status, ethnicity, and the child's gender and school year

Abbreviations: CI = confidence interval

Associations between well-being and leaving home

Parents who perceived that their child had left the home for shopping, to provide help to someone else, to meet family, for medical treatment or for another reason had higher odds of reporting that their child had lower well-being (Table 6) [adjusted odds ratio, 1.24 (95% CI, 1.13 to 1.35); 1.37 (95% CI, 1.21 to 1.56); 1.19 (95% CI, 1.07 to 1.33); 3.70 (95% CI, 2.64 to 5.18); 1.22 (95% CI, 1.08 to 1.37), respectively]. Parents who perceived that their child was not leaving the home to exercise also had higher odds of reporting that their child had lower well-being [adjusted odds ratio, 0.93 (95% CI, 0.89 to 0.97)].

Table 6 Binary logistic regression comparing reasons for children leaving the home by children with low well-being (n=519)

Number of times that child had left the home for each reason on a continuous scale starting at 0	Child higher well-being, n, (%)	Child lower well-being, n, (%)	Unadjusted odds ratio (95%, CI)	P-value	Adjusted odds ratio (95%, CI) †	P-value
To go to the shops for groceries, toiletries, or medicines	N=1491, M=0.53, SD=1.03	N=519, M=0.83, SD=1.24	**1.25** (1.15 to 1.36)**	**<0.001**	**1.24** (1.13 to 1.35)**	**<0.001**
To go to the shops for other items	N=1491, M=0.38, SD=0.91	N=519, M=0.68, SD=1.13	**1.32** (1.20 to 1.45)**	**<0.001**	**1.31** (1.19 to 1.45)**	**<0.001**
To provide help to someone else	N=1491, M=0.15, SD=0.71	N=519, M=0.42, SD=0.99	**1.44** (1.27 to 1.64)**	**<0.001**	**1.37** (1.21 to 1.56)**	**<0.001**
To meet friends	N=1491, M=0.44, SD=1.51	N=519, M=0.64, SD=1.20	**1.14** (1.06 to 1.24)**	**<0.001**	**1.14* (1.05 to 1.24)**	**0.002**
To meet family members who don't live with you	N=1491, M=0.42, SD=0.88	N=519, M=0.59, SD=0.95	**1.22** (1.10 to 1.35)**	**<0.001**	**1.19** (1.07 to 1.33)**	**0.001**
For a medical need (e.g., an outpatient appointment)	N=1497, M=0.04, SD=0.25	N=519, M=0.24, SD=0.60	**3.75** (2.75 to 5.11)**	**<0.001**	**3.70** (2.64 to 5.18)**	**<0.001**
To go to school	N=1491, M=0.62, SD=1.48	N=519, M=0.77, SD=1.51	1.07 (1.00 to 1.14)	0.05	1.06 (0.99 to 1.13)	0.12
For exercise	N=1491, M=3.27, SD=2.99	N=519, M=2.64, SD=2.53	**0.92** (0.89 to 0.96)**	**<0.001**	**0.93** (0.89 to 0.97)**	**<0.001**
For another reason	N=1491, M=0.15, SD=0.76	N=519, M=0.33, SD=0.97	**1.25** (1.12 to 1.40)**	**<0.001**	**1.22** (1.08 to 1.37)**	**0.001**

* *p≤ ·05* and formatted bold
** *p≤ ·001* and formatted bold
Some results are rounded to 3 rather than 2 decimal places to distinguish between *p≤ ·05* and *p≤ ·001*
† When interpretating the predictor listed in the row, we controlled for the other variables listed here: participant gender, age, region, household income, employment status, education level, marital status, ethnicity, and the child's gender and school year

Abbreviations: n = number; M = mean; SD = standard deviation; % = percentage; CI = confidence interval

Discussion

During the COVID-19 pandemic, interventions were implemented to reduce physical contacts, including school closures and limits to physical contact between members of different households. We explored the impact of these measures to identify the risk factors for children having 1) close contact with family members from outside their household and 2) a low well-being.

Physical close contact with non-household family members

Our finding that 15% of children had close contact with non-household family members when schools were closed is concerning, as this would have increased the risk of disease transmission. This is particularly problematic for the 6% who had close contact with a non-household family member aged 70 years or over, who would be particularly at risk from COVID-19.

We found several variables associated with non-household contact that seem to point towards a high risk of such interactions among families that require childcare. This included, for example, when one parent was a key worker and when parents reported they had been unable to keep up with work or other important commitments. At the time of the survey parents were able to go back to work and some children were eligible to attend school. However, research suggests that most eligible children were still not attending school [21], and many will have needed alternative childcare.

We found that the risk for non-household family close contact was relatively equal across all children's ages, which contrasts with previous research [7]. We

suspect that the restrictions in place at the time of our survey explains this result. A longitudinal German study found that during COVID-19, the activities that children engaged in differed between ages [32]. However, these age differences disappeared when the guidance was more restrictive. That study also found that children commonly met elderly relatives (and friends) throughout the one-year study period, which supports our findings.

There appeared to be a cluster of predictive variables that indicated that children with worse psychological well-being had higher odds of meeting up with non-household family members. Variables in this group included children or their parent having low well-being and children being worried about non-household family members. We interpret these findings as the close contact may have been used to try to improve children's well-being [33], although as we are unable to determine causality; it could be that children's well-being reduced because of meeting their non-household family members.

Children who had extra support before the school closures and had special educational needs also had higher odds of non-household family close contact. This suggests that worries about education also increased risk of non-household close contact, which aligns with previous research [7].

Our results were less clear in terms of the perceived risk of COVID-19 and the impact on children's non-household family close contact. We did not find any associations between children's non-household family close contact and the lockdown statements that related to COVID-19. This finding is at odds with previous research [7]. In addition, children who were vulnerable to COVID-19 and lived with someone over 70 years old had higher odds of non-household family close contact.

These findings are concerning, suggesting a higher risk of contagion in these more vulnerable groups.

Children with a low well-being

Over a quarter (26%) of children reported on in our study had low well-being. A study in Switzerland conducted while schools were closed due to the COVID-19 pandemic found children's well-being and family functioning had reduced compared to before the pandemic, and the psychological impacts were greater for children at risk for neurodevelopmental impairments [34]. Children with special educational needs had four times higher odds of having a lower well-being compared to children without special educational needs. Previous research supports this finding [35]. In addition, a study suggests that children with special education needs are at risk for emotional, conduct, attention and peer relationship problems [36]. Therefore, the risk of adverse mental health problems could be exacerbated in children with special educational needs due to being at increased risk of challenging behaviour during lockdown. Parents who struggle to manage their children's behaviour are prone to using adverse parenting styles, and are at increased risk of family conflict and parental distress [37-39]. That said, 'special educational needs' covers a wide range of health and educational needs and research is needed to unpack what makes these children most at risk of poor well-being; increased risk could result from educational worries and a lack of academic support for these children [34].

We also observed risk factors for low well-being that relate to the home environment: parents who reported that their child had lower well-being also tended to report that they were bored or upset about not seeing family members, and that people in the household had not been getting along, or there was no structure to the

day. Parents also had higher odds of being worried about the financial impact of lockdown or unable to keep up with work and other commitments. Similar risk factors were identified in a study conducted during the COVID-19 pandemic that found individuals with poor sleep quality, increased distress due to financial circumstances, dependents, and or who were not adjusting to lockdown had higher odds of experiencing depression [40]. In addition, there is research showing the relationship between parent low well-being and child poor mental health outcomes [5, 37, 41]. We found that children were at seven times higher odds of lower well-being when the parent had low well-being. Our findings indicate that not only is parental well-being associated with child well-being but there also appears to be a link between parental distress due to home circumstances, such as financial worries.

Factors related to vulnerability to COVID-19 were also associated with children having lower well-being. These findings mirror research showing that risk factors for poor well-being in children include being worried about a grandparent's vulnerability to COVID-19, infecting their grandparents [15, 16] and being vulnerable to COVID-19 themselves [42, 43]. Children also had higher odds of a lower well-being if one of their parents was a key worker. Keyworkers commonly interacted with many people daily, which increased their risk of COVID-19 [44]. Families that adopted more protective behaviours because of the risk of COVID-19 also had higher odds of having a child with lower well-being; it is possible that increased levels of protective behaviours reflected a higher general sense of worry about the pandemic within the household.

More positively, children who had a higher well-being had higher odds of having parents who had confidence in home-schooling and who perceived that

children were keeping up with their schooling. Research has shown that parental self-efficacy can improve children’s well-being [45]. It is possible that this explains our results perhaps by reducing tension within the home about schoolwork. ~~We did not find any associations with parental education, income, or employment status in contrast to previous research [46] although we did note that younger parents had higher odds of reporting low well-being for their children than older parents. Younger adults in general experienced higher levels of stress and anxiety during the pandemic [40] because of more challenging working and living conditions, something which might account for our findings.~~

Exercise was also a protective factor; children had higher odds of having higher well-being the more times they had left the home to exercise. This finding aligns with previous research that shows the benefits of exercise on physical and mental health [40, 47-50]. We were surprised that access to outside space had no associations with either of our outcomes, although having access to outside space does not necessarily mean children use the space. To counterbalance the increased stress on families as a result of the pandemic, exercise should be promoted as a way to maintain well-being and parents should be taught how to increase their self-efficacy in managing difficult situations.

Limitations

We used a cross-sectional study design and the findings are based on a single point in time. Cross-sectional designs are common in health research as data can be gathered quickly so that the data can be used to respond rapidly to the health threat [51]. We used purposive sampling to meet pre-determined quotas for parent and children characteristics to broadly represent parents and children in England. The

use of quota rather than random sampling means that it is not possible to quantify the nature of any bias in the prevalence estimates that we have made. However, we have no reason to believe that the associations between the different variables that we measured would be affected by any theoretical bias [52]. Data were collected from self-reports, which can lead to self-report bias [53]. However, self-report data is a valid study design method [54, 55]. The RCADS is designed for children aged between 8 and 18 although parents reported on children from four years old [56]. However, we suggest this has minimal impact on our findings, RCADS has been found to be a reliable and valid measure in children as young as three years old [57].

Conclusions

During the COVID-19 pandemic we found that although children reduced their close contact, 15% had non-adherent physical contact with non-household family members. The reasons for close contact were largely related to a need for childcare, although factors relating to COVID-19, children's well-being and education were also important. Children who had special educational needs or had a parent with low well-being were most at risk for having a lower well-being themselves. Exercise and parent self-efficacy with home-schooling may help maintain children's mental and physical health.

~~**Keywords:** children; COVID-19; close contact; physical close contact; adherence; England; parents; well-being; mental health; physical health; exercise~~

Author contributions:

Lisa Woodland: Conceptualised, Methodology, Formal analysis, Investigation, Data Curation, Writing – Original Draft, Visualization, Project administration.

Louise E. Smith: Conceptualised, Methodology, Writing – Review & Editing.

Samantha K. Brooks: Methodology, Writing – Review & Editing.

Rebecca K. Webster: Conceptualised, Methodology, Writing – Review & Editing, Supervision, Funding acquisition.

Richard Amlôt: Conceptualised, Methodology, Writing – Review & Editing, Supervision, Funding acquisition.

Antonia Rubin: Investigation, Methodology, Writing – Review & Editing.

G. James Rubin: Conceptualised, Methodology, Resources, Writing – Review & Editing, Supervision, Funding acquisition.

Competing Interests: I have read the journal's policy and the authors of this manuscript have the following competing interests: G.J.R., R.A. and L.S. participate in the UK's Scientific Advisory Group for Emergencies, or its subgroups. RA is an employee of the UK Health Security Agency, which is an arm of the UK Government. This does not alter our adherence to PLOS ONE policies on sharing data and materials. These groups did not fund the study or authors.

Data Sharing: The data set and survey questions related to this study can be accessed via the King's College London research data repository, KORDS (DOI 10.18742/21757232). For further information contact: kcl - research.data research.data@kcl.ac.uk.

Appendix A

Table 7 Binary logistic regression comparing parent and child characteristics and associations with children's non-household close contact

Variable	Level	Child's not had close contact, n, (%)	Child's had close contact, n, (%)
Parent gender	Male	797 (86)	134 (14)
	Female	894 (84)	171 (16)
Parent age	18-35 years	305 (77)	89 (23)
	36-45 years	738 (85)	130 (15)
	46 years ≥	658 (88)	90 (12)
Region	East Midlands	129 (85)	22 (15)
	East of England	186 (85)	33 (15)
	North East	104 (88)	14 (12)
	North West	232 (85)	41 (15)
	South East	283 (84)	52 (16)
	South West	143 (88)	20 (12)

	West Midlands	183 (87)	28 (13)
	Yorkshire & the Humber	161 (83)	34 (17)
	London	280 (81)	65 (19)
Household income	≤ £34,999	660 (82)	144 (18)
	£35,000 ≥	958 (86)	156 (14)
Employment status[1]	Working	1412 (84)	265 (16)
	Not working	278 (87)	43 (13)
Parent Working from home[2]	Yes	774 (82)	166 (18)
	No	551 (88)	77 (12)
Education level	≤ A-level	740 (87)	115 (13)
	Degree ≥	947 (83)	192 (17)
Marital status	Living alone	262 (80)	65 (20)
	Married/cohabiting	1439 (86)	244 (14)
Ethnicity	White	1483 (85)	270 (15)
	BAME	203 (84)	38 (16)
Key worker status	Both parents	167 (86)	28 (14)
	One parent	686 (81)	161 (19)
	No	837 (88)	119 (12)
Child gender	Boy	891 (84)	172 (16)
	Girl	810 (86)	137 (14)
Child school year	Early Years	85 (84)	16 (16)
	Key Stage 1	279 (78)	77 (22)
	Key Stage 2	576 (85)	99 (15)
	Key Stage 3	334 (85)	61 (15)
	Key Stage 4	289 (88)	39 (12)
	Years 12 & 13	138 (89)	17 (11)
Child had special educational needs	Yes	118 (73)	43 (27)
	No	1568 (86)	264 (14)
Child lower well-being	Yes	384 (74)	135 (26)
	No	1317 (88)	174 (12)
Parent lower well-being	Yes	295 (75)	96 (25)
	No	1406 (87)	213 (13)
Child vulnerable COVID-19	Yes	111 (71)	46 (29)
	No	1566 (86)	260 (14)
Household vulnerable COVID-19	Yes	429 (84)	81 (16)
	No	1127 (86)	184 (14)
Someone over 70 years	Yes	59 (68)	28 (32)
	No	1642 (85)	281 (15)
COVID-19 behaviours followed by child and parent	Continuous (0=followed no behaviours, 11=followed all behaviours)	N=1701, M=5.51, SD=3.06	N=309, M=6.11, SD=3.27
Access to outside space[3]	Garden	1524 (85)	260 (15)
	Other[3]	121 (77)	36 (23)
	No	56 (81)	13 (19)

[1] Working includes students and volunteers
[2] Question only offered to participants who reported working in a paid job (full-time, part-time, and self-employed) and not to participants who reported being a student, on furlough and a volunteer.
[3] Participants that reported no access to a garden but had access to other outdoor spaces such as patio, terrace, and balcony
Abbreviations: n = number; % = percentage; M = mean; SD = standard deviation

Appendix B

Table 8 Binary logistic regression comparing statements about lockdown and associations with children's non-household close contact. Close contact is defined by a child's close contact with a family member outside the household (n=309)

Statement	Level	Child's not had close contact, n, (%)	Child's had close contact, n, (%)
If child goes out, she/he is likely to catch coronavirus	5-point Likert-scale (1=strongly agree, 5=strongly disagree)	N=1672, M=3.27, SD=1.03	N=309, M=3.08, SD=1.18
If child goes out, she/he is likely to bring coronavirus back into our home	5-point Likert-scale (1=strongly agree, 5=strongly disagree)	N=1672, M=3.23, SD=1.07	N=309, M=3.05, SD=1.16
Child is keeping up with his/her schoolwork	5-point Likert-scale (1=strongly agree, 5=strongly disagree)	N=1672, M=2.12, SD=1.08	N=309, M=2.30, SD=1.15
I feel confident helping child with her/his schoolwork	5-point Likert-scale (1=strongly agree, 5=strongly disagree)	N=1672, M=2.23, SD=1.03	N=305, M=2.30, SD=1.07
I feel supported by child's school	5-point Likert-scale (1=strongly agree, 5=strongly disagree)	N=1681, M=2.32, SD=1.07	N=306, M=2.32, SD=1.07
I have access to all the resources that child needs to do her/his schoolwork	5-point Likert-scale (1=strongly agree, 5=strongly disagree)	N=1678, M=2.15, SD=1.02	N=307, M=2.21, SD=1.03
During lockdown, child has learned about important things she/he wouldn't normally learn at school	5-point Likert-scale (1=strongly agree, 5=strongly disagree)	N=1691, M=2.40, SD=1.00	N=308, M=2.38, SD=1.05
In the past 7 days, child has been bored	5-point Likert-scale (1=strongly agree, 5=strongly disagree)	N=1694, M=2.54, SD=1.20	N=309, M=2.30, SD=1.16
In the past 7 days, my household has had a regular structure to the day	5-point Likert-scale (1=strongly agree, 5=strongly disagree)	N=1692, M=2.31, SD=1.02	N=309, M=2.43, SD=1.12
In the past 7 days, child has kept in touch with her/his friends	5-point Likert-scale (1=strongly agree, 5=strongly disagree)	N=1691, M=2.25, SD=1.14	N=307, M=2.26, SD=1.12
Child is worried about coronavirus	5-point Likert-scale (1=strongly agree, 5=strongly disagree)	N=1690, M=2.72, SD=1.12	N=306, M=2.70, SD=1.23
In the past 7 days, child has felt upset about not seeing other family members who do not live with us	5-point Likert-scale (1=strongly agree, 5=strongly disagree)	N=1684, M=2.97, SD=1.24	N=308, M=2.65, SD=1.25
In the past 7 days, I have found it hard to keep up with work or other important commitments	5-point Likert-scale (1=strongly agree, 5=strongly disagree)	N=1626, M=3.22, SD=1.21	N=299, M=2.79, SD=1.21
In the past 7 days, people in my household have been getting along well	5-point Likert-scale (1=strongly agree, 5=strongly disagree)	N=1691, M=2.08, SD=0.95	N=309, M=2.19, SD=1.01
I am worried about the financial impact of lockdown measures	5-point Likert-scale (1=strongly agree, 5=strongly disagree)	N=1685, M=2.51, SD=1.21	N=304, M=2.40, SD=1.16
Before the school closures, child had extra support at school	5-point Likert-scale (1=strongly agree, 5=strongly disagree)	N=1528, M=3.59, SD=1.36	N=285, M=3.16, SD=1.48

Abbreviations: n = number; % = percentage; M = mean; SD = standard deviation

Appendix C

Table 9 Frequencies to show the behaviours that parents or children had followed, in the past 7 days because of the risk of coronavirus. The findings our presented by children's non-household family close contact (n=309) and lower well-being (n=519).

Statements	Statement in relation to COVID-19 guidelines at the time	Total participants (n=2010, %)	Childs not had close contact (n=1701, %)	Childs had close contact (n=309, (%)	Child higher well-being, (n=1491, %)	Child lower well-being, (n=519, %)
Washed your hands thoroughly and regularly	Recommended	1895 (94%)	1617 (95)	278 (90)	1435 (96)	460 (89)
Stayed 2m (3 steps) away from people you do not live with when outside your home	Recommended	1867 (93%)	1599 (94)	268 (87)	1413 (95)	454 (87)
Cleaned or disinfected items you have brought into the home, such as groceries or parcels	Not recommended	1089 (54%)	908 (53)	181 (59)	779 (52)	310 (60)
Washed [CHILD]'s clothes when she/ he has returned home	Not recommended	1089 (54%)	829 (49)	176 (57)	688 (46)	317 (61)
Washed your clothes when you have returned home	Not recommended	959 (48%)	788 (46)	171 (55)	653 (44)	306 (59)
Used tissues or clothing to touch door handles or other objects when outside	Not recommended	954 (48%)	788 (46)	166 (54)	679 (46)	275 (53)
Covered your face or used a face mask when out and about	Not recommended	822 (41%)	678 (40)	144 (47)	571 (38)	251 (48)
Covered [CHILD's] face or given her/him a face mask when out and about	Not recommended	741 (37%)	600 (35)	141 (46)	500 (34)	241 (46)
Left items that you have brought into the home for 24 hours or more before using them, to kill any virus on them	Not recommended	725 (36%)	607 (36)	118 (38)	493 (33)	232 (45)
Worn protective gloves when out and about	Not recommended	693 (35%)	562 (33)	131 (43)	453 (30)	240 (46)
Made [CHILD] wear protective gloves when out and about	Not recommended	503 (25%)	390 (23)	113 (37)	308 (21)	195 (38)

Abbreviations: n = number of parents; % = percentage

Appendix D

Table 10 Binary logistic regression comparing parent and child characteristics and associations with children's lower well-being (n=519)

Variable	Level	Child higher well-being, n, (%)	Child lower well-being, n, (%)
Parent gender	Male	702 (75)	229 (25)

	Female	784 (74)	281 (26)
Parent age	18-35 years	263 (67)	131 (33)
	36-45 years	652 (75)	216 (25)
	46 years ≥	576 (77)	172 (23)
Region	East Midlands	101 (67)	50 (33)
	East of England	163 (74)	56 (26)
	North East	92 (78)	26 (22)
	North West	208 (76)	65 (24)
	South East	248 (74)	87 (26)
	South West	118 (72)	45 (28)
	West Midlands	161 (76)	50 (24)
	Yorkshire & the Humber	152 (78)	43 (22)
	London	248 (72)	97 (28)
Household income	≤ £34,999	583 (73)	221 (27)
	£35,000 ≥	832 (75)	282 (25)
Employment status[1]	Working	1240 (74)	437 (26)
	Not working	244 (76)	77 (24)
Parent Working from home[2]	Yes	682 (73)	258 (27)
	No	474 (75)	154 (25)
Education level	≤ A-level	642 (75)	213 (25)
	Degree ≥	836 (73)	303 (27)
Marital status	Living alone	234 (72)	93 (28)
	Married/cohabiting	1257 (75)	426 (25)
Ethnicity	White	1304 (74)	449 (26)
	BAME	176 (73)	65 (27)
Key worker status	Both parents	134 (69)	61 (31)
	One parent	593 (70)	254 (30)
	No	754 (79)	202 (21)
Child gender	Boy	771 (73)	292 (27)
	Girl	720 (76)	227 (24)
Child school year	Early Years	77 (76)	24 (24)
	Key Stage 1	263 (74)	93 (26)
	Key Stage 2	517 (7)	158 (23)
	Key Stage 3	286 (72)	109 (28)
	Key Stage 4	230 (70)	98 (30)
	Years 12 & 13	118 (76)	37 (24)
Child has special educational needs	Yes	71 (44)	90 (56)
	No	1410 (770	422 (23)
Parent low well-being	Yes	159 (41)	232 (59)
	No	1332 (82)	287 (18)
Child vulnerable COVID-19	Yes	77 (49)	80 (51)
	No	1393 (76)	433 (24)
Household vulnerable COVID-19	Yes	329 (65)	181 (35)
	No	1029 (78)	282 (22)
Someone over 70 years	Yes	46 (53)	41 (47)
	No	1445 (75)	478 (25)
COVID-19 behaviours followed by child and parent	Continuous (0=followed no behaviours, 11=followed all behaviours)	N=1491, M=5.35, SD=3.02	N=519, M=6.32, SD=3.22
Access to outside space[3]	Garden	1333 (75)	451 (25)
	Other[3]	108 (69)	49 (31)
	No	50 (72)	19 (28)

* *p≤ ·05* and formatted bold
** *p≤ ·001* and formatted bold
Some results are rounded to 3 rather than 2 decimal places to distinguish between *p≤ ·05* and *p≤ ·001*
† When interpretating the predictor listed in the row, we controlled for the other variables listed here: participant gender, age, region, household income, employment status, education level, marital status, ethnicity, and the child's gender and school year
^^^ Not included variable highly correlated with employment variable

[1] Working includes students and volunteers
[2] Question only offered to participants who reported working in a paid job (full-time, part-time, and self-employed) and not to participants who reported being a student, on furlough and a volunteer.
[3] Participants that reported no access to a garden but had access to other outdoor spaces such as patio, terrace, and balcony
Abbreviations: n = number of parents; % = percentage; CI = confidence interval

Appendix E

Table 11 Binary logistic regression comparing statements about lockdown and associations with children's lower well-being (n=519)

Statement	Level	Child higher well-being, n, (%)	Child lower well-being, n, (%)
If child goes out, she/he is likely to catch coronavirus	5-point Likert-scale (1=strongly agree, 5=strongly disagree)	N=1473, M=3.37, SD=1.02	N=515, M=2.87, SD=1.06
If child goes out, she/he is likely to bring coronavirus back into our home	5-point Likert-scale (1=strongly agree, 5=strongly disagree)	N=1465, M=3.33, SD=1.04	N=515, M=2.82 SD=1.11
Child is keeping up with his/her schoolwork	5-point Likert-scale (1=strongly agree, 5=strongly disagree)	N=1470, M=2.03, SD=1.01	N=508, M=2.48, SD=1.24
I feel confident helping child with her/his schoolwork	5-point Likert-scale (1=strongly agree, 5=strongly disagree)	N=1469, M=2.16, SD=0.98	N=508, M=2.48, SD=1.13
I feel supported by child's school	5-point Likert-scale (1=strongly agree, 5=strongly disagree)	N=1475, M=2.25, SD=1.03	N=512, M=2.52 SD=1.15
I have access to all the resources that child needs to do her/his schoolwork	5-point Likert-scale (1=strongly agree, 5=strongly disagree)	N=1473, M=2.09, SD=0.97	N=512, M=2.37, SD=1.14
During lockdown, child has learned about important things she/he wouldn't normally learn at school	5-point Likert-scale (1=strongly agree, 5=strongly disagree)	N=1481, M=2.37, SD=0.98	N=518, M=2.49, SD=1.08
In the past 7 days, child has been bored	5-point Likert-scale (1=strongly agree, 5=strongly disagree)	N=1486, M=2.63, SD=1.20	N=517, M=2.14, SD=1.11
In the past 7 days, my household has had a regular structure to the day	5-point Likert-scale (1=strongly agree, 5=strongly disagree)	N=1484, M=2.27, SD=0.99	N=517, M=2.50, SD=1.14
In the past 7 days, child has kept in touch with her/his friends	5-point Likert-scale (1=strongly agree, 5=strongly disagree)	N=1483, M=2.21, SD=1.12	N=515, M=2.39, SD=1.19
Child is worried about coronavirus	5-point Likert-scale (1=strongly agree, 5=strongly disagree)	N=1481, M=2.88, SD=1.12	N=515, M=2.27, SD=1.08
In the past 7 days, child has felt upset about not seeing other family members who do not live with us	5-point Likert-scale (1=strongly agree, 5=strongly disagree)	N=1475, M=3.09, SD=1.24	N=517, M=2.45, SD=1.15
In the past 7 days, I have found it hard to keep up with work or other important commitments	5-point Likert-scale (1=strongly agree, 5=strongly disagree)	N=1429, M=3.37, SD=1.19	N=496, M=2.55, SD=1.15
In the past 7 days, people in my household have been getting along well	5-point Likert-scale (1=strongly agree, 5=strongly disagree)	N=1484, M=2.00, SD=0.93	N=516, M=2.37, SD=0.97

I am worried about the financial impact of lockdown measures	5-point Likert-scale (1=strongly agree, 5=strongly disagree)	N=1475, M=2.58, SD=1.23	N=514, M=2.24, SD=1.10
Before the school closures, child had extra support at school	5-point Likert-scale (1=strongly agree, 5=strongly disagree)	N=1326, M=3.72, SD=1.32	N=487, M=2.98, SD=1.42

Abbreviations: N = number of parents; M = mean; SD = standard deviation; % = percentage

References

1. HM Government Prime Minister's statement on coronavirus (COVID-19): 23 March 2020. https://www.gov.uk/government/speeches/pm-address-to-the-nation-on-coronavirus-23-march-2020 (accessed 11 June 2020).

2. HM Government Children of critical workers and vulnerable children who can access schools or educational settings. https://www.gov.uk/government/publications/coronavirus-covid-19-maintaining-educational-provision/guidance-for-schools-colleges-and-local-authorities-on-maintaining-educational-provision (accessed 16 March 2021).

3. HM Government Prime Minister's statement on coronavirus (COVID-19): 11 May 2020. https://www.gov.uk/government/speeches/pm-statement-on-coronavirus-11-may-2020 (accessed 22 March 2022).

4. HM Government, PM: Six people can meet outside under new measures to ease lockdown. 2020.

5. Viner, R.; Russell, S.; Saulle, R.; Croker, H.; Stansfield, C.; Packer, J.; Nicholls, D.; Goddings, A.-L.; Bonell, C.; Hudson, L.; Hope, S.; Ward, J.; Schwalbe, N.; Morgan, A.; Minozzi, S., School Closures During Social Lockdown and Mental Health, Health Behaviors, and Well-being Among Children and Adolescents During the First COVID-19 Wave. *JAMA Pediatrics* **2022**.

6. Woodland, L.; Hodson, A.; Webster, R. K.; Amlôt, R.; Smith, L. E.; Rubin, G. J., A qualitative study about how families coped with managing their well-being, children’s physical activity and education during the COVID-19 school closures in England. *PLOS ONE* **2022,** *17* (12), e0279355.

7. Brooks, S. K.; Smith, L. E.; Webster, R. K.; Weston, D.; Woodland, L.; Hall, I.; Rubin, G. J., The impact of unplanned school closure on children's social contact: rapid evidence review. *Euro Surveill* **2020,** *25* (13).

8. Mizumoto, K.; Yamamoto, T.; Nishiura, H., Contact behaviour of children and parental employment behaviour during school closures against the pandemic influenza A (H1N1-2009) in Japan. *Journal of International Medical Research* **2013,** *41* (3), 716-724.

9. DiMatteo, M. R., The role of effective communication with children and their families in fostering adherence to pediatric regimens. *Patient Education and Counseling* **2004,** *55* (3), 339-344.

10. Kunin-Batson, A. S.; Seburg, E. M.; Crain, A. L.; Jaka, M. M.; Langer, S. L.; Levy, R. L.; Sherwood, N. E., Household factors, family behavior patterns, and adherence to dietary and physical activity guidelines among children at risk for obesity. *Journal of nutrition education and behavior* **2015,** *47* (3), 206-215. e1.

11. Smith, L. E.; D'Antoni, D.; Jain, V.; Pearce, J. M.; Weinman, J.; Rubin, G. J., A systematic review of factors affecting intended and actual adherence with antiviral medication as treatment or prophylaxis in seasonal and pandemic flu. *Influenza and other respiratory viruses* **2016,** *10* (6), 462-478.

12. Gentili, D.; Bardin, A.; Ros, E.; Piovesan, C.; Ramigni, M.; Dalmanzio, M.; Dettori, M.; Filia, A.; Cinquetti, S., Impact of Communication Measures Implemented During a School Tuberculosis Outbreak on Risk Perception among Parents and School Staff, Italy, 2019. *International Journal of Environmental Research and Public Health* **2020,** *17* (3), 911.

13. Woodland, L.; Hodson, A.; Webster, R. K.; Amlôt, R.; Smith, L. E.; Rubin, G. J., A Qualitative Study Evaluating the Factors Affecting Families' Adherence to the First COVID-19 Lockdown in England Using the COM-B Model and TDF. *International Journal of Environmental Research and Public Health* **2022,** *19* (12), 7305.

14. HM Government *Sixteenth SAGE meeting on Wuhan Coronavirus (Covid-19), 16th March 2020*; 2020.

15. Sarkadi, A.; Sahlin Torp, L.; Pérez-Aronsson, A.; Warner, G., Children's expressions of worry during the COVID-19 pandemic in Sweden. *Journal of Pediatric Psychology* **2021,** *46* (8), 939-949.

16. Idoiaga, N.; Berasategi, N.; Eiguren, A.; Picaza, M., Exploring children's social and emotional representations of the Covid-19 pandemic. *Frontiers in Psychology* **2020,** *11*, 1952.

17. Denford, S.; Morton, K. S.; Lambert, H.; Zhang, J.; Smith, L. E.; Rubin, G. J.; Cai, S.; Zhang, T.; Robin, C.; Lasseter, G.; Hickman, M.; Oliver, I.; Yardley, L., Understanding patterns of adherence to COVID-19 mitigation measures: a qualitative interview study. *Journal of Public Health* **2021,** *43* (3), 508-516.

18. Lasseter, G.; Compston, P.; Robin, C.; Lambert, H.; Hickman, M.; Denford, S.; Reynolds, R.; Zhang, J.; Cai, S.; Zhang, T.; Smith, L. E.; Rubin, J.; Yardley, L.; Amlot, R.; Oliver, I., Exploring the impact of shielding advice on the health and wellbeing of individuals identified as extremely vulnerable and advised to shield in Southwest England amid the COVID-19 pandemic: A mixed-methods evaluation. Cold Spring Harbor Laboratory: 2022.

19. BMG Research BMG About Us. https://www.bmgresearch.co.uk/bmg/about-us/ (accessed 1 September 2022).

20. Smith, L. E.; Woodland, L.; Amlôt, R.; Rubin, A.; Rubin, G. J., A cross-sectional survey of parental perceptions of COVID-19 related hygiene measures within schools and adherence to social distancing in journeys to and from school. *BMJ Paediatrics Open* **2020,** *4* (1).

21. Woodland, L.; Smith, L. E.; Webster, R. K.; Amlôt, R.; Rubin, A.; Wessely, S.; Rubin, G. J., Why did some parents not send their children back to school following school closures during the COVID-19 pandemic: a cross-sectional survey. *BMJ Paediatrics Open* **2021,** *5* (1), e001014.

22. Zhang, C.; Conrad, F. In *Speeding in web surveys: The tendency to answer very fast and its association with straightlining*, Survey research methods, 2014; pp 127-135.

23. Callegaro, M.; DiSogra, C., Computing Response Metrics for Online Panels. *Public Opinion Quarterly* **2008,** *72* (5), 1008-1032.

24. HM Government Schools, pupils and their characteristics: Academic Year 2019/20. https://explore-education-statistics.service.gov.uk/find-statistics/school-pupils-and-their-characteristics (accessed 25 June 2020).

25. HM Government [Withdrawn] Guidance for contacts of people with confirmed coronavirus (COVID-19) infection who do not live with the person. https://www.gov.uk/government/publications/guidance-for-contacts-of-people-with-possible-or-confirmed-coronavirus-covid-19-infection-who-do-not-live-with-the-person/guidance-for-contacts-of-people-with-possible-or-confirmed-coronavirus-covid-19-infection-who-do-not-live-with-the-person (accessed 16 August 2023).

26. Chorpita, B. F.; Yim, L.; Moffitt, C.; Umemoto, L. A.; Francis, S. E., Assessment of symptoms of DSM-IV anxiety and depression in children: a revised child anxiety and depression scale. *Behaviour Research and Therapy* **2000,** *38* (8), 835-855.

27. Kroenke, K.; Spitzer, R. L.; Williams, J. B. W.; Lowe, B., An Ultra-Brief Screening Scale for Anxiety and Depression: The PHQ-4. *Psychosomatics* **2009,** *50* (6), 613-621.

28. Piqueras, J. A.; Martín-Vivar, M.; Sandin, B.; San Luis, C.; Pineda, D., The Revised Child Anxiety and Depression Scale: A systematic review and reliability generalization meta-analysis. *Journal of affective disorders* **2017,** *218*, 153-169.

29. Wicke, F. S.; Krakau, L.; Löwe, B.; Beutel, M. E.; Brähler, E., Update of the standardization of the Patient Health Questionnaire-4 (PHQ-4) in the general population. *Journal of Affective Disorders* **2022,** *312*, 310-314.

30. IBM Corp IBM SPSS Statistics for Windows, 26.0 Armonk, NY.

31. IBM Corp IBM SPSS Statistics for Windows, 28.0 Armonk, NY.

32. Paulsen, M.; Zychlinsky Scharff, A.; De Cassan, K.; Sugianto, R. I.; Blume, C.; Blume, H.; Christmann, M.; Hauß, C.; Illig, T.; Jonczyk, R.; Klopp, N.; Kopfnagel, V.; Lichtinghagen, R.; Lucas, H.; Luhr, A.; Mutschler, F.; Pietschmann,

T.; Pott, P.-C.; Prokein, J.; Schaefer, P.; Stahl, F.; Stanislawski, N.; Von Der Born, J.; Schmidt, B. M. W.; Heiden, S.; Stiesch, M.; Memaran, N.; Melk, A., Children and Adolescents’ Behavioral Patterns in Response to Escalating COVID-19 Restriction Reveal Sex and Age Differences. *Journal of Adolescent Health* **2022,** *70* (3), 378-386.

33. Morina, N.; Kip, A.; Hoppen, T. H.; Priebe, S.; Meyer, T., Potential impact of physical distancing on physical and mental health: a rapid narrative umbrella review of meta-analyses on the link between social connection and health. *BMJ Open* **2021,** *11* (3), e042335.

34. Ehrler, M.; Werninger, I.; Schnider, B.; Eichelberger, D. A.; Naef, N.; Disselhoff, V.; Kretschmar, O.; Hagmann, C. F.; Latal, B.; Wehrle, F. M., Impact of the COVID-19 pandemic on children with and without risk for neurodevelopmental impairments. *Acta Paediatrica* **2021,** *110* (4), 1281-1288.

35. Essex, M. J.; Kraemer, H. C.; Armstrong, J. M.; Boyce, W. T.; Goldsmith, H. H.; Klein, M. H.; Woodward, H.; Kupfer, D. J., Exploring Risk Factors for the Emergence of Children's Mental Health Problems. *Archives of General Psychiatry* **2006,** *63* (11), 1246.

36. Deighton, J.; Lereya, S. T.; Casey, P.; Patalay, P.; Humphrey, N.; Wolpert, M., Prevalence of mental health problems in schools: poverty and other risk factors among 28 000 adolescents in England. *British Journal of Psychiatry* **2019,** *215* (3), 565-567.

37. Wille, N.; Bettge, S.; Ravens-Sieberer, U., Risk and protective factors for children’s and adolescents’ mental health: results of the BELLA study. *European Child & Adolescent Psychiatry* **2008,** *17* (S1), 133-147.

38. Prime, H.; Wade, M.; Browne, D. T., Risk and resilience in family well-being during the COVID-19 pandemic. *American Psychologist* **2020,** *75* (5), 631.

39. Spinelli, M.; Lionetti, F.; Pastore, M.; Fasolo, M., Parents' stress and children's psychological problems in families facing the COVID-19 outbreak in Italy. *Frontiers in psychology* **2020,** *11*, 1713.

40. Varma, P.; Junge, M.; Meaklim, H.; Jackson, M. L., Younger people are more vulnerable to stress, anxiety and depression during COVID-19 pandemic: A global cross-sectional survey. *Progress in Neuro-Psychopharmacology and Biological Psychiatry* **2021,** *109*, 110236.

41. Davis, E.; Sawyer, M. G.; Lo, S. K.; Priest, N.; Wake, M., Socioeconomic risk factors for mental health problems in 4–5-year-old children: Australian population study. *Academic Pediatrics* **2010,** *10* (1), 41-47.

42. Tunçgenç, B.; Newson, M.; Sulik, J.; Zhao, Y.; Dezecache, G.; Deroy, O.; Zein, M. E., Social alignment matters: Following pandemic guidelines is associated with better wellbeing. *BMC Public Health* **2022,** *22* (1).

43. Saurabh, K.; Ranjan, S., Compliance and Psychological Impact of Quarantine in Children and Adolescents due to Covid-19 Pandemic. *The Indian Journal of Pediatrics* **2020,** *87* (7), 532-536.

44. Environmental Modelling Group (EMG) Transmission Group *COVID-19 Risk by Occupation and Workplace*; HM Government: 11 February, 2021.

45. Albanese, A. M.; Russo, G. R.; Geller, P. A., The role of parental self-efficacy in parent and child well-being: A systematic review of associated outcomes. *Child: care, health and development* **2019,** *45* (3), 333-363.

46. Reiss, F., Socioeconomic inequalities and mental health problems in children and adolescents: A systematic review. *Social Science & Medicine* **2013,** *90*, 24-31.

47. Biddle, S. J. H.; Ciaccioni, S.; Thomas, G.; Vergeer, I., Physical activity and mental health in children and adolescents: An updated review of reviews and an analysis of causality. *Psychology of Sport and Exercise* **2019,** *42*, 146-155.

48. Ahn, S.; Fedewa, A. L., A Meta-analysis of the Relationship Between Children's Physical Activity and Mental Health. *Journal of Pediatric Psychology* **2011,** *36* (4), 385-397.

49. Eddolls, W. T. B.; McNarry, M. A.; Lester, L.; Winn, C. O. N.; Stratton, G.; Mackintosh, K. A., The association between physical activity, fitness and body mass index on mental well-being and quality of life in adolescents. *Quality of Life Research* **2018,** *27* (9), 2313-2320.

50. Benzing, V.; Gaillard, P.; Scheidegger, D.; Dössegger, A.; Nigg, C. R.; Schmidt, M., COVID-19: Physical Activity and Quality of Life in a Sample of Swiss School Children during and after the First Stay-at-Home. *International Journal of Environmental Research and Public Health* **2022,** *19* (4), 2231.

51. Wang, X.; Cheng, Z., Cross-sectional studies: strengths, weaknesses, and recommendations. *Chest* **2020,** *158* (1), S65-S71.

52. Kohler U Possible Uses of Nonprobability Sampling for the Social Sciences. *Survey Methods: Insights from the Field*. https://surveyinsights.org/?p=10981 (accessed 29 July 2020).

53. Epstein, W. M., Response bias in opinion polls and American social welfare. *The Social Science Journal* **2006,** *43* (1), 99-110.

54. Kormos, C.; Gifford, R., The validity of self-report measures of proenvironmental behavior: A meta-analytic review. *Journal of Environmental Psychology* **2014,** *40*, 359-371.

55. Craig, K.; Hale, D.; Grainger, C.; Stewart, M. E., Evaluating metacognitive self-reports: systematic reviews of the value of self-report in metacognitive research. *Metacognition and Learning* **2020,** *15*, 155-213.

56. Ebesutani, C.; Chorpita, B. F.; Higa-Mcmillan, C. K.; Nakamura, B. J.; Regan, J.; Lynch, R. E., A Psychometric Analysis of the Revised Child Anxiety and

Depression Scales—Parent Version in a School Sample. *Journal of Abnormal Child Psychology* **2011,** *39* (2), 173-185.

57. Ebesutani, C.; Tottenham, N.; Chorpita, B., The Revised Child Anxiety and Depression Scale - Parent Version: Extended Applicability and Validity for Use with Younger Youth and Children with Histories of Early-Life Caregiver Neglect. *Journal of Psychopathology and Behavioral Assessment* **2015,** *37* (4), 705-718.

Supporting information:

S1 Text. **Full survey material.** The information can be downloaded at: DOI 10.18742/21757232

Click here to access/download
Supporting Information
S.1 Text.docx

Ethics approval (for editors only)

Click here to access/download

Other

Ethics LRS-18787.pdf

Parent-reported child's close contact with non-household family members and their well-being during the COVID-19 pandemic: a cross-sectional survey

Short title: Children's perceived close contact with non-household family members and their well-being during COVID-19

Lisa Woodland[1,5*], Louise E. Smith[1,5], Samantha K. Brooks[1,5], Rebecca K. Webster[2], Richard Amlôt[3,5], Antonia Rubin[4], G James Rubin[1,5]

[1] Department of Psychological Medicine, King's College London, London, United Kingdom

[2] Department of Psychology, University of Sheffield, Sheffield, United Kingdom

[3] Behavioural Science and Insights Unit, UK Health Security Agency, Salisbury, United Kingdom

[4] Trustee at Weald of Kent Grammar School, Tonbridge, Kent, United Kingdom

[5] NIHR Health Protection Research Unit in Emergency Preparedness and Response

* Corresponding author

E-mail: lisa.woodland@kcl.ac.uk (LW)

Abstract

In England (UK), at the start of the COVID-19 pandemic the public were required to reduce their physical contacts to slow the spread of COVID-19. We investigated risk factors for children having: 1) close contact with family members from outside their household ("non-adherent behaviour"); and 2) low well-being (using the Revised Child Anxiety and Depression Scale). We conducted an online cross-sectional survey, completed at any location of the participant's choice between 8 and 11 June 2020 in parents (n = 2,010) who were aged eighteen years or over and had a school-aged child (4-18 years old). Parents reported that 15% (n = 309) of children had non-adherent contact and that 26% (n = 519) had low well-being. We used a series of binary logistic regressions to investigate associations between outcomes and child and parent characteristics. Children had higher odds of having non-household contact when they had special educational needs [adjusted odds ratio, 2.19 (95% CI, 1.47 to 3.27)], lower well-being [2.65 (95% CI, 2.03 to 3.46)], were vulnerable to COVID-19 [2.17 (95% CI, 1.45 to 3.25)], lived with someone who was over 70 years old [2.56 (95% CI, 1.55 to 4.24)] and their parent had low well-being [1.94 (95% CI, 1.45 to 2.58)]. Children had higher odds of lower well-being when they had special educational needs [4.13 (95% CI, 2.90 to 5.87)], were vulnerable to COVID-19 [3.06 (95% CI, 2.15 to 4.36)], lived with someone else who was vulnerable to COVID-19 [2.08 (95% CI, 1.64 to 2.64)], or lived with someone who was over 70 years old [2.41 (95% CI, 1.51 to 3.83)]. Many children came into contact with non-household family members, mainly for childcare. Factors relating to COVID-19, children's well-being and education were also important. If school closures are needed in future, addressing these issues may help reduce contact.

Key Words: coronavirus disease 2019; child; adherence; wellbeing; social behaviour; pathogen transmission

Introduction

On 23 March 2020, England went into “lockdown,” with the public required to stay at home to slow the spread of COVID-19 [1]. The public were only allowed to leave home for a limited number of reasons, which included for medical attention, to buy necessities (as infrequently as possible), for exercise (once a day) and to work if absolutely necessary and they were unable to work from home. Physical distancing guidelines were implemented: people from different households were not allowed to meet and individuals were asked to stay two meters away from people not in their household. Non-essential shops were closed, and schools were closed to most children. Children could attend school if they were on a ‘health care plan’ due to a specific medical or social need or if their parent(s) were employed in jobs that were essential to the COVID-19 response (‘key workers’), such as doctors, teachers, and supermarket employees [2]. After two months of lockdown guidance, the restrictions started to ease. On 11 May it was announced that people could return to work, the restrictions on spending time outside were lifted, and individuals were allowed to arrange meetings with one person from another household as long as this happened outside and a two meter distance was maintained [3]. From 1 June 2020, the rules relaxed further: six people from different households were able to meet outside (though still at a two meter distance) and more children were eligible to attend school, including children in early years (four to five years), year one (five to six years) and year six (10 to 11 years) [4].

While all the restrictions caused strain within society, the closure of schools was particularly problematic. A systematic review suggested that between 18% and 60% of children scored above risk thresholds for distress, anxiety and depressive symptoms between February and July 2020 and increases in other adverse behaviours were also reported, such as an increase in suicidal ideation, worsening of mood, emotional difficulties and conduct problems in children [5]. In a qualitative study that we conducted in April 2020 we found that parent’s and children’s characteristics (e.g., parent’s work status, children’s age, access to resources for home-schooling and entertainment activities and space inside and outside the home to exercise) impacted how well families could cope with the COVID-19 school closures [6].

Determining, at the height of a crisis, whether the epidemiological benefits of school closures in terms of reducing disease transmission will exceed the psychological, physical, educational, and social costs is a daunting challenge for those who must make this decision. One question that must be factored into decision making is ‘where do children go when their school is shut?’. In an ideal world (from an epidemiological perspective), parents would be able to ensure that children are placed under appropriate alternative supervision and remain apart from each other: continued mixing between children and other households would defeat the purpose of school closures. A systematic review of unplanned school closures prior to the COVID-19 pandemic found a 65% reduction in the mean total number of contacts for each student whilst schools were closed [7]. However, all 19 studies included in the review reported that children continued to meet with people from other households to some extent. A common reason for meeting others related to childcare. For example, one study found that children in households where special childcare arrangements

were needed during the closure had significantly higher odds of leaving home than children who were more independent and able to take care of themselves [8]. Parental concerns about the school closure also commonly reflected the difficulties they would face in trying to arrange childcare as well as concerns about lost education [7]. Previous research about children's adherence to medical treatment and encouraging healthy eating behaviours and physical exercise follow a similar pattern; factors relating to social networks and family cohesiveness or conflict [9, 10] and risk perceptions (e.g., perceived severity and vulnerability) [11, 12] are common in impacting adherence. These findings mirror a study that we conducted about family's adherence to England's COVID-19 guidelines. This study also found that parent's and children's characteristics, such as family income impacted adherence and that adherence may be more challenging for families with low psychological and physical ability [13].

In the context of the COVID-19 pandemic, issues around childcare while schools were closed were of particular concern because of a worry that grandparents might be called on to look after children in some families, putting older adults at increased risk of infection [14]. It was not just policy makers who worried about this, many children were themselves worried about their grandparents' vulnerability to COVID-19 [15] and of the risk that they themselves might infect their grandparents [16]. The extent to which these worries were borne out remains unclear.

Of course, while keeping children separate from each other and away from their grandparents might reduce disease transmission, it does present other risks. For example, a reduction in physical activity levels as a result of staying at home can affect physical and mental well-being [5]. While parents were advised to ensure their

children engaged in physical activity during the initial lockdown, the extent to which this occurred, and the impact of that initial period of isolation and inactivity is uncertain. Some parents and children may also have been very cautious about the risks associated with COVID-19, engaging in additional protective behaviours that were not explicitly recommended by the Government and which may have exerted an additional toll on well-being [17, 18].

In this study we investigated the risk factors associated with parents reporting children having: 1) close contact with family members from outside their household; and 2) low well-being during the first COVID-19 lockdown in the UK. Considering the evidence discussed above, we specifically explored whether close contact with non-household family members and low child well-being were associated with: parents' age, gender, and employment characteristics, children's age, gender and special educational needs status, and household's vulnerability to, perceptions about and behaviours in relation to COVID-19.

Materials and Methods

Design

We commissioned a market research company, BMG Research [19] to administer an online cross-sectional survey between 8 and 11 June 2020. Data collection occurred after lockdown restrictions had begun to be eased. At this stage in the pandemic, schools had re-opened to children in early years (four to five years), year one (five to six years) and year six (1 to 11 years). However, physical distancing restrictions remained in place throughout society, and while up to six non-household members could meet, this had to be outside and at a two-meter distance.

We have previously published data from this survey relating to parental perceptions of the hygiene procedures within schools [20] and investigating why some parents did not send their eligible children back to school [21].

Participants

Participants (n=2,447) were recruited from BMG Research's panel. To achieve a sample that was broadly representative of the population, BMG Research monitored region, child age, child gender, parent/guardian age, and parent/guardian gender. Participants were eligible for the study if they were aged eighteen years or over, lived in England, and were a parent or guardian to a school-aged child (4-18 years old) who usually lived with them. One-hundred and eighty-three participants were screened out for non-eligibility by BMG, 226 participants dropped out after starting the survey and 28 completed but were removed for reasons related to quality control, such as completing the survey quickly compared to the average (14 minutes) and median (11 minutes) survey time or for 'straight-lining' (selecting the same option for every question) suggesting inattention to the questions. These issues are common in online surveys [22]. Two thousand and ten participants remained.

Quota sampling, as used in this study, is a non-probability based approach and we have therefore not reported the response rates as they are not helpful indicators of nonresponse bias. The response rates for each quota will differ, the denominator for each is essentially unknown, and the predominant source of bias is in the make-up of the underlying panels that are being recruited from, rather than whether the sample is representative of the panel [23].

The sample fell within five percentage points of the national population by the child's gender, key stage, and type of school attended against the known distribution for school children in England [24].

Participants were paid in points that could be accumulated by the participant and exchanged later for money. Our survey paid points equivalent to about £0.60.

The research was approved by the Psychiatry, Nursing and Midwifery Research Ethics Subcommittee at King's College London (LRS-19/20-18787). An information sheet was provided at the start of the survey, which described the purpose of the study. Participants were also informed of the study process, and they provided written consent before taking part.

Study Materials

The full survey is available in the supporting information (S1 Text).

All participants answered questions referring to their child who had the most recent birthday. In rare cases where children shared a birthday, we asked the parent to select one child.

Outcome one: Child's physical close contact with non-household family members

We asked parents to choose from a list of seven options about people who the child had come into close contact with in the past 24 hours ("someone [child] lives with;" "friends or other children who [child] does not live with"; "a babysitter, nanny or childminder;" "family member aged under 70 who [child] does not live with;" "family member aged over 70 who [child] does not live with;" "other children, not already reported above"; and "other adults, not already reported above"). We made

clear in the question that “by close contact we mean closer than 2 meters, for fifteen minutes or more,” which was the UK Government’s definition of close contact [25]. Parents were asked to report all the options that applied.

We created a binary variable to indicate whether the child had close contact with a family member from outside their household, defined as a “non-household close contact.” This included children reported as having had close contact with either “a family member aged under 70 who [child] does not live with” or “a family member aged over 70 who [child] does not live with.”

Outcome two: Child’s well-being

We asked parents to report the child’s well-being using two subscales from the Revised Child Anxiety and Depression Scale (RCADS): the generalised anxiety disorder (GAD) sub-scale and the major depressive disorder (MDD) sub-scale [26]. The GAD sub-scale asks parents to respond to six statements about their child (e.g., “my child worries about things”; and “my child worries that something awful will happen to someone in the family”). The MDD sub-scale asks parents to respond to ten statements about their child (e.g., “my child feels sad or empty”; “nothing is much fun for my child”; and “my child has trouble sleeping”). Parents can respond “never,” “sometimes,” “often,” and “always.”

We created a binary variable to indicate well-being in the child. The variable was recoded using SPSS syntax supplied by the RCADS authors, which assigns a value against each answer from 0 (“never”) to 3 (“always”) on the GAD and MDD RCADS sub-scales and creates a total score for each sub-scale [26]. The total score is turned into a t-score, normalising the RCADS scores within the population, by child’s age and gender. A t-score of 65 (approximately in the top 7% of un-referred

young people of the same age) on either the GAD or MDD sub-scale was our well-being cut off. “Lower well-being” represents a child with a medium or severe risk of clinical mental illness and “higher well-being” represents a child with low risk of clinical mental illness.

Predictor variables:

Parent and child personal characteristics

We asked parents to report their gender, age, region, household income, employment status, and if employed, whether they were working from home, level of education, marital status, ethnicity, and key worker status. We asked parents to report the child’s gender, age, school year, and whether the child had special educational needs. We also asked whether anyone within the household was aged over 70 years old or had a health condition that made them vulnerable to COVID-19 and whether they had access to outside space.

We recoded household income (less than £5,000; £5,000-£9,999; £10,000-£14,999; £15,000-£19,999; £20,000-£24,999; £25,000-£29,999; £30,000-£34,999; £35,000-£39,999; £40,000-£44,999; £45,000-£49,999; £50,000-£59,999; £60,000-£69,999; £70,000-£84,999; £85,000-£99,999; more than £100,000), employment status (full time paid job (31+ hours); part time paid job (<31 hours); doing paid work on a self-employed basis or within your own business; employed, but currently furloughed; student / on a government training programme (Nation Traineeship/Modern Apprenticeship); out of work (6 months or less); out of work (more than 6 months); looking after home / homemaker; retired; disabled OR long-term sick; unpaid work for a business, community or voluntary organisation), parent

education level (PhD/Doctor; Master's; Bachelor's Degree or equivalent (such as a NVQ level 5); higher education (such as a HND or a NVQ level 4); A-level or equivalent (such as Scottish Highers or NVQ level 3); GCSE and below (such as O level or an RSA Diploma); Other qualifications (Such as NVQ level 1); No qualifications), marital status (single (i.e. never married and never registered as a same sex civil-partnership); co-habiting with partner (but never married or been in a civil partnership); civil partnership; married; separated, but still legally married / in a civil partnership; divorced / civil partnership legally dissolved; widowed / surviving partner from a same-sex civil partnership), and ethnicity (English/Welsh/Scottish/Northern Irish/British; Irish; Gypsy or Irish Traveller; White and Black Caribbean; White and Black African; White and Asian; Indian; Pakistani; Bangladeshi; Chinese; Caribbean; African; Arab; any other (please specify)) into binary variables.

We recoded parent age, key worker status, and access to outside space into categorical variables, as shown in the results tables. We recoded child school year into Key Stages as used in the English education system (Early years = ages 4 to 5; Key Stage 1 = ages 5 to 7; Key Stage 2 = ages 7 to 11; Key Stage 3 = ages 11 to 14; Key Stage 4 = ages 14 to 16; Years 12 and 13 = ages 16 to 18). We created two binary variables to indicate whether the child, and someone in the household (other than the child) had a health condition that might make them particularly vulnerable to COVID-19. The responses "yourself [participant]" and "anyone else you live with" were combined into one variable to indicate that the child lived with someone vulnerable to COVID-19.

Parent's well-being

We asked parents to report their well-being using the Patient-Health Questionnaire-4 (PHQ4) [27], which asks "over the last two weeks, how often have you been bothered by the following problems:" "feeling nervous, anxious or on the edge;" "not being able to stop or control worrying;" "little interest or pleasure in doing things;" and "feeling down, depressed, or hopeless." Parents can respond "not at all," "several days," "more than half the days," and "nearly every day."

We created a binary variable to indicate low well-being in the parent. We assigned a value against each answer from 0 ("not at all") to 3 ("nearly every day") on the PHQ4 and summed responses (range 0 to 12). We used a cut off score of 5 or above to indicate low well-being in the parent, indicating moderate or severe risk of clinical anxiety or depression.

Child's activities outside the home

We asked parents how many times the child had left the home in the past seven days: "to go to the shops for groceries, toiletries, or medicines;" "to go to the shops for other items;" "for exercise;" "for a medical need (e.g., an outpatient appointment);" "to go to school;" "to provide help to someone else;" "to meet friends;" to meet family members who they did not live with; and "for another reason."

Items about the child's activities outside the home were used as continuous variables.

Behaviours that parents and children had followed

We asked parents to report the behaviours that they or their children had followed in the past 7 days because of the risk of COVID-19 (e.g., "washed your hands thoroughly and regularly," "stayed 2m (3 steps) away from people you do not

live with when outside your home," "washed your clothes when you have returned home and "washed [child]'s clothes when she/he has returned home"). Parents could respond "yes" or "no" to each statement. Out of the 11 statements that we asked about, two were recommended and nine were not recommended by UK Government at the time of the study.

We created a continuous variable to indicate the COVID-19 behaviours that parents and their child had followed by combining all 11 responses to the statements about what the parent or child had done in the past seven days because of the risk of COVID-19.

Statements about lockdown

We included 16 statements about lockdown, which included questions about COVID-19 (e.g., "if [child] goes out, she/he is likely to catch coronavirus"), schooling (e.g., "[child] is keeping up with his/her schoolwork") and home environment (e.g., "in the past 7 days, [child] has been bored"). Parents responded to each statement using a five-point Likert-scale from "strongly agree" to "strongly disagree," or "not applicable."

Items about lockdown were used as continues variables.

Missing data

We took the pragmatic approach to code the responses "don't know", "not applicable", "prefer not to say" and "prefer to self-describe" as missing data. We adopted this approach for outcome variables because it was not possible to categorise participants as adherent or not, or as having good wellbeing or not. For predictors we adopted this approach to provide a clearer understanding of the

difference between endorsing, or not endorsing, each variable in terms of its impact on our outcomes.

Validity and Reliability

Previous studies have used RCADS [28], and the PHQ-4 [29], and they have both demonstrated good validity and reliability. The survey questions that related to COVID-19 were designed for this study based on our previous research [6], they have face validity and we copied the wording of official Government guidance where applicable. Five parents and one school trustee (see public involvement section) piloted the survey. Beyond this we do not have psychometric data about the validity and reliability of the questions.

Public involvement

A school trustee contributed to the development of the survey materials and co-authored this paper. The survey questions were reviewed by five parents of school children who also piloted the survey before publication. The feedback we received resulted in minor changes to the wording of some survey questions for clarity.

Analysis

We ran a series of binary logistic regressions using SPSS [30, 31] investigating the univariable associations between our two outcomes and each of our predictor variables. We ran a second set of binary logistic regressions controlling for child and parent characteristics (participant gender, age, region, household income, employment status, education level, marital status, ethnicity, and the child's gender and school year).

We applied a Bonferroni correction to our results (p≤0.001) due to running many analyses. Only associations that met this level are discussed narratively in the results section.

Results

Most parents were: female (53%); between 36 and 45 years of age (43%); lived in London (17%); had a household income over £35,000 (55%); working (83%); highly educated (57%); married or co-habiting (84%); and of white ethnicity (87%) (Table 1).

Table 1: Parent, child and household characteristics (n = 2,010)

Variable	Level	n, (%)*
Parent gender	Male	931 (46)
	Female	1065 (53)
Parent age	18-35 years	394 (20)
	36-45 years	868 (43)
	46 years ≥	748 (37)
Region	East Midlands	151 (8)
	East of England	219 (11)
	North East	118 (6)
	North West	273 (14)
	South East	335 (17)
	South West	163 (8)
	West Midlands	211 (10)
	Yorkshire & the Humber	195 (10)
	London	345 (17)
Household income	≤ £34,999	804 (40)
	£35,000 ≥	1114 (55)
Employment status[1]	Working	1677 (83)
	Not working	321 (16)
Parent Working from home[2]	Yes	940 (59)
	No	628 (40)
Education level	≤ A-level	855 (43)
	Degree ≥	1139 (57)
Marital status	Living alone	327 (16)
	Married/cohabiting	1683 (84)
Ethnicity	White	1753 (87)
	BAME	241 (12)
Key worker status	Both parents	195 (10)
	One parent	847 (42)
	No	956 (48)
Child gender	Boy	1063 (53)
	Girl	947 (47)
Child school year	Early Years (ages 4 to 5)	101 (5)
	Key Stage 1 (ages 5 to 7)	356 (18)
	Key Stage 2 (ages 7 to 11)	675 (34)
	Key Stage 3 (ages 11 to 14)	395 (20)
	Key Stage 4 (ages 14 to 16)	328 (16)
	Years 12 & 13 (ages 16 to 18)	155 (8)
	Yes	161 (8)

Child had special educational needs	No	1832 (91)
Child lower well-being	Yes	519 (26)
	No	1491 (74)
Parent lower well-being	Yes	391 (19)
	No	1619 (81)
Child vulnerable COVID-19	Yes	157 (8)
	No	1826 (91)
Household vulnerable COVID-19	Yes	510 (25)
	No	1311 (65)
Someone over 70 years	Yes	87 (4)
	No	1923 (96)
COVID-19 behaviours followed by child and parent	Continuous (0=followed no behaviours, 11=followed all behaviours)	N=2,010, M=5.60, SD=3.10
Access to outside space[3]	Garden	1784 (89)
	Other[3]	157 (8)
	No	69 (3)

*Percentages may not total 100 due to rounding errors and missing data.
[1] Working includes students and volunteers
[2] Question only offered to participants who reported working in a paid job (full-time, part-time, and self-employed) and not to participants who reported being a student, on furlough and a volunteer.
[3] Participants that reported no access to a garden but had access to other outdoor spaces such as patio, terrace, and balcony
Abbreviations: n = number; % = percentage; M = mean; SD = standard deviation

Risk factors associated with children's non-household close contact

Of the 2010 parent responses, 15% (95% CI 14% to 17%, n=309) of children were perceived by the parents to have had close contact with a family member that they did not live with in the past 24 hours. This included 9% (n=189) who had close contact with a family member aged under 70 years and 6% (n=120) who had close contact with a family member aged 70 years or older, which includes n = 45 who had close contact with non-household family members under and over 70 years of age. The parent and child characteristics associated with children who had non-household close contact are shown in Table 2 (see Table 7, Appendix A for the descriptives). Parents with lower well-being had higher odds of reporting that their child had non-household close contact [adjusted odds ratio 1.94 (95% confidence interval (CI), 1.45 to 2.58)]. Parents who reported that their child had special educational needs, lower well-being, were vulnerable to COVID-19, and lived with someone who was over 70 years of age had higher odds of reporting that their child

had had non-household close contact [adjusted odd ratios, 2.19 (95% CI, 1.47 to 3.27); 2.65 (95% CI, 2.03 to 3.46); 2.56 (95% CI, 1.55 to 4.24), respectively].

Table 3 shows the associations between children who had non-household close contact, as perceived by their parents, and the predictor variables relating to parent perceptions about lockdown (see Table 8, Appendix B for the descriptives). Parents' agreement that their child had extra support at school before the closures, were upset about not seeing other family members that they did not live with, and that the parent had found it hard to keep up with work or other important commitments was associated with their children's non-household close contact [adjusted odd ratios, 0.82 (95% CI, 0.74 to 0.90); 0.84 (95% CI, 0.75 to 0.93); 0.78 (95% CI, 0.70 to 0.87), respectively].

The behaviours that families followed, perceived by parents because of the risk of COVID-19 are presented in Table 9 (see Appendix C).

Table 2 Binary logistic regression comparing parent and child characteristics and associations with children's non-household close contact

Variable	Level	Unadjusted odds Ratio (95% CI)	P-value	Adjusted odds ratio (95% CI) †	P-value
Parent gender	Male	0.88 (0.69 to 1.12)	0.30	0.97 (0.74 to 1.27)	0.80
	Female	Reference		Reference	
Parent age	18-35 years	**2.13** (1.54 to 2.95)**	**<0.001**	**1.28* (1.25 to 2.65)**	**0.002**
	36-45 years	1.29 (0.97 to 1.72)	0.09	1.17 (0.85 to 1.62)	0.34
	46 years ≥	Reference		Reference	
Region	East Midlands	0.73 (0.43 to 1.24)	0.25	0.69 (0.40 to 1.21)	0.19
	East of England	0.76 (0.48 to 1.21)	0.25	0.77 (0.47 to 1.26)	0.30
	North East	0.58 (0.31 to 1.08)	0.08	0.55 (0.28 to 1.06)	0.07
	North West	0.76 (0.50 to 1.17)	0.21	0.83 (0.52 to 1.32)	0.43
	South East	0.79 (0.53 to 1.18)	0.25	0.84 (0.55 to 1.28)	0.43
	South West	0.60 (0.35 to 1.03)	0.07	0.61 (0.35 to 1.08)	0.09
	West Midlands	0.66 (0.41 to 1.07)	0.09	0.63 (0.37 to 1.05)	0.08
	Yorkshire & the Humber	0.91 (0.58 to 1.44)	0.69	0.88 (0.54 to 1.44)	0.61
	London	Reference		Reference	
Household income	≤ £34,999	**1.34* (1.05 to 1.72)**	**0.02**	**1.44* (1.08 to 1.92)**	**0.01**
	£35,000 ≥	Reference		Reference	
Employment status[1]	Working	0.27 (1.21 to 0.86 to 1.72)	0.27	1.12 (0.77 to 1.63)	0.57
	Not working	Reference		Reference	
Parent Working from home[2]	Yes	**1.53* (1.15 to 2.05)**	**0.004**	^^^	^^^
	No	Reference		Reference	
Education level	≤ A-level	**0.77* (0.60 to 0.98)**	**0.04**	**0.70* (0.53 to 0.93)**	**0.01**
	Degree ≥	Reference		Reference	
Marital status	Living alone	**1.46* (1.08 to 1.98)**	**0.01**	1.36 (0.98 to 1.90)	0.07
	Married/cohabiting	Reference		Reference	
Ethnicity	White	0.97 (0.67 to 1.41)	0.88	1.25 (0.83 to 1.87)	0.28
	BAME	Reference		Reference	
Key worker status	Both parents	1.18 (0.76 to 1.84)	0.47	1.18 (0.74 to 1.90)	0.49
	One parent	**1.65** (1.28 to 2.14)**	**<0.001**	**1.53* (1.16 to 2.02)**	**0.002**
	No	Reference		Reference	
Child gender	Boy	1.14 (0.89 to 1.46)	0.29	1.14 (0.88 to 1.48)	1.14
	Girl	Reference		Reference	
Child school year	Early Years	1.53 (0.73 to 3.18)	0.26	1.22 (0.55 to 2.71)	0.63
	Key Stage 1	**2.24* (1.28 to 3.94)**	**0.01**	**2.11* (1.13 to 3.94)**	**0.02**
	Key Stage 2	1.40 (0.81 to 2.41)	0.23	1.28 (0.70 to 2.33)	0.42
	Key Stage 3	1.48 (0.84 to 2.63)	0.18	1.53 (0.83 to 2.83)	0.17
	Key Stage 4	1.10 (0.60 to 2.01)	0.77	1.21 (0.64 to 2.30)	0.56
	Years 12 & 13	Reference		Reference	
Child had special educational needs	Yes	**2.16** (1.49 to 3.14)**	**<0.001**	**2.19** (1.47 to 3.27)**	**<0.001**
	No	Reference		Reference	
Child lower well-being	Yes	**2.66** (2.07 to 3.42)**	**<0.001**	**2.65** (2.03 to 3.46)**	**<0.001**
	No	Reference		Reference	
Parent lower well-being	Yes	**2.15** (1.64 to 2.82)**	**<0.001**	**1.94** (1.45 to 2.58)**	**<0.001**
	No	Reference		Reference	
Child vulnerable COVID-19	Yes	**2.50** (1.73 to 3.61)**	**<0.001**	**2.17** (1.45 to 3.25)**	**<0.001**
	No	Reference		Reference	
Household vulnerable COVID-19	Yes	1.16 (0.87 to 1.54)	0.32	1.17 (0.87 to 1.59)	0.30
	No	Reference		Reference	

Someone over 70 years	Yes	**2.77** (1.74 to 4.42)**	**<0.001**	**2.56** (1.55 to 4.24)**	**<0.001**
	No	Reference		Reference	
COVID-19 behaviours followed by child and parent	Continuous (0=followed no behaviours, 11=followed all behaviours)	**1.06* (1.02 to 1.11)**	**0.002**	1.04 (0.99 to 1.08)	0.10
Access to outside space[3]	Garden	0.73 (0.40 to 1.38)	0.33	0.88 (0.44 to 1.75)	0.71
	Other[3]	1.28 (0.63 to 2.60)	0.49	1.25 (0.58 to 2.73)	0.71
	No	Reference		Reference	

* $p \leq$ *·05* and formatted bold
** $p \leq$ *·001* and formatted bold
Some results are rounded to 3 rather than 2 decimal places to distinguish between $p \leq$ *·05* and $p \leq$ *·001*
† When interpretating the predictor listed in the row, we controlled for the other variables listed here: participant gender, age, region, household income, employment status, education level, marital status, ethnicity, and the child's gender and school year
^^^ Not included variable highly correlated with employment variable
[1] Working includes students and volunteers
[2] Question only offered to participants who reported working in a paid job (full-time, part-time, and self-employed) and not to participants who reported being a student, on furlough and a volunteer.
[3] Participants that reported no access to a garden but had access to other outdoor spaces such as patio, terrace, and balcony
Abbreviations: CI = confidence interval

Table 3 Binary logistic regression comparing statements about lockdown and associations with children's non-household close contact. Close contact is defined by a child's close contact with a family member outside the household (n=309)

Statement	Level	Unadjusted odds ratio (95%, CI)	P-value	Adjusted odds ratio (95%, CI) †	P-value
If child goes out, she/he is likely to catch coronavirus	5-point Likert-scale (1=strongly agree, 5=strongly disagree)	**0.85* (0.75 to 0.95)**	**0.004**	0.89 (0.79 to 1.01)	0.07
If child goes out, she/he is likely to bring coronavirus back into our home	5-point Likert-scale (1=strongly agree, 5=strongly disagree)	**0.86* (0.77 to 0.96)**	**0.01**	0.91 (0.81 to 1.03)	0.13
Child is keeping up with his/her schoolwork	5-point Likert-scale (1=strongly agree, 5=strongly disagree)	**1.16* (1.04 to 1.29)**	**0.01**	**1.14* (1.02 to 1.27)**	**0.03**
I feel confident helping child with her/his schoolwork	5-point Likert-scale (1=strongly agree, 5=strongly disagree)	1.06 (0.94 to 1.19)	0.32	1.08 (0.96 to 1.23)	0.20
I feel supported by child's school	5-point Likert-scale (1=strongly agree, 5=strongly disagree)	1.00 (0.89 to 1.12)	0.96	0.99 (0.68 to 1.12)	0.87
I have access to all the resources that child needs to do her/his schoolwork	5-point Likert-scale (1=strongly agree, 5=strongly disagree)	1.05 (0.94 to 1.18)	0.38	1.09 (0.96 to 1.23)	0.18
During lockdown, child has learned about important things she/he wouldn't normally learn at school	5-point Likert-scale (1=strongly agree, 5=strongly disagree)	0.98 (0.87 to 1.11)	0.74	1.03 (0.30 to 1.17)	0.69
In the past 7 days, child has been bored	5-point Likert-scale (1=strongly agree, 5=strongly disagree)	**0.84** (0.75 to 0.93)**	**0.001**	**0.85* (0.76 to 0.95)**	**0.004**
In the past 7 days, my household has had a regular structure to the day	5-point Likert-scale (1=strongly agree, 5=strongly disagree)	1.12 (1.00 to 1.26)	0.05	1.09 (0.96 to 1.23)	0.18
In the past 7 days, child has kept in touch with her/his friends	5-point Likert-scale (1=strongly agree, 5=strongly disagree)	1.00 (0.90 to 1.12)	0.96	0.95 (0.84 to 1.07)	0.36
Child is worried about coronavirus	5-point Likert-scale (1=strongly agree, 5=strongly disagree)	0.98 (0.88 to 1.09)	0.72	0.99 (0.88 to 1.10)	0.82
In the past 7 days, child has felt upset about not seeing other family members who do not live with us	5-point Likert-scale (1=strongly agree, 5=strongly disagree)	**0.81** (0.73 to 0.89)**	**<0.001**	**0.84** (0.75 to 0.93)**	**0.001**
In the past 7 days, I have found it hard to keep up with work or other important commitments	5-point Likert-scale (1=strongly agree, 5=strongly disagree)	**0.75** (0.67 to 0.83)**	**<0.001**	**0.78** (0.70 to 0.87)**	**<0.001**
In the past 7 days, people in my household have been getting along well	5-point Likert-scale (1=strongly agree, 5=strongly disagree)	1.13 (1.00 to 1.28)	0.05	1.07 (0.94 to 1.22)	0.28
I am worried about the financial impact of lockdown measures	5-point Likert-scale (1=strongly agree, 5=strongly disagree)	0.92 (0.83 to 1.02)	0.13	0.93 (0.83 to 1.03)	0.18
Before the school closures, child had extra support at school	5-point Likert-scale (1=strongly agree, 5=strongly disagree)	**0.80** (0.73 to 0.88)**	**<0.001**	**0.82** (0.74 to 0.90)**	**<0.001**

* *p*≤ *·05* and formatted bold

** *p*≤ *·001* and formatted bold

Some results are rounded to 3 rather than 2 decimal places to distinguish between *p*≤ *·05* and *p*≤ *·001*

† When interpreting the predictor listed in the row, we controlled for the other variables listed here: participant gender, age, region, household income, employment status, education level, marital status, ethnicity, and the child's gender and school year

Abbreviations: CI = confidence interval

Risk factors associated with children's lower well-being

Of the 2010 parent responses, 26% (95% CI 24% to 28%, n=519) reported that their child was perceived to have had low well-being. The parent and child characteristics associated with child low well-being are shown in Table 4 (see Table 10, Appendix D for the descriptives). Parents aged between 18 and 35 years old, who were a key worker and parents with lower well-being had higher odds of reporting that their child had lower well-being [adjusted odds ratio, 1.92 (95% CI, 1.40 to 2.64); 1.51 (95% CI, 1.20 to 1.90); 7.26 (95% CI, 5.62 to 9.38), respectively]. Parents who reported that their child had had special educational needs, were vulnerable to COVID-19, lived with someone else who was also vulnerable to COVID-19, and lived with someone that was over 70 years old also had higher odds of reporting that their child had lower well-being [adjusted odd ratios, 4.13 (95% CI, 2.90 to 5.87)]; 3.06 (95% CI, 2.15 to 4.36); 2.08 (95% CI, 1.64 to 2.64)]; 2.41 (95% CI, 1.51 to 3.83), respectively]. Parents who reported that their household followed multiple precautionary behaviours because of the risk of COVID-19 had higher odds of reporting that their child had lower well-being [adjusted odds ratio, 1.11 (95% CI, 1.07 to 1.15)]. All lockdown statements bar two were associated with lower well-being (Table 5) (see Table 11, Appendix E for descriptives).

Table 4 Binary logistic regression comparing parent and child characteristics and associations with children's lower well-being (n=519)

Variable	Level	Unadjusted odds Ratio (95% CI)	P-value	Adjusted odds ratio (95% CI) †	P-value
Parent gender	Male	0.91 (0.74 to 1.11)	0.36	0.92 (0.74 to 1.15)	0.46
	Female	Reference		Reference	
Parent age	18-35 years	**1.67** (1.27 to 2.19)**	**<0.001**	**1.92** (1.40 to 2.64)**	**<0.001**
	36-45 years	1.11 (0.88 to 1.40)	0.38	1.25 (0.96 to 1.62)	0.09
	46 years ≥	Reference		Reference	
Region	East Midlands	1.27 (0.84 to 1.91)	0.26	1.21 (0.78 to 1.88)	0.39
	East of England	0.88 (0.60 to 1.29)	0.51	0.93 (0.61 to 1.39)	0.71
	North East	0.72 (0.44 to 1.18)	0.20	0.77 (0.46 to 1.30)	0.33
	North West	0.80 (0.56 to 1.15)	0.23	0.90 (0.61 to 1.33)	0.61
	South East	0.90 (0.64 to 1.26)	0.53	0.90 (0.62 to 1.29)	0.56
	South West	0.98 (0.64 to 1.48)	0.91	1.04 (0.67 to 1.63)	0.85
	West Midlands	0.79 (0.54 to 1.18)	0.25	0.75 (0.49 to 1.15)	0.19
	Yorkshire & the Humber	0.72 (0.48 to 1.09)	0.12	0.71 (0.46 to 1.11)	0.13
	London	Reference		Reference	
Household income	≤ £34,999	1.12 (0.91 to 1.37)	0.29	1.08 (0.85 to 1.37)	0.54
	£35,000 ≥	Reference		Reference	
Employment status[1]	Working	1.12 (0.85 to 1.48)	0.44	1.12 (0.82 to 1.52)	0.48
	Not working	Reference		Reference	
Parent Working from home[2]	Yes	1.16 (0.92 to 1.47)	0.20	^^^	^^^
	No	Reference		Reference	
Education level	≤ A-level	0.92 (0.75 to 1.12)	0.39	0.92 (0.74 to 1.16)	0.50
	Degree ≥	Reference		Reference	
Marital status	Living alone	1.17 (0.90 to 1.53)	0.24	1.15 (0.87 to 1.53)	0.33
	Married/cohabiting	Reference		Reference	
Ethnicity	White	0.93 (0.69 to 1.26)	0.65	1.06 (0.76 to 1.48)	0.73
	BAME	Reference		Reference	
Key worker status	Both parents	**1.70* (1.21 to 2.39)**	**0.002**	**1.71* (1.18 to 2.46)**	**0.004**
	One parent	**1.60** (1.29 to 1.98)**	**<0.001**	**1.51** (1.20 to 1.90)**	**<0.001**
	No	Reference		Reference	
Child gender	Boy	1.20 (0.98 to 1.47)	0.07	1.22 (0.98 to 1.50)	0.07
	Girl	Reference		Reference	
Child school year	Early Years	0.99 (0.55 to 1.79)	0.98	0.71 (0.38 to 1.32)	0.28
	Key Stage 1	1.13 (0.73 to 1.75)	0.59	0.80 (0.50 to 1.29)	0.37
	Key Stage 2	0.97 (0.65 to 1.47)	0.90	0.71 (0.46 to 1.10)	0.13
	Key Stage 3	1.22 (0.79 to 1.87)	0.37	1.03 (0.66 to 1.61)	1.61
	Key Stage 4	1.36 (0.88 to 2.11)	0.17	1.15 (0.73 to 1.80)	0.55
	Years 12 & 13	Reference		Reference	
Child has special educational needs	Yes	**4.24** (3.05 to 5.89)**	**<0.001**	**4.13** (2.90 to 5.87)**	**<0.001**
	No	Reference		Reference	
Parent low well-being	Yes	**6.77** (5.33 to 8.60)**	**<0.001**	**7.26** (5.62 to 9.38)**	**<0.001**
	No	Reference		Reference	
Child vulnerable COVID-19	Yes	**3.34** (2.40 to 4.65)**	**<0.001**	**3.06** (2.15 to 4.36)**	**<0.001**
	No	Reference		Reference	
Household vulnerable COVID-19	Yes	**2.01** (1.60 to 2.51)**	**<0.001**	**2.08** (1.64 to 2.64)**	**<0.001**
	No	Reference		Reference	
Someone over 70 years	Yes	**2.69** (1.75 to 4.16)**	**<0.001**	**2.41** (1.51 to 3.83)**	**<0.001**
	No	Reference		Reference	
COVID-19 behaviours followed by child and parent	Continuous (0=followed no behaviours,	**1.11** (1.07 to 1.14)**	**<0.001**	**1.11** (1.07 to 1.15)**	**<0.001**

	11=followed all behaviours)				
Access to outside space[3]	Garden	0.89 (0.52 to 1.53)	0.67	1.10 (0.60 to 2.03)	0.76
	Other[3]	1.19 (0.64 to 2.23)	0.58	1.36 (0.68 to 2.73)	0.38
	No	Reference		Reference	

* $p \leq \cdot 05$ and formatted bold

** $p \leq \cdot 001$ and formatted bold

Some results are rounded to 3 rather than 2 decimal places to distinguish between $p \leq \cdot 05$ and $p \leq \cdot 001$

† When interpretating the predictor listed in the row, we controlled for the other variables listed here: participant gender, age, region, household income, employment status, education level, marital status, ethnicity, and the child’s gender and school year

^^^ Not included variable highly correlated with employment variable

[1] Working includes students and volunteers

[2] Question only offered to participants who reported working in a paid job (full-time, part-time, and self-employed) and not to participants who reported being a student, on furlough and a volunteer.

[3] Participants that reported no access to a garden but had access to other outdoor spaces such as patio, terrace, and balcony

Abbreviations: CI = confidence interval

Table 5 Binary logistic regression comparing statements about lockdown and associations with children's lower well-being (n=519)

Statement	Level	Unadjusted odds ratio (95%, CI)	P-value	Adjusted odds ratio (95%, CI) †	P-value
If child goes out, she/he is likely to catch coronavirus	5-point Likert-scale (1=strongly agree, 5=strongly disagree)	**0.63** (0.57 to 0.70)**	**<0.001**	**0.63** (0.57 to 0.70)**	**<0.001**
If child goes out, she/he is likely to bring coronavirus back into our home	5-point Likert-scale (1=strongly agree, 5=strongly disagree)	**0.63** (0.58 to 0.70)**	**<0.001**	**0.63** (0.57 to 0.70)**	**<0.001**
Child is keeping up with his/her schoolwork	5-point Likert-scale (1=strongly agree, 5=strongly disagree)	**1.43** (1.31 to 1.57)**	**<0.001**	**1.46** (1.33 to 1.60)**	**<0.001**
I feel confident helping child with her/his schoolwork	5-point Likert-scale (1=strongly agree, 5=strongly disagree)	**1.34** (1.22 to 1.48)**	**<0.001**	**1.34** (1.21 to 1.48)**	**<0.001**
I feel supported by child's school	5-point Likert-scale (1=strongly agree, 5=strongly disagree)	**1.26** (1.15 to 1.38)**	**<0.001**	**1.24** (1.12 to 1.37)**	**<0.001**
I have access to all the resources that child needs to do her/his schoolwork	5-point Likert-scale (1=strongly agree, 5=strongly disagree)	**1.30** (1.18 to 1.43)**	**<0.001**	**1.31** (1.18 to 1.44)**	**<0.001**
During lockdown, child has learned about important things she/he wouldn't normally learn at school	5-point Likert-scale (1=strongly agree, 5=strongly disagree)	**1.12* (1.01 to 1.24)**	**0.02**	**1.13* (1.02 to 1.25)**	**0.02**
In the past 7 days, child has been bored	5-point Likert-scale (1=strongly agree, 5=strongly disagree)	**0.68** (0.62 to 0.75)**	**<0.001**	**0.68** (0.61 to 0.75)**	**<0.001**
In the past 7 days, my household has had a regular structure to the day	5-point Likert-scale (1=strongly agree, 5=strongly disagree)	**1.23** (1.12 to 1.36)**	**<0.001**	**1.23** (1.11 to 1.36)**	**<0.001**
In the past 7 days, child has kept in touch with her/his friends	5-point Likert-scale (1=strongly agree, 5=strongly disagree)	**1.15* (1.05 to 1.25)**	**0.01**	1.67 (0.89 to 3.15)	0.11
Child is worried about coronavirus	5-point Likert-scale (1=strongly agree, 5=strongly disagree)	**0.60** (0.54 to 0.66)**	**<0.001**	**0.59** (0.53 to 0.65)**	**<0.001**
In the past 7 days, child has felt upset about not seeing other family members who do not live with us	5-point Likert-scale (1=strongly agree, 5=strongly disagree)	**0.64** (0.59 to 0.70)**	**<0.001**	**0.63** (0.57 to 0.69)**	**<0.001**
In the past 7 days, I have found it hard to keep up with work or other important commitments	5-point Likert-scale (1=strongly agree, 5=strongly disagree)	**0.56** (0.51 to 0.61)**	**<0.001**	**0.55** (0.49 to 0.60)**	**<0.001**
In the past 7 days, people in my household have been getting along well	5-point Likert-scale (1=strongly agree, 5=strongly disagree)	**1.47** (1.33 to 1.63)**	**<0.001**	**1.46** (1.31 to 1.63)**	**<0.001**
I am worried about the financial impact of lockdown measures	5-point Likert-scale (1=strongly agree, 5=strongly disagree)	**0.78** (0.71 to 0.85)**	**<0.001**	**0.77** (0.70 to 0.85)**	**<0.001**
Before the school closures, child had extra support at school	5-point Likert-scale (1=strongly agree, 5=strongly disagree)	**0.68** (0.63 to 0.74)**	**<0.001**	**0.69** (0.63 to 0.75)**	**<0.001**

* *p≤ ·05* and formatted bold
** *p≤ ·001* and formatted bold
Some results are rounded to 3 rather than 2 decimal places to distinguish between *p≤ ·05* and *p≤ ·001*
† When interpretating the predictor listed in the row, we controlled for the other variables listed here: participant gender, age, region, household income, employment status, education level, marital status, ethnicity, and the child's gender and school year
Abbreviations: CI = confidence interval

Associations between well-being and leaving home

Parents who perceived that their child had left the home for shopping, to provide help to someone else, to meet family, for medical treatment or for another reason had higher odds of reporting that their child had lower well-being (Table 6) [adjusted odds ratio, 1.24 (95% CI, 1.13 to 1.35); 1.37 (95% CI, 1.21 to 1.56); 1.19 (95% CI, 1.07 to 1.33); 3.70 (95% CI, 2.64 to 5.18); 1.22 (95% CI, 1.08 to 1.37), respectively]. Parents who perceived that their child was not leaving the home to exercise also had higher odds of reporting that their child had lower well-being [adjusted odds ratio, 0.93 (95% CI, 0.89 to 0.97)].

Table 6 Binary logistic regression comparing reasons for children leaving the home by children with low well-being (n=519)

Number of times that child had left the home for each reason on a continuous scale starting at 0	Child higher well-being, n, (%)	Child lower well-being, n, (%)	Unadjusted odds ratio (95%, CI)	P-value	Adjusted odds ratio (95%, CI) †	P-value
To go to the shops for groceries, toiletries, or medicines	N=1491, M=0.53, SD=1.03	N=519, M=0.83, SD=1.24	**1.25** (1.15 to 1.36)**	**<0.001**	**1.24** (1.13 to 1.35)**	**<0.001**
To go to the shops for other items	N=1491, M=0.38, SD=0.91	N=519, M=0.68, SD=1.13	**1.32** (1.20 to 1.45)**	**<0.001**	**1.31** (1.19 to 1.45)**	**<0.001**
To provide help to someone else	N=1491, M=0.15, SD=0.71	N=519, M=0.42, SD=0.99	**1.44** (1.27 to 1.64)**	**<0.001**	**1.37** (1.21 to 1.56)**	**<0.001**
To meet friends	N=1491, M=0.44, SD=1.51	N=519, M=0.64, SD=1.20	**1.14** (1.06 to 1.24)**	**<0.001**	**1.14* (1.05 to 1.24)**	**0.002**
To meet family members who don't live with you	N=1491, M=0.42, SD=0.88	N=519, M=0.59, SD=0.95	**1.22** (1.10 to 1.35)**	**<0.001**	**1.19** (1.07 to 1.33)**	**0.001**
For a medical need (e.g., an outpatient appointment)	N=1497, M=0.04, SD=0.25	N=519, M=0.24, SD=0.60	**3.75** (2.75 to 5.11)**	**<0.001**	**3.70** (2.64 to 5.18)**	**<0.001**
To go to school	N=1491, M=0.62, SD=1.48	N=519, M=0.77, SD=1.51	1.07 (1.00 to 1.14)	0.05	1.06 (0.99 to 1.13)	0.12
For exercise	N=1491, M=3.27, SD=2.99	N=519, M=2.64, SD=2.53	**0.92** (0.89 to 0.96)**	**<0.001**	**0.93** (0.89 to 0.97)**	**<0.001**
For another reason	N=1491, M=0.15, SD=0.76	N=519, M=0.33, SD=0.97	**1.25** (1.12 to 1.40)**	**<0.001**	**1.22** (1.08 to 1.37)**	**0.001**

* *p≤ ·05* and formatted bold
** *p≤ ·001* and formatted bold
Some results are rounded to 3 rather than 2 decimal places to distinguish between *p≤ ·05* and *p≤ ·001*
† When interpreting the predictor listed in the row, we controlled for the other variables listed here: participant gender, age, region, household income, employment status, education level, marital status, ethnicity, and the child's gender and school year

Abbreviations: n = number; M = mean; SD = standard deviation; % = percentage; CI = confidence interval

Discussion

During the COVID-19 pandemic, interventions were implemented to reduce physical contacts, including school closures and limits to physical contact between members of different households. We explored the impact of these measures to identify the risk factors for children having 1) close contact with family members from outside their household and 2) a low well-being.

Physical close contact with non-household family members

Our finding that 15% of children had close contact with non-household family members when schools were closed is concerning, as this would have increased the risk of disease transmission. This is particularly problematic for the 6% who had close contact with a non-household family member aged 70 years or over, who would be particularly at risk from COVID-19.

We found several variables associated with non-household contact that seem to point towards a high risk of such interactions among families that require childcare. This included, for example, when one parent was a key worker and when parents reported they had been unable to keep up with work or other important commitments. At the time of the survey parents were able to go back to work and some children were eligible to attend school. However, research suggests that most eligible children were still not attending school [21], and many will have needed alternative childcare.

We found that the risk for non-household family close contact was relatively equal across all children's ages, which contrasts with previous research [7]. We

suspect that the restrictions in place at the time of our survey explains this result. A longitudinal German study found that during COVID-19, the activities that children engaged in differed between ages [32]. However, these age differences disappeared when the guidance was more restrictive. That study also found that children commonly met elderly relatives (and friends) throughout the one-year study period, which supports our findings.

There appeared to be a cluster of predictive variables that indicated that children with worse psychological well-being had higher odds of meeting up with non-household family members. Variables in this group included children or their parent having low well-being and children being worried about non-household family members. We interpret these findings as the close contact may have been used to try to improve children's well-being [33], although as we are unable to determine causality; it could be that children's well-being reduced because of meeting their non-household family members.

Children who had extra support before the school closures and had special educational needs also had higher odds of non-household family close contact. This suggests that worries about education also increased risk of non-household close contact, which aligns with previous research [7].

Our results were less clear in terms of the perceived risk of COVID-19 and the impact on children's non-household family close contact. We did not find any associations between children's non-household family close contact and the lockdown statements that related to COVID-19. This finding is at odds with previous research [7]. In addition, children who were vulnerable to COVID-19 and lived with someone over 70 years old had higher odds of non-household family close contact.

These findings are concerning, suggesting a higher risk of contagion in these more vulnerable groups.

Children with a low well-being

Over a quarter (26%) of children reported on in our study had low well-being. A study in Switzerland conducted while schools were closed due to the COVID-19 pandemic found children's well-being and family functioning had reduced compared to before the pandemic, and the psychological impacts were greater for children at risk for neurodevelopmental impairments [34]. Children with special educational needs had four times higher odds of having a lower well-being compared to children without special educational needs. Previous research supports this finding [35]. In addition, a study suggests that children with special education needs are at risk for emotional, conduct, attention and peer relationship problems [36]. Therefore, the risk of adverse mental health problems could be exacerbated in children with special educational needs due to being at increased risk of challenging behaviour during lockdown. Parents who struggle to manage their children's behaviour are prone to using adverse parenting styles, and are at increased risk of family conflict and parental distress [37-39]. That said, 'special educational needs' covers a wide range of health and educational needs and research is needed to unpack what makes these children most at risk of poor well-being; increased risk could result from educational worries and a lack of academic support for these children [34].

We also observed risk factors for low well-being that relate to the home environment: parents who reported that their child had lower well-being also tended to report that they were bored or upset about not seeing family members, and that people in the household had not been getting along, or there was no structure to the

day. Parents also had higher odds of being worried about the financial impact of lockdown or unable to keep up with work and other commitments. Similar risk factors were identified in a study conducted during the COVID-19 pandemic that found individuals with poor sleep quality, increased distress due to financial circumstances, dependents, and or who were not adjusting to lockdown had higher odds of experiencing depression [40]. In addition, there is research showing the relationship between parent low well-being and child poor mental health outcomes [5, 37, 41]. We found that children were at seven times higher odds of lower well-being when the parent had low well-being. Our findings indicate that not only is parental well-being associated with child well-being but there also appears to be a link between parental distress due to home circumstances, such as financial worries.

Factors related to vulnerability to COVID-19 were also associated with children having lower well-being. These findings mirror research showing that risk factors for poor well-being in children include being worried about a grandparent's vulnerability to COVID-19, infecting their grandparents [15, 16] and being vulnerable to COVID-19 themselves [42, 43]. Children also had higher odds of a lower well-being if one of their parents was a key worker. Keyworkers commonly interacted with many people daily, which increased their risk of COVID-19 [44]. Families that adopted more protective behaviours because of the risk of COVID-19 also had higher odds of having a child with lower well-being; it is possible that increased levels of protective behaviours reflected a higher general sense of worry about the pandemic within the household.

More positively, children who had a higher well-being had higher odds of having parents who had confidence in home-schooling and who perceived that

children were keeping up with their schooling. Research has shown that parental self-efficacy can improve children's well-being [45]. It is possible that this explains our results perhaps by reducing tension within the home about schoolwork. We did not find any associations with parental education, income, or employment status in contrast to previous research [46] although we did note that younger parents had higher odds of reporting low well-being for their children than older parents. Younger adults in general experienced higher levels of stress and anxiety during the pandemic [40] because of more challenging working and living conditions, something which might account for our findings.

Exercise was also a protective factor; children had higher odds of having higher well-being the more times they had left the home to exercise. This finding aligns with previous research that shows the benefits of exercise on physical and mental health [40, 47-50]. We were surprised that access to outside space had no associations with either of our outcomes, although having access to outside space does not necessarily mean children use the space. To counterbalance the increased stress on families as a result of the pandemic, exercise should be promoted as a way to maintain well-being and parents should be taught how to increase their self-efficacy in managing difficult situations.

Limitations

We used a cross-sectional study design and the findings are based on a single point in time. Cross-sectional designs are common in health research as data can be gathered quickly so that the data can be used to respond rapidly to the health threat [51]. We used purposive sampling to meet pre-determined quotas for parent and children characteristics to broadly represent parents and children in England. The

use of quota rather than random sampling means that it is not possible to quantify the nature of any bias in the prevalence estimates that we have made. However, we have no reason to believe that the associations between the different variables that we measured would be affected by any theoretical bias [52]. Data were collected from self-reports, which can lead to self-report bias [53]. However, self-report data is a valid study design method [54, 55]. The RCADS is designed for children aged between 8 and 18 although parents reported on children from four years old [56]. However, we suggest this has minimal impact on our findings, RCADS has been found to be a reliable and valid measure in children as young as three years old [57].

Conclusions

During the COVID-19 pandemic we found that although children reduced their close contact, 15% had non-adherent physical contact with non-household family members. The reasons for close contact were largely related to a need for childcare, although factors relating to COVID-19, children's well-being and education were also important. Children who had special educational needs or had a parent with low well-being were most at risk for having a lower well-being themselves. Exercise and parent self-efficacy with home-schooling may help maintain children's mental and physical health.

Keywords: children; COVID-19; close contact; physical close contact; adherence; England; parents; well-being; mental health; physical health; exercise

Author contributions:

Lisa Woodland: Conceptualised, Methodology, Formal analysis, Investigation, Data Curation, Writing – Original Draft, Visualization, Project administration.

Louise E. Smith: Conceptualised, Methodology, Writing – Review & Editing.

Samantha K. Brooks: Methodology, Writing – Review & Editing.

Rebecca K. Webster: Conceptualised, Methodology, Writing – Review & Editing, Supervision, Funding acquisition.

Richard Amlôt: Conceptualised, Methodology, Writing – Review & Editing, Supervision, Funding acquisition.

Antonia Rubin: Investigation, Methodology, Writing – Review & Editing.

G. James Rubin: Conceptualised, Methodology, Resources, Writing – Review & Editing, Supervision, Funding acquisition.

Competing Interests: I have read the journal's policy and the authors of this manuscript have the following competing interests: G.J.R., R.A. and L.S. participate in the UK's Scientific Advisory Group for Emergencies, or its subgroups. RA is an employee of the UK Health Security Agency, which is an arm of the UK Government. This does not alter our adherence to PLOS ONE policies on sharing data and materials. These groups did not fund the study or authors.

Data Sharing: The data set and survey questions related to this study can be accessed via the King's College London research data repository, KORDS (DOI 10.18742/21757232). For further information contact: kcl - research.data research.data@kcl.ac.uk.

Appendix A

Table 7 Binary logistic regression comparing parent and child characteristics and associations with children's non-household close contact

Variable	Level	Child's not had close contact, n, (%)	Child's had close contact, n, (%)
Parent gender	Male	797 (86)	134 (14)
	Female	894 (84)	171 (16)
Parent age	18-35 years	305 (77)	89 (23)
	36-45 years	738 (85)	130 (15)
	46 years ≥	658 (88)	90 (12)
Region	East Midlands	129 (85)	22 (15)
	East of England	186 (85)	33 (15)
	North East	104 (88)	14 (12)
	North West	232 (85)	41 (15)
	South East	283 (84)	52 (16)
	South West	143 (88)	20 (12)

	West Midlands	183 (87)	28 (13)
	Yorkshire & the Humber	161 (83)	34 (17)
	London	280 (81)	65 (19)
Household income	≤ £34,999	660 (82)	144 (18)
	£35,000 ≥	958 (86)	156 (14)
Employment status[1]	Working	1412 (84)	265 (16)
	Not working	278 (87)	43 (13)
Parent Working from home[2]	Yes	774 (82)	166 (18)
	No	551 (88)	77 (12)
Education level	≤ A-level	740 (87)	115 (13)
	Degree ≥	947 (83)	192 (17)
Marital status	Living alone	262 (80)	65 (20)
	Married/cohabiting	1439 (86)	244 (14)
Ethnicity	White	1483 (85)	270 (15)
	BAME	203 (84)	38 (16)
Key worker status	Both parents	167 (86)	28 (14)
	One parent	686 (81)	161 (19)
	No	837 (88)	119 (12)
Child gender	Boy	891 (84)	172 (16)
	Girl	810 (86)	137 (14)
Child school year	Early Years	85 (84)	16 (16)
	Key Stage 1	279 (78)	77 (22)
	Key Stage 2	576 (85)	99 (15)
	Key Stage 3	334 (85)	61 (15)
	Key Stage 4	289 (88)	39 (12)
	Years 12 & 13	138 (89)	17 (11)
Child had special educational needs	Yes	118 (73)	43 (27)
	No	1568 (86)	264 (14)
Child lower well-being	Yes	384 (74)	135 (26)
	No	1317 (88)	174 (12)
Parent lower well-being	Yes	295 (75)	96 (25)
	No	1406 (87)	213 (13)
Child vulnerable COVID-19	Yes	111 (71)	46 (29)
	No	1566 (86)	260 (14)
Household vulnerable COVID-19	Yes	429 (84)	81 (16)
	No	1127 (86)	184 (14)
Someone over 70 years	Yes	59 (68)	28 (32)
	No	1642 (85)	281 (15)
COVID-19 behaviours followed by child and parent	Continuous (0=followed no behaviours, 11=followed all behaviours)	N=1701, M=5.51, SD=3.06	N=309, M=6.11, SD=3.27
Access to outside space[3]	Garden	1524 (85)	260 (15)
	Other[3]	121 (77)	36 (23)
	No	56 (81)	13 (19)

[1] Working includes students and volunteers
[2] Question only offered to participants who reported working in a paid job (full-time, part-time, and self-employed) and not to participants who reported being a student, on furlough and a volunteer.
[3] Participants that reported no access to a garden but had access to other outdoor spaces such as patio, terrace, and balcony
Abbreviations: n = number; % = percentage; M = mean; SD = standard deviation

Appendix B

Table 8 Binary logistic regression comparing statements about lockdown and associations with children's non-household close contact. Close contact is defined by a child's close contact with a family member outside the household (n=309)

Statement	Level	Child's not had close contact, n, (%)	Child's had close contact, n, (%)
If child goes out, she/he is likely to catch coronavirus	5-point Likert-scale (1=strongly agree, 5=strongly disagree)	N=1672, M=3.27, SD=1.03	N=309, M=3.08, SD=1.18
If child goes out, she/he is likely to bring coronavirus back into our home	5-point Likert-scale (1=strongly agree, 5=strongly disagree)	N=1672, M=3.23, SD=1.07	N=309, M=3.05, SD=1.16
Child is keeping up with his/her schoolwork	5-point Likert-scale (1=strongly agree, 5=strongly disagree)	N=1672, M=2.12, SD=1.08	N=309, M=2.30, SD=1.15
I feel confident helping child with her/his schoolwork	5-point Likert-scale (1=strongly agree, 5=strongly disagree)	N=1672, M=2.23, SD=1.03	N=305, M=2.30, SD=1.07
I feel supported by child's school	5-point Likert-scale (1=strongly agree, 5=strongly disagree)	N=1681, M=2.32, SD=1.07	N=306, M=2.32, SD=1.07
I have access to all the resources that child needs to do her/his schoolwork	5-point Likert-scale (1=strongly agree, 5=strongly disagree)	N=1678, M=2.15, SD=1.02	N=307, M=2.21, SD=1.03
During lockdown, child has learned about important things she/he wouldn't normally learn at school	5-point Likert-scale (1=strongly agree, 5=strongly disagree)	N=1691, M=2.40, SD=1.00	N=308, M=2.38, SD=1.05
In the past 7 days, child has been bored	5-point Likert-scale (1=strongly agree, 5=strongly disagree)	N=1694, M=2.54, SD=1.20	N=309, M=2.30, SD=1.16
In the past 7 days, my household has had a regular structure to the day	5-point Likert-scale (1=strongly agree, 5=strongly disagree)	N=1692, M=2.31, SD=1.02	N=309, M=2.43, SD=1.12
In the past 7 days, child has kept in touch with her/his friends	5-point Likert-scale (1=strongly agree, 5=strongly disagree)	N=1691, M=2.25, SD=1.14	N=307, M=2.26, SD=1.12
Child is worried about coronavirus	5-point Likert-scale (1=strongly agree, 5=strongly disagree)	N=1690, M=2.72, SD=1.12	N=306, M=2.70, SD=1.23
In the past 7 days, child has felt upset about not seeing other family members who do not live with us	5-point Likert-scale (1=strongly agree, 5=strongly disagree)	N=1684, M=2.97, SD=1.24	N=308, M=2.65, SD=1.25
In the past 7 days, I have found it hard to keep up with work or other important commitments	5-point Likert-scale (1=strongly agree, 5=strongly disagree)	N=1626, M=3.22, SD=1.21	N=299, M=2.79, SD=1.21
In the past 7 days, people in my household have been getting along well	5-point Likert-scale (1=strongly agree, 5=strongly disagree)	N=1691, M=2.08, SD=0.95	N=309, M=2.19, SD=1.01
I am worried about the financial impact of lockdown measures	5-point Likert-scale (1=strongly agree, 5=strongly disagree)	N=1685, M=2.51, SD=1.21	N=304, M=2.40, SD=1.16
Before the school closures, child had extra support at school	5-point Likert-scale (1=strongly agree, 5=strongly disagree)	N=1528, M=3.59, SD=1.36	N=285, M=3.16, SD=1.48

Abbreviations: n = number; % = percentage; M = mean; SD = standard deviation

Appendix C

Table 9 Frequencies to show the behaviours that parents or children had followed, in the past 7 days because of the risk of coronavirus. The findings our presented by children's non-household family close contact (n=309) and lower well-being (n=519).

Statements	Statement in relation to COVID-19 guidelines at the time	Total participants (n=2010, %)	Childs not had close contact (n=1701, %)	Childs had close contact (n=309, (%)	Child higher well-being, (n=1491, %)	Child lower well-being, (n=519, %)
Washed your hands thoroughly and regularly	Recommended	1895 (94%)	1617 (95)	278 (90)	1435 (96)	460 (89)
Stayed 2m (3 steps) away from people you do not live with when outside your home	Recommended	1867 (93%)	1599 (94)	268 (87)	1413 (95)	454 (87)
Cleaned or disinfected items you have brought into the home, such as groceries or parcels	Not recommended	1089 (54%)	908 (53)	181 (59)	779 (52)	310 (60)
Washed [CHILD]'s clothes when she/ he has returned home	Not recommended	1089 (54%)	829 (49)	176 (57)	688 (46)	317 (61)
Washed your clothes when you have returned home	Not recommended	959 (48%)	788 (46)	171 (55)	653 (44)	306 (59)
Used tissues or clothing to touch door handles or other objects when outside	Not recommended	954 (48%)	788 (46)	166 (54)	679 (46)	275 (53)
Covered your face or used a face mask when out and about	Not recommended	822 (41%)	678 (40)	144 (47)	571 (38)	251 (48)
Covered [CHILD's] face or given her/him a face mask when out and about	Not recommended	741 (37%)	600 (35)	141 (46)	500 (34)	241 (46)
Left items that you have brought into the home for 24 hours or more before using them, to kill any virus on them	Not recommended	725 (36%)	607 (36)	118 (38)	493 (33)	232 (45)
Worn protective gloves when out and about	Not recommended	693 (35%)	562 (33)	131 (43)	453 (30)	240 (46)
Made [CHILD] wear protective gloves when out and about	Not recommended	503 (25%)	390 (23)	113 (37)	308 (21)	195 (38)

Abbreviations: n = number of parents; % = percentage

Appendix D

Table 10 Binary logistic regression comparing parent and child characteristics and associations with children's lower well-being (n=519)

Variable	Level	Child higher well-being, n, (%)	Child lower well-being, n, (%)
Parent gender	Male	702 (75)	229 (25)

	Female	784 (74)	281 (26)
Parent age	18-35 years	263 (67)	131 (33)
	36-45 years	652 (75)	216 (25)
	46 years ≥	576 (77)	172 (23)
Region	East Midlands	101 (67)	50 (33)
	East of England	163 (74)	56 (26)
	North East	92 (78)	26 (22)
	North West	208 (76)	65 (24)
	South East	248 (74)	87 (26)
	South West	118 (72)	45 (28)
	West Midlands	161 (76)	50 (24)
	Yorkshire & the Humber	152 (78)	43 (22)
	London	248 (72)	97 (28)
Household income	≤ £34,999	583 (73)	221 (27)
	£35,000 ≥	832 (75)	282 (25)
Employment status[1]	Working	1240 (74)	437 (26)
	Not working	244 (76)	77 (24)
Parent Working from home[2]	Yes	682 (73)	258 (27)
	No	474 (75)	154 (25)
Education level	≤ A-level	642 (75)	213 (25)
	Degree ≥	836 (73)	303 (27)
Marital status	Living alone	234 (72)	93 (28)
	Married/cohabiting	1257 (75)	426 (25)
Ethnicity	White	1304 (74)	449 (26)
	BAME	176 (73)	65 (27)
Key worker status	Both parents	134 (69)	61 (31)
	One parent	593 (70)	254 (30)
	No	754 (79)	202 (21)
Child gender	Boy	771 (73)	292 (27)
	Girl	720 (76)	227 (24)
Child school year	Early Years	77 (76)	24 (24)
	Key Stage 1	263 (74)	93 (26)
	Key Stage 2	517 (7)	158 (23)
	Key Stage 3	286 (72)	109 (28)
	Key Stage 4	230 (70)	98 (30)
	Years 12 & 13	118 (76)	37 (24)
Child has special educational needs	Yes	71 (44)	90 (56)
	No	1410 (770	422 (23)
Parent low well-being	Yes	159 (41)	232 (59)
	No	1332 (82)	287 (18)
Child vulnerable COVID-19	Yes	77 (49)	80 (51)
	No	1393 (76)	433 (24)
Household vulnerable COVID-19	Yes	329 (65)	181 (35)
	No	1029 (78)	282 (22)
Someone over 70 years	Yes	46 (53)	41 (47)
	No	1445 (75)	478 (25)
COVID-19 behaviours followed by child and parent	Continuous (0=followed no behaviours, 11=followed all behaviours)	N=1491, M=5.35, SD=3.02	N=519, M=6.32, SD=3.22
Access to outside space[3]	Garden	1333 (75)	451 (25)
	Other[3]	108 (69)	49 (31)
	No	50 (72)	19 (28)

* *p*≤ *·05* and formatted bold

** *p*≤ *·001* and formatted bold

Some results are rounded to 3 rather than 2 decimal places to distinguish between *p*≤ *·05* and *p*≤ *·001*

† When interpreting the predictor listed in the row, we controlled for the other variables listed here: participant gender, age, region, household income, employment status, education level, marital status, ethnicity, and the child's gender and school year

^^^ Not included variable highly correlated with employment variable

[1] Working includes students and volunteers
[2] Question only offered to participants who reported working in a paid job (full-time, part-time, and self-employed) and not to participants who reported being a student, on furlough and a volunteer.
[3] Participants that reported no access to a garden but had access to other outdoor spaces such as patio, terrace, and balcony
Abbreviations: n = number of parents; % = percentage; CI = confidence interval

Appendix E

Table 11 Binary logistic regression comparing statements about lockdown and associations with children's lower well-being (n=519)

Statement	Level	Child higher well-being, n, (%)	Child lower well-being, n, (%)
If child goes out, she/he is likely to catch coronavirus	5-point Likert-scale (1=strongly agree, 5=strongly disagree)	N=1473, M=3.37, SD=1.02	N=515, M=2.87, SD=1.06
If child goes out, she/he is likely to bring coronavirus back into our home	5-point Likert-scale (1=strongly agree, 5=strongly disagree)	N=1465, M=3.33, SD=1.04	N=515, M=2.82 SD=1.11
Child is keeping up with his/her schoolwork	5-point Likert-scale (1=strongly agree, 5=strongly disagree)	N=1470, M=2.03, SD=1.01	N=508, M=2.48, SD=1.24
I feel confident helping child with her/his schoolwork	5-point Likert-scale (1=strongly agree, 5=strongly disagree)	N=1469, M=2.16, SD=0.98	N=508, M=2.48, SD=1.13
I feel supported by child's school	5-point Likert-scale (1=strongly agree, 5=strongly disagree)	N=1475, M=2.25, SD=1.03	N=512, M=2.52 SD=1.15
I have access to all the resources that child needs to do her/his schoolwork	5-point Likert-scale (1=strongly agree, 5=strongly disagree)	N=1473, M=2.09, SD=0.97	N=512, M=2.37, SD=1.14
During lockdown, child has learned about important things she/he wouldn't normally learn at school	5-point Likert-scale (1=strongly agree, 5=strongly disagree)	N=1481, M=2.37, SD=0.98	N=518, M=2.49, SD=1.08
In the past 7 days, child has been bored	5-point Likert-scale (1=strongly agree, 5=strongly disagree)	N=1486, M=2.63, SD=1.20	N=517, M=2.14, SD=1.11
In the past 7 days, my household has had a regular structure to the day	5-point Likert-scale (1=strongly agree, 5=strongly disagree)	N=1484, M=2.27, SD=0.99	N=517, M=2.50, SD=1.14
In the past 7 days, child has kept in touch with her/his friends	5-point Likert-scale (1=strongly agree, 5=strongly disagree)	N=1483, M=2.21, SD=1.12	N=515, M=2.39, SD=1.19
Child is worried about coronavirus	5-point Likert-scale (1=strongly agree, 5=strongly disagree)	N=1481, M=2.88, SD=1.12	N=515, M=2.27, SD=1.08
In the past 7 days, child has felt upset about not seeing other family members who do not live with us	5-point Likert-scale (1=strongly agree, 5=strongly disagree)	N=1475, M=3.09, SD=1.24	N=517, M=2.45, SD=1.15
In the past 7 days, I have found it hard to keep up with work or other important commitments	5-point Likert-scale (1=strongly agree, 5=strongly disagree)	N=1429, M=3.37, SD=1.19	N=496, M=2.55, SD=1.15
In the past 7 days, people in my household have been getting along well	5-point Likert-scale (1=strongly agree, 5=strongly disagree)	N=1484, M=2.00, SD=0.93	N=516, M=2.37, SD=0.97

I am worried about the financial impact of lockdown measures	5-point Likert-scale (1=strongly agree, 5=strongly disagree)	N=1475, M=2.58, SD=1.23	N=514, M=2.24, SD=1.10
Before the school closures, child had extra support at school	5-point Likert-scale (1=strongly agree, 5=strongly disagree)	N=1326, M=3.72, SD=1.32	N=487, M=2.98, SD=1.42

Abbreviations: N = number of parents; M = mean; SD = standard deviation; % = percentage

References

1. HM Government Prime Minister's statement on coronavirus (COVID-19): 23 March 2020. https://www.gov.uk/government/speeches/pm-address-to-the-nation-on-coronavirus-23-march-2020 (accessed 11 June 2020).

2. HM Government Children of critical workers and vulnerable children who can access schools or educational settings. https://www.gov.uk/government/publications/coronavirus-covid-19-maintaining-educational-provision/guidance-for-schools-colleges-and-local-authorities-on-maintaining-educational-provision (accessed 16 March 2021).

3. HM Government Prime Minister's statement on coronavirus (COVID-19): 11 May 2020. https://www.gov.uk/government/speeches/pm-statement-on-coronavirus-11-may-2020 (accessed 22 March 2022).

4. HM Government, PM: Six people can meet outside under new measures to ease lockdown. 2020.

5. Viner, R.; Russell, S.; Saulle, R.; Croker, H.; Stansfield, C.; Packer, J.; Nicholls, D.; Goddings, A.-L.; Bonell, C.; Hudson, L.; Hope, S.; Ward, J.; Schwalbe, N.; Morgan, A.; Minozzi, S., School Closures During Social Lockdown and Mental Health, Health Behaviors, and Well-being Among Children and Adolescents During the First COVID-19 Wave. *JAMA Pediatrics* **2022**.

6. Woodland, L.; Hodson, A.; Webster, R. K.; Amlôt, R.; Smith, L. E.; Rubin, G. J., A qualitative study about how families coped with managing their well-being, children’s physical activity and education during the COVID-19 school closures in England. *PLOS ONE* **2022,** *17* (12), e0279355.

7. Brooks, S. K.; Smith, L. E.; Webster, R. K.; Weston, D.; Woodland, L.; Hall, I.; Rubin, G. J., The impact of unplanned school closure on children's social contact: rapid evidence review. *Euro Surveill* **2020,** *25* (13).

8. Mizumoto, K.; Yamamoto, T.; Nishiura, H., Contact behaviour of children and parental employment behaviour during school closures against the pandemic influenza A (H1N1-2009) in Japan. *Journal of International Medical Research* **2013,** *41* (3), 716-724.

9. DiMatteo, M. R., The role of effective communication with children and their families in fostering adherence to pediatric regimens. *Patient Education and Counseling* **2004,** *55* (3), 339-344.

10. Kunin-Batson, A. S.; Seburg, E. M.; Crain, A. L.; Jaka, M. M.; Langer, S. L.; Levy, R. L.; Sherwood, N. E., Household factors, family behavior patterns, and adherence to dietary and physical activity guidelines among children at risk for obesity. *Journal of nutrition education and behavior* **2015,** *47* (3), 206-215. e1.

11. Smith, L. E.; D'Antoni, D.; Jain, V.; Pearce, J. M.; Weinman, J.; Rubin, G. J., A systematic review of factors affecting intended and actual adherence with antiviral medication as treatment or prophylaxis in seasonal and pandemic flu. *Influenza and other respiratory viruses* **2016,** *10* (6), 462-478.

12. Gentili, D.; Bardin, A.; Ros, E.; Piovesan, C.; Ramigni, M.; Dalmanzio, M.; Dettori, M.; Filia, A.; Cinquetti, S., Impact of Communication Measures Implemented During a School Tuberculosis Outbreak on Risk Perception among Parents and School Staff, Italy, 2019. *International Journal of Environmental Research and Public Health* **2020,** *17* (3), 911.

13. Woodland, L.; Hodson, A.; Webster, R. K.; Amlôt, R.; Smith, L. E.; Rubin, G. J., A Qualitative Study Evaluating the Factors Affecting Families' Adherence to the First COVID-19 Lockdown in England Using the COM-B Model and TDF. *International Journal of Environmental Research and Public Health* **2022,** *19* (12), 7305.

14. HM Government *Sixteenth SAGE meeting on Wuhan Coronavirus (Covid-19), 16th March 2020*; 2020.

15. Sarkadi, A.; Sahlin Torp, L.; Pérez-Aronsson, A.; Warner, G., Children's expressions of worry during the COVID-19 pandemic in Sweden. *Journal of Pediatric Psychology* **2021,** *46* (8), 939-949.

16. Idoiaga, N.; Berasategi, N.; Eiguren, A.; Picaza, M., Exploring children's social and emotional representations of the Covid-19 pandemic. *Frontiers in Psychology* **2020,** *11*, 1952.

17. Denford, S.; Morton, K. S.; Lambert, H.; Zhang, J.; Smith, L. E.; Rubin, G. J.; Cai, S.; Zhang, T.; Robin, C.; Lasseter, G.; Hickman, M.; Oliver, I.; Yardley, L., Understanding patterns of adherence to COVID-19 mitigation measures: a qualitative interview study. *Journal of Public Health* **2021,** *43* (3), 508-516.

18. Lasseter, G.; Compston, P.; Robin, C.; Lambert, H.; Hickman, M.; Denford, S.; Reynolds, R.; Zhang, J.; Cai, S.; Zhang, T.; Smith, L. E.; Rubin, J.; Yardley, L.; Amlot, R.; Oliver, I., Exploring the impact of shielding advice on the health and wellbeing of individuals identified as extremely vulnerable and advised to shield in Southwest England amid the COVID-19 pandemic: A mixed-methods evaluation. Cold Spring Harbor Laboratory: 2022.

19. BMG Research BMG About Us. https://www.bmgresearch.co.uk/bmg/about-us/ (accessed 1 September 2022).

20. Smith, L. E.; Woodland, L.; Amlôt, R.; Rubin, A.; Rubin, G. J., A cross-sectional survey of parental perceptions of COVID-19 related hygiene measures within schools and adherence to social distancing in journeys to and from school. *BMJ Paediatrics Open* **2020,** *4* (1).

21. Woodland, L.; Smith, L. E.; Webster, R. K.; Amlôt, R.; Rubin, A.; Wessely, S.; Rubin, G. J., Why did some parents not send their children back to school following school closures during the COVID-19 pandemic: a cross-sectional survey. *BMJ Paediatrics Open* **2021,** *5* (1), e001014.

22. Zhang, C.; Conrad, F. In *Speeding in web surveys: The tendency to answer very fast and its association with straightlining*, Survey research methods, 2014; pp 127-135.

23. Callegaro, M.; DiSogra, C., Computing Response Metrics for Online Panels. *Public Opinion Quarterly* **2008,** *72* (5), 1008-1032.

24. HM Government Schools, pupils and their characteristics: Academic Year 2019/20. https://explore-education-statistics.service.gov.uk/find-statistics/school-pupils-and-their-characteristics (accessed 25 June 2020).

25. HM Government [Withdrawn] Guidance for contacts of people with confirmed coronavirus (COVID-19) infection who do not live with the person. https://www.gov.uk/government/publications/guidance-for-contacts-of-people-with-possible-or-confirmed-coronavirus-covid-19-infection-who-do-not-live-with-the-person/guidance-for-contacts-of-people-with-possible-or-confirmed-coronavirus-covid-19-infection-who-do-not-live-with-the-person (accessed 16 August 2023).

26. Chorpita, B. F.; Yim, L.; Moffitt, C.; Umemoto, L. A.; Francis, S. E., Assessment of symptoms of DSM-IV anxiety and depression in children: a revised child anxiety and depression scale. *Behaviour Research and Therapy* **2000,** *38* (8), 835-855.

27. Kroenke, K.; Spitzer, R. L.; Williams, J. B. W.; Lowe, B., An Ultra-Brief Screening Scale for Anxiety and Depression: The PHQ-4. *Psychosomatics* **2009,** *50* (6), 613-621.

28. Piqueras, J. A.; Martín-Vivar, M.; Sandin, B.; San Luis, C.; Pineda, D., The Revised Child Anxiety and Depression Scale: A systematic review and reliability generalization meta-analysis. *Journal of affective disorders* **2017,** *218*, 153-169.

29. Wicke, F. S.; Krakau, L.; Löwe, B.; Beutel, M. E.; Brähler, E., Update of the standardization of the Patient Health Questionnaire-4 (PHQ-4) in the general population. *Journal of Affective Disorders* **2022,** *312*, 310-314.

30. IBM Corp IBM SPSS Statistics for Windows, 26.0 Armonk, NY.

31. IBM Corp IBM SPSS Statistics for Windows, 28.0 Armonk, NY.

32. Paulsen, M.; Zychlinsky Scharff, A.; De Cassan, K.; Sugianto, R. I.; Blume, C.; Blume, H.; Christmann, M.; Hauß, C.; Illig, T.; Jonczyk, R.; Klopp, N.; Kopfnagel, V.; Lichtinghagen, R.; Lucas, H.; Luhr, A.; Mutschler, F.; Pietschmann,

T.; Pott, P.-C.; Prokein, J.; Schaefer, P.; Stahl, F.; Stanislawski, N.; Von Der Born, J.; Schmidt, B. M. W.; Heiden, S.; Stiesch, M.; Memaran, N.; Melk, A., Children and Adolescents' Behavioral Patterns in Response to Escalating COVID-19 Restriction Reveal Sex and Age Differences. *Journal of Adolescent Health* **2022,** *70* (3), 378-386.

33. Morina, N.; Kip, A.; Hoppen, T. H.; Priebe, S.; Meyer, T., Potential impact of physical distancing on physical and mental health: a rapid narrative umbrella review of meta-analyses on the link between social connection and health. *BMJ Open* **2021,** *11* (3), e042335.

34. Ehrler, M.; Werninger, I.; Schnider, B.; Eichelberger, D. A.; Naef, N.; Disselhoff, V.; Kretschmar, O.; Hagmann, C. F.; Latal, B.; Wehrle, F. M., Impact of the COVID-19 pandemic on children with and without risk for neurodevelopmental impairments. *Acta Paediatrica* **2021,** *110* (4), 1281-1288.

35. Essex, M. J.; Kraemer, H. C.; Armstrong, J. M.; Boyce, W. T.; Goldsmith, H. H.; Klein, M. H.; Woodward, H.; Kupfer, D. J., Exploring Risk Factors for the Emergence of Children's Mental Health Problems. *Archives of General Psychiatry* **2006,** *63* (11), 1246.

36. Deighton, J.; Lereya, S. T.; Casey, P.; Patalay, P.; Humphrey, N.; Wolpert, M., Prevalence of mental health problems in schools: poverty and other risk factors among 28 000 adolescents in England. *British Journal of Psychiatry* **2019,** *215* (3), 565-567.

37. Wille, N.; Bettge, S.; Ravens-Sieberer, U., Risk and protective factors for children's and adolescents' mental health: results of the BELLA study. *European Child & Adolescent Psychiatry* **2008,** *17* (S1), 133-147.

38. Prime, H.; Wade, M.; Browne, D. T., Risk and resilience in family well-being during the COVID-19 pandemic. *American Psychologist* **2020,** *75* (5), 631.

39. Spinelli, M.; Lionetti, F.; Pastore, M.; Fasolo, M., Parents' stress and children's psychological problems in families facing the COVID-19 outbreak in Italy. *Frontiers in psychology* **2020,** *11*, 1713.

40. Varma, P.; Junge, M.; Meaklim, H.; Jackson, M. L., Younger people are more vulnerable to stress, anxiety and depression during COVID-19 pandemic: A global cross-sectional survey. *Progress in Neuro-Psychopharmacology and Biological Psychiatry* **2021,** *109*, 110236.

41. Davis, E.; Sawyer, M. G.; Lo, S. K.; Priest, N.; Wake, M., Socioeconomic risk factors for mental health problems in 4–5-year-old children: Australian population study. *Academic Pediatrics* **2010,** *10* (1), 41-47.

42. Tunçgenç, B.; Newson, M.; Sulik, J.; Zhao, Y.; Dezecache, G.; Deroy, O.; Zein, M. E., Social alignment matters: Following pandemic guidelines is associated with better wellbeing. *BMC Public Health* **2022,** *22* (1).

43. Saurabh, K.; Ranjan, S., Compliance and Psychological Impact of Quarantine in Children and Adolescents due to Covid-19 Pandemic. *The Indian Journal of Pediatrics* **2020,** *87* (7), 532-536.

44. Environmental Modelling Group (EMG) Transmission Group *COVID-19 Risk by Occupation and Workplace*; HM Government: 11 February, 2021.

45. Albanese, A. M.; Russo, G. R.; Geller, P. A., The role of parental self-efficacy in parent and child well-being: A systematic review of associated outcomes. *Child: care, health and development* **2019,** *45* (3), 333-363.

46. Reiss, F., Socioeconomic inequalities and mental health problems in children and adolescents: A systematic review. *Social Science & Medicine* **2013,** *90*, 24-31.

47. Biddle, S. J. H.; Ciaccioni, S.; Thomas, G.; Vergeer, I., Physical activity and mental health in children and adolescents: An updated review of reviews and an analysis of causality. *Psychology of Sport and Exercise* **2019,** *42*, 146-155.

48. Ahn, S.; Fedewa, A. L., A Meta-analysis of the Relationship Between Children's Physical Activity and Mental Health. *Journal of Pediatric Psychology* **2011,** *36* (4), 385-397.

49. Eddolls, W. T. B.; McNarry, M. A.; Lester, L.; Winn, C. O. N.; Stratton, G.; Mackintosh, K. A., The association between physical activity, fitness and body mass index on mental well-being and quality of life in adolescents. *Quality of Life Research* **2018,** *27* (9), 2313-2320.

50. Benzing, V.; Gaillard, P.; Scheidegger, D.; Dössegger, A.; Nigg, C. R.; Schmidt, M., COVID-19: Physical Activity and Quality of Life in a Sample of Swiss School Children during and after the First Stay-at-Home. *International Journal of Environmental Research and Public Health* **2022,** *19* (4), 2231.

51. Wang, X.; Cheng, Z., Cross-sectional studies: strengths, weaknesses, and recommendations. *Chest* **2020,** *158* (1), S65-S71.

52. Kohler U Possible Uses of Nonprobability Sampling for the Social Sciences. *Survey Methods: Insights from the Field*. https://surveyinsights.org/?p=10981 (accessed 29 July 2020).

53. Epstein, W. M., Response bias in opinion polls and American social welfare. *The Social Science Journal* **2006,** *43* (1), 99-110.

54. Kormos, C.; Gifford, R., The validity of self-report measures of proenvironmental behavior: A meta-analytic review. *Journal of Environmental Psychology* **2014,** *40*, 359-371.

55. Craig, K.; Hale, D.; Grainger, C.; Stewart, M. E., Evaluating metacognitive self-reports: systematic reviews of the value of self-report in metacognitive research. *Metacognition and Learning* **2020,** *15*, 155-213.

56. Ebesutani, C.; Chorpita, B. F.; Higa-Mcmillan, C. K.; Nakamura, B. J.; Regan, J.; Lynch, R. E., A Psychometric Analysis of the Revised Child Anxiety and

Depression Scales—Parent Version in a School Sample. *Journal of Abnormal Child Psychology* **2011,** *39* (2), 173-185.

57. Ebesutani, C.; Tottenham, N.; Chorpita, B., The Revised Child Anxiety and Depression Scale - Parent Version: Extended Applicability and Validity for Use with Younger Youth and Children with Histories of Early-Life Caregiver Neglect. *Journal of Psychopathology and Behavioral Assessment* **2015,** *37* (4), 705-718.

Supporting information:

S1 Text. **Full survey material.** The information can be downloaded at: DOI 10.18742/21757232
