# Supplementary material for: Parent-reported child’s close contact with non-household family members and their well-being during the COVID-19 pandemic: A cross-sectional survey
Source: PLoS One. 2023 Oct 19;18(10):e0292344. doi: 10.1371/journal.pone.0292344 (PMC10586646; doi:10.1371/journal.pone.0292344)
Supplement: Attachment — Submitted filename: Editor comments.docx [file pone.0292344.s004.docx]

**Academic Editor’s comments**

Dear Dr. Woodland,

I would first like to apologize for the long review process. I had to wait for the 3rd reviewer's comments since Reviewer 1 had recommended acceptance, while Reviewer 2 had recommended a rejection.

While I felt that the article reports important findings, the methodology and results sections have watered them down. Two of the reviewers have raised critical points regarding your article. In fact, Reviewer 2 recommended a rejection. However, I has to carefully consider if this was worth another chance or I should also reject it. After careful consideration, I am of the view of giving you and your team a chance to carefully attend to the points raised by the reviewers and myself and resubmit. The main concern is on the statistical analysis, presentation and interpretation of the results, as you will note in my comments as well as those of the reviewers.

However, note that once you have resubmitted, I will have to subject the revised manuscript to another peer review cycle as I believe the manuscript would have had major changes due to the nature of the recommended suggestions/revisions. If you resubmit the paper without changes in the analysis and interpretation of the results, it will result into a rejection without peer review.

Regards,

Mduduzi C. Shongwe, PhD

Academic Editor, PLOS ONE

------------------------------------------------------------------------------

**Title:**

1. Since the children were not the respondents in this survey, the title should be rephrased to reflect that. Secondly, substitute “interaction” with “close contact” for consistency. The suggested/rephrased title is: “Perceived children’s close contact with non-household family members and their well-being during the COVID-19 pandemic: a cross-sectional survey”

**Abstract**:

2. Line 28: Is close contact = interaction? It is not clear. Be consistent throughout the article.
3. Line 32: mention the setting and highest statistical procedure/tests performed.
4. Lines 34-35: Include results of direct reports of interactions/contact by the students as well, not only as perceived by their parents.
5. Line 34: Revisit the statistical language: odds ratios are not a measure of risk, therefore, revise the phrase “more likely” to “had higher odds” throughout the manuscript.
6. Liness 34-40: state the statistical values e.g. aORs and their Cis.
7. Include a sentence that will serve as a “conclusion and recommendation” in the abstract.
8. Include key words after the abstract.

**Introduction**

9. Do not number the headings for PLOS One (revise the manuscript by strictly following the journal formatting style)
10. Line 54 should be “Box 1”. However, it is not clear what this text appears in a box when it is still the authors presenting their introduction. It would make sense if it was an image from another source document e.g., the media or regulating agency. I suggest this be removed from the box and be presented as part of the continuous writing of the introduction and be cited as done in the box.
11. Lines 100-104, why these variables, specifically, amongst all other 'risk factors'? What informed this selection?
12. Discuss the known risk factors from previous studies in the introduction section. What factors have other studies identified? Any disagreements/mixed findings on these?
13. Discuss the “well-being” reported in other studies from elsewhere as part of the literature review in the introduction section.

**Methods**:

14. Lines 106 and 117, BMG stands for?
15. Line 122, if you begin a sentence with a number, write the number in words. Do that throughout the manuscript.
16. Lines 124-125, “… completing the survey quickly…”. – What was the definition of “quickly”? Who assessed the quickness, BMG or the authors? How did the authors ascertain this?
17. Lines 124-126, indicate to readers whether this practice is common/conventional in such studies i.e. cite previous studies that have used or textbooks that recommend such practice.
18. Line 132, upload the Ethical clearance certificate during the resubmission.
19. Line 134, was the questionnaire/survey administered online or physically? On average, how long did it take participants to complete the survey?
20. Line 147, decide whether to use “interaction” or “close contact”? Be consistent.
21. Line 155, what is the validity of this question in measuring “physical interaction” or close contact?
22. Lines 156-165, discuss the scoring, validity and reliability of the measure. In fact, do that for all the measures in your study. Merge 2.5 with 2.3 (Lines 192-236).
23. Line 179, where were these questions sourced from? Or were they developed for this study? How or informed by?
24. Line 186, Who is the “patient” being referred to here?
25. Lines 192-236, “Kill” this section by merging it with 2.3: analysis should be discussed under 2.6. This will enhance the flow.
26. Lines 197-200, what informed this definition of close contact or interaction?
27. Lines 213-215, recoded from what original categorization? Or from continuous measurement? Specify that for all the variables described as "recoded" below.
28. Lines 229-232, which ones are these now? Are they the same questions referred-to in 2.3.6? If so, merge the sections with 2.3.6. If different, which ones are these now? What questions were asked? How were they developed? What is their validity?
29. Lines 212-236, these should be discussed under 2.3.
30. Line 233, what was the scoring and the possible score range? Merge/discuss this under 2.3.
31. Lines 235-236, what informed this statistical decision? Why were they not valid responses? Is this common statistical practice? If so, site the authorities or sources recommending such a practice. How many of such observations were deemed as missing? Did you assess the impact of removing these observations?
32. Line 236, so, what was the eventual response rate in this study?
33. Line 240, it appears as though hierarchical models would have been the best choice/more efficient here to minimize/control Type I error. Reconsider your analysis decisions/choices and justify your decisions or choices. Otherwise, rerun the analysis using hierarchical models.

**Results:**

34. Line 248, present the participants' background characteristics first.
35. Line 249-250, you cannot report the findings as though they were stated by the children. In such studies, indicate that the results were reported or perceived by the parents, e.g., here, it must read, ".... 15% of the children were reported to have had ......".
36. Line 256, as indicated in the comments for the abstract section, revisit your statistical language. Using “more likely” is not accurate reporting for odds ratios as they are not a measure of risk. Revise all such words and instead refer to them as "higher odds" or "lower odds" as appropriate. Do that through throughout the results and discussion sections.
37. Ordinarily, this should be a purely descriptive table of the background characteristics of the participants.
38. Throughout the results section, report the ORs and their CIs in text when reporting the results from regression.
39. Indicate whether it is crude or adjusted (i.e Bivariate or multiple). Do that for all tables
40. Show the "N" for all the tables.
41. Line 269, ordinarily, this should be a purely descriptive table of the background characteristics of the participants.
42. Line 274, it is not accurate to state that all these were adjusted for, yet they were also the predictors. Presenting such a statement implies that there was one or two main independent variable(s) and the rest were control variable/confounders, in which case, you would not interpret them. However, in your study, these were all covariates or predictors, hence, when interpreting one predictor, you should state that you controlled for the rest; not that the model adjusted for all the variables listed. Revist this.
43. Lines, 292-294, the correct presentation should be: "parents aged between 18 and 35 years old, parents who were a key worker and parents with lower well-being had higher odds of reporting that their child had lower well-being." You must do this correction throughout the results section and discussion section.
44. The Tables needs to be simplified to make them easier to read. Present fewer columns, by separately presenting the background characteristics, then descriptives of the variables in Tables 4 and 5 (as Supplementary files/Supporting Information) and collapse Tables 4 and 5 as suggested below.
45. Tables 4 & 5, is it possible to create composite scores of each variable and present them rather than ‘dumping’ all the individual items into the regression models, which is what appears to be the case in the current presentation. That is, these would then be two variables represented each table.

**Discussion**

46. The discussion section will have to be revised to align with the changes suggested above
